# HIF2α promotes tumour growth in clear cell renal cell carcinoma by increasing the expression of NUDT1 to reduce oxidative stress

**DOI:** 10.1002/ctm2.592

**Published:** 2021-11-04

**Authors:** Jian Shi, Zhiyong Xiong, Keshan Wang, Changfei Yuan, Yu Huang, Wen Xiao, Xiangui Meng, Zhixian Chen, Qingyang Lv, Daojia Miao, Huageng Liang, Tianbo Xu, Kairu Xie, Hongmei Yang, Xiaoping Zhang

**Affiliations:** ^1^ Department of Urology Union Hospital Tongji Medical College Huazhong University of Science and Technology Wuhan Hubei P. R. China; ^2^ Institute of Urology Union Hospital Tongji Medical College Huazhong University of Science and Technology Wuhan Hubei P. R. China; ^3^ Department of Pathogenic Biology School of Basic Medicine Huazhong University of Science and Technology Wuhan Hubei P. R. China

**Keywords:** hypoxia‐inducible factor 2alpha (HIF2α), NUDT1, oxidative stress, reactive oxygen species, sirtuin 3 (SIRT3)

## Abstract

**Background:**

The key role of hypoxia‐inducible factor 2alpha (HIF2α) in the process of renal cancer has been confirmed. In the field of tumour research, oxidative stress is also considered to be an important influencing factor. However, the relationship and biological benefits of oxidative stress and HIF2α in ccRCC remain unclear. This research attempts to explore the effect of oxidative stress on the cancer‐promoting effect of HIF2α in ccRCC and reveal its mechanism of action.

**Methods:**

The bioinformatics analysis for ccRCC is based on whole transcriptome sequencing and TCGA database. The detection of the expression level of related molecules is realised by western blot and PCR. The expression of Nucleoside diphosphate‐linked moiety X‐type motif 1 (NUDT1) was knocked down by lentiviral infection technology. The functional role of NUDT1 were further investigated by CCK8 assays, transwell assays and cell oxidative stress indicator detection. The exploration of related molecular mechanisms is realised by Luciferase assays and Chromatin immunoprecipitation (ChIP) assays.

**Results:**

Molecular screening based on knockdown HIF2α sequencing data and oxidative stress related data sets showed that NUDT1 is considered to be an important molecule for the interaction of HIF2α with oxidative stress. Subsequent experimental results showed that NUDT1 can cooperate with HIF2α to promote the progression of ccRCC. And this biological effect was found to be caused by the oxidative stress regulated by NUDT1. Mechanistically, HIF2α transcription activates the expression of NUDT1, thereby inhibiting oxidative stress and promoting the progression of ccRCC.

**Conclusions:**

This research clarified a novel mechanism by which HIF2α stabilises sirtuin 3 (SIRT3) through direct transcriptional activation of NUDT1, thereby inhibiting oxidative stress to promote the development of ccRCC. It provided the possibility for the selection of new therapeutic targets for ccRCC and the study of combination medication regimens.

## Graphical abstract

1. Silencing HIF2α in ccRCC can lead to a decrease in oxidative stress level.

2. The oxidative stress pathway inhibited by the high expression of NUDT1 plays an important role in the cancer‐promoting of HIF2α in ccRCC.

3. NUDT1 regulates ccRCC progression through cellular oxidative stress mediated by SIRT3 ubiquitination.

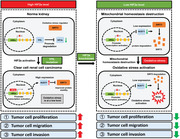

## 1 INTRODUCTION

Clear cell renal cell carcinoma (ccRCC) is the pathological subtype with the highest proportion of kidney cancer.^1^ In most ccRCCs, the hypoxia‐inducible factor (HIF) signal is widely activated due to the mutation of VHL.^2^ Hypoxia‐inducible factor (HIF) is a regulator of cell detection and adaptation to oxygen levels, specifically regulating oxygen homeostasis through transcriptional activation of downstream genes.^3^ Hypoxia‐inducible factor signals are usually mediated by two subunits (HIF1α and HIF2α), which mainly affect tumour progression through transcriptional regulation.^4^ Among them, HIF1α and HIF2α have been confirmed to have diametrically opposite effects in ccRCC, and HIF2α is considered to be one of the most significant oncogenes in ccRCC.^5^, ^6^, ^7^, ^8^, ^9^

Oxidative stress refers to the destruction of the redox balance in the cell.^10^, ^11^ It is mostly caused by the imbalance of mitochondrial function, which is directly mediated by reactive oxygen species (ROS) and can be directly regulated by mitochondrial related proteins such as NOX family and sirtuin 3 (SIRT3).^12^, ^13^ ROS is a double‐edged sword for cells. A certain range of ROS can clearly toxic substances maintain cell viability. However, when the level of ROS reaches an uncontrollable level, it can damage cell structure, affect cell metabolism and destroy nucleic acid stability, thereby causing cell death.^14^, ^15^, ^16^, ^17^ Previous studies have confirmed that there is also a great relationship between hypoxia‐induced signals and oxidative stress. Among them, HIF1α can affect the formation of many tumours through the mediation of ROS, and HIF2α affect the level of related mitochondrial matrix proteins through oxidative stress.^18^, ^19^, ^20^, ^21^ However, the relationship and biological benefits of oxidative stress and HIF2α in ccRCC have not yet been proven.

Nucleoside diphosphate linked moiety X‐type motif 1 (NUDT1) is an 18KD naked pyrophosphatase,^22^, ^23^ which is necessary for RAS/ROS‐related transformation and has important significance for the maintenance of cell viability.^24^, ^25^, ^26^, ^27^, ^28^, ^29^ In the field of oncology, NUDT1 has a certain research foundation, it mainly focuses on lung cancer,^30^ gastrointestinal tumours^31^, ^32^ and glioblastomas.^33^ However, its effects in ccRCC have not been elucidated.

In our study, a new mechanism has been clarified that HIF2α in ccRCC affects oxidative stress through transcriptional regulation of NUDT1, thereby affecting the progress of ccRCC.

## 2 MATERIALS AND METHODS

### 2.1 Human ccRCC tissues and cell lines

The HK‐2, A‐498, 786‐0, Caki‐1 and OSRC cell lines were from the American Type Culture Collection (ATCC, USA) and were cultivated under conditions recommended by the provider. DMEM (HyClone, UT, USA) were used to culture cell lines, with 1% penicillin‐streptomycin solution and 10% foetal bovine serum (Gibco, MA, USA) supplemented.

Human ccRCC tissue samples come from Wuhan Union Hospital. The pathological results of all samples were ccRCC. All the patients had not been treated by any antitumour treatment before surgery. Huazhong University of Science and Technology Committee approved this study. The tissue samples were acquired with informed consent signed by patients.

### 2.2 Immunohistochemistry

The tumour and adjacent normal tissues of ccRCC patients were treated with paraffin embedding, and use immunohistochemical staining to process the tissue sections. The tissue sections were processed in sequence according to the following steps: deparaffinisation, rehydration and incubating for antigen retrieval. NUDT1 antibody (ABclonal, A13330, Wuhan, China) or HIF2α antibody (ABclonal, A7553, Wuhan, China) were used as primary antibodies and incubated overnight. Immune complexes and nuclei were visualised by DAB and haematoxylinm, respectively (Biosharp, BS915, Hefei, China). The statistical analysis of all immunohistochemical staining is shown in Supplementary Information 4.

### 2.3 RNA extraction and qPCR

TRIzol reagent (Thermo, USA) was used to extract total RNA from tissues. Use 1 μg RNA for reverse transcription. Determination of mass and concentration of RNA solution by NanoDrop2000 spectrophotometer (NanoDrop Technologies, USA). GAPDH was used as an internal control. Use SYBR Green Mix (Thermo, USA) to perform qPCR according to the manufacturer's instructions. Real‐time qPCR was performed using the StepOnePlus™ PCR system (Applied Biosystems, California, USA). Analyse qRT‐PCR data with StepOne software 2.3 (Applied Biosystems, California, USA). 2^− ΔΔCT^ is used as the final statistical data. For ccRCC tissue samples, log _2_(2 ^− ΔCT^) is used as the final statistical data. Detailed primer sequences:

GAPDH: forward, 5′‐GAGTCCACTGGCGTCTTCA‐3′,

reverse, 5′‐GGTCATGAGTCCTTCCACGA‐3′;

NUDT1: forward, 5′‐ATCGTGTTTGAGTTCGTGGG‐3′,

reverse, 5′‐TGGAAACCAGTAGCTGTCGT‐3′;

and HIF2α: forward 5′‐ACAGGTGGAGCTAACAGGAC‐3′,

reverse, 5′‐CCGTGCACTTCATCCTCATG‐3′.

### 2.4 Whole transcriptome sequencing

HIF2α and NUDT1 stable knocked down ccRCC cell lines were established with lentivirus respectively. Every 5 × 10^6^ cells were vigorously pipetted with 1 ml of TRIzol until clear. Oebiotech, China (contact NO: OE2017H0149S) performed total RNA extraction and total transcriptome sequencing after stable inhibition of HIF2α.

Total RNA extraction, whole transcriptome sequencing and bioinformatics data analysis after NUDT1 stable knockdown were supported by Majorbio (China). The differentially expressed genes from the RNA sequencing results were analysed via Majorbio Cloud Platform.

### 2.5 Cell transfection and infection

The supplier of HIF2α‐targeted shRNAs and NUDT1‐targeted shRNAs is Genechem Co. Ltd (China). The supplier of the overexpression plasmids of NUDT1 is Genechem Co. Ltd (China). The HIF1α siRNA was purchased from GenePharma. The shRNA or expression vector of NUDT1 was infected into 786‐0 and A498 cells with the manufacturer's protocols, respectively. The vector backbone of shRNA is ‘hU6‐MCS‐CBh‐gcGFP‐IRES‐puromycin’. The vector backbone of LV‐NUDT1 is ‘Ubi‐MCS‐3FLAG‐SV40‐EGFP‐IRES‐puromycin’. The specific sequences of shRNA were:

shNUDT1‐1 Forward 5′‐ccCGACGACAGCTACTGGTTT‐3′

shNUDT1‐2 Forward 5′‐ccTGAGCTCATGGACGTGCAT‐3′

shHIF2α‐1 Forward 5′‐caGTACCCAGACGGATTTCAA‐3′

shHIF2α‐2 Forward 5′‐acTTCATGTCCATGCTGTGGC‐3′

### 2.6 Immunoprecipitation, Western blotting

After the cells were lysed with RIPA, centrifuged at 13 000 rpm for 5 min, the supernatant was collected in two parts: a small amount of lysate was taken as input and the remaining lysate was incubated with 2 μg of the corresponding antibody and 30 μl of agarose beads overnight at 4°C. Immune complexes were separated at 3000 rpm for 5 min. Carefully discard the supernatant, and wash the agarose beads 3 times with 200μl of lysis buffer. Finally, add 64 μl of RIPA lysis buffer and 16 μl of loading buffer, and boil for 10 min.

Western blotting: RIPA protein cleavage buffer (Beyotime, Wuhan, China) containing a mixture of protease inhibitors (Beyotime, P1005, Wuhan, China) and Phenylmethanesulfonyl fluoride (PMSF) (Beyotime, ST506, Wuhan, China) was used for protein extraction. The supplier of BCA kit is Beyotime Institute of Biotechnology (P0012S, China). Forty micrograms of protein was loaded in to SDS‐PAGE and then transferred to polyvinylidene fluoride (PVDF) membranes (Roche, 03010040001, Basel, Switzerland). Five percent non‐fat dried skim milk (BD Company, 232100, New Jersey, USA) was used to blocking. The membranes with primary antibodies were incubated overnight at 4°C. After washing with PBST 3 times, incubate with secondary antibody for 2 h at room temperature.

The primary antibody was diluted at a ratio of 1:1000. The primary antibodies used were shown as follows: NUDT1 (ABclonal Biotech Co., Ltd, A13330, Wuhan, China), HIF2α (ABclonal Biotech Co., Ltd, A7553, Wuhan, China), GAPDH (Proteintech, 60004‐1‐Ig, Chicago, USA), HO‐1 (ABclonal Biotech Co., Ltd, A11919, Wuhan, China), SOD2 (ABclonal Biotech Co., Ltd, A1340, Wuhan, China), CAT (ABclonal Biotech Co., Ltd, A5275, Wuhan, China), SIRT3 (ABclonal Biotech Co., Ltd, A7307, Wuhan, China), Ubiquitin (P4D1) Mouse mAb (CST, 3936S, Boston, USA) and OTUB1(ABclonal Biotech Co., Ltd, A11656, Wuhan, China). The secondary antibodies used for western blotting were as follows: HRP‐conjugated Affinipure Goat Anti‐Rabbit IgG(H+L) (Proteintech, SA00001‐2, USA), HRP‐conjugated Affinipure Goat Anti‐Mouse IgG(H+L) (Proteintech, SA00001‐1, Chicago, USA). The above secondary antibodies are diluted at a ratio of 1:2000. The statistical analysis of all western blots is shown in Supplementary Information 2.

### 2.7 Cell viability assays

Each 96‐well plate was inoculated with cells at a density of 2 × 10^3^/ well. Cell proliferation rate was measured with cell counting kit 8 (YEASEN Biotech Co.Ltd, 40203ES80, China) on the basis of the instruction manual. Add 110 μl CCK8 solution (10 μl CCK8:100 μl medium) to each well and incubate in the dark for 1 h. Determination of absorbance at 450 nm by NanoDrop 2000 spectrophotometer (NanoDrop Technologies, USA). Cell viability were measured at 0, 24, 48, 72 and 96 h after treatment.

### 2.8 Colony formation assays

A total of 10^3^ cells were seeded in 6‐well plates for 2 weeks and then fixed with methanol. Stain with 0.05% crystal violet (Servicebio, G1014, Wuhan, China) to visualise the colonies (>50 cells/colonies).

### 2.9 Wound healing assays

Ten microlitres pipette tips were used for wounding in a straight line when the cells have reached 70–80% fusion in the 6‐well plates. Afterwards, gently wash the cells with PBS and hold them at 37°C. Images were collected at 0, 12 and 24 h post wounding under UOP microscope (UOP Photoelectric Technology, DSZ2000, Chongqing, China) with UopView software (UOP Photoelectric Technology, Chongqing, China).

### 2.10 Transwell assays

Cells are cultured in serum‐free medium for 24 h before testing. With or without Matrigel™ (BD Company, BD‐354234, USA) transwell^®^ inserts (Corning Costar Corp, 01020023, USA) were used for migration and invasion assay. Cells are inoculated in the top compartment of the implant and allow cell invasion through the stroma. After 24 h of culture, fix the cells on the lower surface of the insert with methanol and stained with 0.05% crystal violet (Servicebio, G1014, Wuhan, China). Image acquisition and cell counting were performed in randomly selected areas.

### 2.11 Measurement of intracellular oxidative stress levels

Cells with a density of 5 × 10^5^–10^6^ cells/ml were measured for ROS levels through the Cellular ROS Detection Assay Kit (Abcam, ab186029, UK). Fluorescence microscope (Leica Microsystems, Leica DMI 3000 B, Wetzlar, Germany) was used to observe the fluorescence intensity and collect images.

The assay uses ROS deep red dye to quantify ROS: the dye is cell permeable and reacts with intracellular ROS to produce a deep red fluorescent signal (Ex/Em = 650/675 nm). The intensity of deep red fluorescence reflects the level of cellular ROS; the stronger the fluorescence is, the higher the cellular ROS level is.

### 2.12 Cellular MDA, protein carbonylation, 8‐oxodG measurement

Use malondialdehyde (MDA) assay kit (Nanjing Jiancheng, A003‐1‐2, China) to measure cell MDA. Intracellular malondialdehyde (MDA) expression was obtained at 532 nm.

The protein carbonylation level was completed by the Protein Carbonylation Content Assay kit (BOXBIO, AKAO007U, Beijing, China) according to the instructions.

8‐oxodG levels in cells and tissues were determined by Human 8‐oxodG ELISA Kit (Wuhan Hull Biotechnology, China) according to the instructions.

### 2.13 Cycloheximide (CHX) chase assay

Respectively treat the cells with 50 μM cycloheximide (CHX) for 0, 4, 8, 12 and 24 h. Cells were collected for protein extraction for western blot analysis. ImageJ software was used to quantitatively analyse protein levels.

Colchicine (S2284), chloroquine (S6999) and MG132 (S2619) were purchased from Selleck (Shanghai, China).

### 2.14 Sunitinib resistance assay

A498 and 786‐0 cells were suspended in DMEM (10% foetal bovine serum) with sunitinib or DMSO. Inoculate 5 × 10^3^ cells in each well of 96‐well plate. After 24 h, 96‐well dishes were cultivated at 37°C for 1, 2 and 3 h for measuring the cell validity. The concentrations of sunitinib were 1, 2, 4, 8, 16 and 32 μmol/ml, respectively

### 2.15 Chromatin immunoprecipitation (ChIP) assay

ChIP assay was performed with SimpleChIP^®^ Kit (Agarose Beads) (CST, 22188S, Boston, USA). The plasmids containing abridged NUDT1 promoter region (Figure S13) were constructed by Tianyi Huiyuan Biological Technology Co., Ltd (Wuhan, China). Rabbit IgG (CST, 2729, Boston, USA) was used to pretreat the cell lysate and Chip‐Grade protein G Agarose Beads (CST, 9007S, Boston, USA). Incubate an anti‐NUDT1 antibody (ABclonal, A13330, Wuhan, China) overnight at 4°C. Also use IgG as a negative control. The details of primers for amplifying the target sequence in the NUDT1 promoter are as below:

Control: Forward 5′‐CACCATTGCTAAACCACCCA‐3′

Reverse 5′‐AGGCTGAGTGGGCATGGG‐3′

Site 1: Forward 5′‐TGGCCAACATGATGAAACCC‐3′

Reverse 5′‐GGGTTCAGGCGATTCTCCT‐3′

Site 2: Forward 5′‐TCTCGAACTCCTGACCTCTG‐3′

Reverse 5′‐CAGCCTGGATGATAGCAAAACA‐3′

Site 3: Forward 5′‐CCGGTCTCTATGTCCATCTTTC‐3′

Reverse 5′‐GAGGGGAAGACAGCGACTC‐3′.

### 2.16 Luciferase assays

The construction of the truncated plasmids in the NUDT1 promoter regions is Tianyi Huiyuan Biological Technology Co., Ltd (Wuhan, China). Construction vector is pGL3‐Basic. The specific construction sequences were shown in Figure S13. Placed the cells in 24‐well plates and Lipofectamine 2000 was used to transfect complimentary DNA (Invitrogen, CA, USA). Use pRL‐TK as an internal control (Promega, E2241, USA). The luciferase activity was determined by double luciferase detection reagent (Promega, E1910, Madison, WI, USA), and it was performed according to the instructions.

### 2.17 Tumour formation assay

A total of 2 × 10^6^ A498 cells infected with shNUDT1‐1, shNUDT1‐2 or negative control shRNA were subcutaneously injected into 6‐week‐old nude mice (Vital River, Beijing, China) and 4 × 10^6^ A498 cells infected with negative control shRNA, shHIF2α, negative control shRNA+LvNUDT1 or shHIF2α+LvNUDT1 were injected into nude mice. The mice were euthanised 49 days after cell implantation and the tumour weight was measured. Measure tumour growth every week for 7 weeks with a digital caliper.

### 2.18 In vivo cancer metastasis assay

The metastatic ability of tumour cells was evaluated by the caudal vein metastasis model in nude mice. Note that, 1 × 10^6^ A498 cells infected with negative control shRNA, shHIF2α, negative control shRNA+LvNUDT1, shHIF2α+LvNUDT1, shNUDT1‐1 or shNUDT1‐2 were injected into the tail vein of mice. All mice were euthanized, and the liver tissues were fixed, paraffin‐embedded and sectioned after 7 weeks’ observation. And then perform H&E and IHC staining. Use UopView software (UOP Photoelectric Technology, Chongqing, China) to analyses the stained sections. The intensities of the staining were analysed using Image J.2.0 software (NIH, Maryland, USA).

### 2.19 TCGA database

The Cancer Genome Atlas (TCGA) is a cancer genomics project containing data on more than 20 000 primary cancers. TCGA mainly stores basic information of various types of tumours, including RNAseq, miRNAseq, DNA methylation, patient clinical information etc. It is a relatively one of the public available comprehensive databases so far. The TCGA Kidney Clear Cell Carcinoma (TCGA‐KIRC) database contains clinical data and related gene expression data of 533 patients with ccRCC. The gene mRNA data in the bioinformatics analysis was obtained from the TCGA database. We analysed relevant data with SPSS 22.0 and generated the curves with GraphPad Prism 7.0.

### 2.20 Bioinformatics analysis

Screening was performed using three independent sets of genes associated with oxidative stress pathways from the Oncomine Database (https://www.oncomine.org). The mRNA levels of genes in normal renal and ccRCC tumour tissue and clinical information of ccRCC patients comes from the TCGA datasets (http://www.cbioportal.org/public‐porta).

Use Gene Set Enrichment Analysis (GSEA) to determining whether a set of genes defined a priori show statistical differences between the two biological states. It can be used to study the gene function and metabolic pathways of a group of statistically significant genes. We used GSEA v4.1.0 for Windows (UC San Diego, San diego, USA) to determine the enrichment pathway of the RNAseq data of ccRCC in TCGA database.

### 2.21 Statistical analysis

Statistical analysis adopts *t*‐test or analysis of variance with SPSS 22.0. Among them, independent‐samples *t*‐test is applied to test whether the mean and variance of the samples of two independent normal populations are from the same population; paired‐samples *t*‐test is applied to test whether two related samples come from a normal population with the same mean. The Pearson's correlation coefficient calculated by linear correlation analysis is used to calculate the correlation between the two genes. Receiver operating characteristic (ROC) and area under curve (AUC) are measured to obtain the highest overall accuracy to compare the diagnostic abilities of different genes.

## 3 RESULTS

### 3.1 NUDT1 is closely related to HIF2α and oxidative stress and highly suggests the clinical prognosis of ccRCC

**FIGURE 1:**
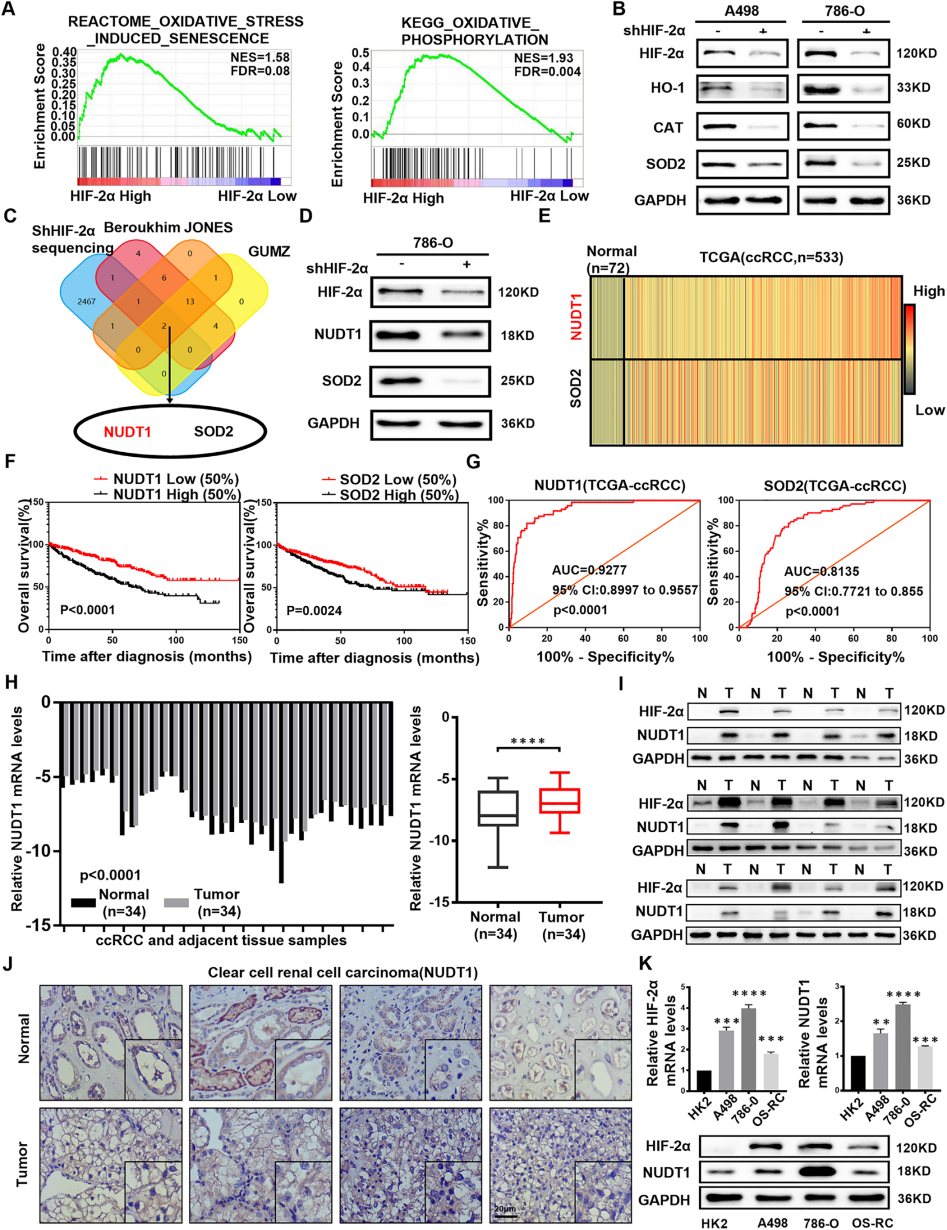
NUDT1 is closely related to HIF2α and oxidative stress and highly suggests the clinical prognosis of ccRCC. (A) GSEA correlation charts were screened according to the database from TCGA‐KIRC, and they reveal the correlation between oxidative stress and HIF2α mRNA levels in ccRCC. FDR < 25% and *p* ≤ .05 was considered statistically significant. (B) Based on 786‐O and A498 are ccRCC cell lines with VHL mutations, we selected them to more truly and comprehensively reflect the cellular biological functions and mechanisms under the action of HIF2α. Protein levels of oxidative stress‐related marker genes HO‐1, CAT and SOD2 in HIF2α knockout cell lines were shown by western blotting. (C) A Venn diagram composed of three independent oxidative stress pathway‐related gene sets from the Oncomine database (https://www.oncomine.org) and the entire transcriptome sequencing data obtained after stable HIF2α knockdown. The data were screened in ccRCC. (D) Protein levels of NUDT1and SOD2 in HIF2α knockdown 786‐0 cells were shown by western blotting. (E) The heatmap of NUDT1 and SOD2 mRNA levels in 533 ccRCC tissues and 72 matched tissues in TCGA database. (F) Kaplan–Meier curves of NUDT1 and SOD2 expression in ccRCC patients were used to measure the impact of overall survival (OS). The *p* value is obtained through log‐rank (Mantel‐Cox) test. (G) The ROC curve was drawn using the expression levels of NUDT1 and SOD2 in tumour samples and normal samples in the TCGA‐KIRC database to assess the sensitivity and specificity of its diagnostic ability. The ROC curves for NUDT1 (AUC = 0.9277 95% CI: 0.8997 to 0.9557; *p* < .0001) and SOD2 (AUC = 0.8135 95% CI: 0.7721 to 0.855; *p* < .0001) in ccRCC. (H) Histogram and box plot of NUDT1 mRNA expression in 34 pairs of ccRCC tissues and adjacent non‐malignant tissues; *t*‐test, *p* < .0001(paired‐samples *t*‐test for statistics). (I) Levels of the HIF2α and NUDT1 protein in ccRCC tissues and adjacent nonmalignant tissues (*n* = 12). (J) Immunohistochemical (IHC) staining for NUDT1 in ccRCC tissues and adjacent nonmalignant tissues. Scale bar: 20 μm. (K) NUDT1 mRNA levels in 3 ccRCC cell lines and normal cell lines. HIF2α and NUDT1protein levels in 3 ccRCC cell lines and normal cell lines; *t*‐test, *p < .05, ***p < .001 and ****p < .0001(independent‐samples *t*‐test for statistics)

Oxidative stress is an important pathophysiological process of cells, which is closely related to the development of a variety of tumours. HIF2α is a key cancer‐promoting gene of ccRCC. Through bioinformatics analysis, we found that HIF2α is highly related with related to ccRCC's oxidative stress (Figure 1A). However, the specific mechanism and mode of this correlation have not been reported in ccRCC. To clarify the relationship between oxidative stress and HIF2α in ccRCC, we used HIF2α‐specific shRNA to construct ccRCC cell lines with HIF2α stably knocked down (Figure S1A). As shown in Figure 1B, the levels of antioxidant enzymes are obviously reduced in the cell lines knocking down HIF2α, which means that HIF2α and oxidative stress have a significant negative regulation.^18^, ^34^ In order to make sure there is no compensation (specifically protein expression) mechanism between HIF1α and HIF2α, siRNA was transfected into ccRCC cell lines to knock down HIF1α. Western blot results showed that the change of HIF1α protein expression had no effect on the expression of HO‐1, CAT and SOD2 (Figure S1B). However, the mRNA levels of HO‐1 and SOD2 are positively correlated with HIF2α (Figure S1C, D). In order to find the potential mechanism of HIF2α regulating oxidative stress, we used the sequencing data after knocking down HIF2α and the ccRCC‐related oxidative stress data set from the Oncomine database for molecular screening. The results showed that there are two molecules, including NUDT1 and SOD2, that are significantly differentially expressed in ccRCC (Figure 1C). Then, the expression trend of NUDT1 and SOD2 was verified by the 786‐0 cells with HIF2α stably knocked down (Figure 1D). Further bioinformatics analysis based on TCGA showed that the average line of NUDT1 and SOD2 in ccRCC showed a high expression trend (Figures 1E and S1E). Subsequently, the construction of the Kaplan–Meier curve also indicated that these two molecules are negatively related to patient survival; that is higher expression levels have a shorter survival time, and more importantly, NUDT1 has a more significant trend (Figure 1F). Moreover, through ROC curve analysis, the AUC of NUDT1 is higher than SOD2, indicating that NUDT1 has better diagnostic value in ccRCC (Figure 1G). Therefore, NUDT1 was selected as the target molecule for in‐depth research.

We conducted further bioinformatics analysis to verify the above results. Similarly, the expression trend of NUDT1 in ccRCC was once again proved by data from the Oncomine database (Figure S1F). Furthermore, bioinformatics analysis was performed using the data of 519 groups of ccRCC patients with complete clinical parameters in TCGA‐KIRC. As shown in Table 1, the expression level of NUDT1 will gradually increase with the increase of ccRCC staging and grading (Figure S1G). Subsequently, the subgroup survival analysis based on ccRCC clinical indicators once again clarified the important guiding significance of NUDT1 on the survival time of ccRCC patients (Figures S2 and S3). Then, we used the level of HIF2α expression as the screening condition to draw the OS Kaplan–Meier curves of the high and low expression of NUDT1 in different clinical parameters. The results indicated that the high expression of HIF2α and NUDT1 obviously reduces the clinical prognosis (Figure S4). Moreover, the COX survival regression analysis based on the TCGA database clarified the status of NUDT1 as an independent risk factor for ccRCC (Table 2). Although there are differences between different normal and tumour samples, which may reflect the heterogeneity of patient tissues, the expression of NUDT1 in tumours is consistent with HIF2α and both are upregulated compared to normal tissues adjacent to cancer (Figures 1H–J and S5). Consistent with the results of tissue verification, experiments based on multiple cell lines showed that the expression levels of HIF2α and NUDT1 in ccRCC cells were obviously increased (Figure 1K). In summary, we conclude that NUDT1 is closely related to HIF2α and oxidative stress and highly suggests the clinical prognosis of ccRCC.

**TABLE 1:** Correlation between NUDT1 mRNA expression and clinicopathological parameters of ccRCC patients

Parameter	Total [cases, (%)]	NUDT1 mRNA expression [cases, (%)]		*p* Value
		Low (*n* = 259)	High (*n* = 260)	
Age (years)				.380
≤60	258 (49.7)	134 (51.7)	124 (47.7)	
>60	261 (50.2)	125 (48.3)	136 (52.3)	
Gender				.001*
female	182 (35.1)	109 (42.1)	73 (28.1)	
male	337 (64.9)	150 (57.9)	187 (71.9)	
T stage				.000*
T1+T2	332 (64.0)	196 (75.7)	136 (52.3)	
T3+T4	187 (36.0)	63 (24.3)	124 (47.7)	
N stage				.006*
N0+ NX	505 (97.3)	257 (99.2)	248 (95.4)	
N1	14 (2.7)	2 (0.8)	12 (4.6)	
M stage				.000*
M0+ MX	441 (85.0)	237 (91.5)	204 (78.5)	
M1	78 (15.0)	22 (8.5)	56 (21.5)	
G stage				.000*
G1+G2	239 (46.1)	156 (60.2)	83 (31.9)	
G3+G4	280 (53.9)	103 (39.8)	177 (68.1)	
TNM stage				.000*
I+II	314 (60.5)	192 (74.1)	122 (46.9)	
III+IV	205 (39.5)	67 (25.9)	138 (53.1)	

* Indicates that the *p* value is statistically significant, *p* < .05.

Relevant clinical data of ccRCC patients are all from the TCGA‐KIRC database.

**TABLE 2:** Univariate and multivariate analyses of NUDT1 mRNA level and patient overall survival or disease‐free survival

Variable	Overall survival				Disease‐free survival			
	Univariate analysis		Multivariate analysis^c^		Univariate analysis		Multivariate analysis^c^	
	HR^a^ (95% CI^b^)	*p* Value	HR^a^ (95% CI^b^)	*p* Value	HR^a^ (95% CI^b^)	*p* Value	HR^a^ (95% CI^b^)	*p* Value
Age (≤60 years vs. >60 years)	1.742 (1.281–2.367)	<.001*	1.649 (1.211–2.246)	.002	1.431 (0.912–2.246)	.119	–	–
Gender (female vs. male)	0.956 (0.701–1.303)	.774	–	–	1.192 (0.731–1.946)	.477	–	–
T stage (T1 or T2 vs. T3 or T4)	3.038 (2.243–4.114)	<.001*	–	–	6.645 (4.016–10.995)	<.001*	–	–
N stage (N0 or NX vs. N1)	3.554 (1.872–6.747)	< .001*	–	–	7.203 (3.429–15.130)	<.001*	2.921 (1.378–6.191)	.005*
M stage (M0 or MX vs. M1)	4.290 (3.145–5.854)	<.001*	2.414 (1.668–3.495)	<.001*	11.989 (7.595–18.923)	<.001	4.662 (2.802–7.758)	<.001*
G grade (G1 or G2 vs. G3 or G4)	2.607 (1.855–3.662)	<.001*	1.546 (1.073–2.226)	.019*	5.227 (2.876–9.498)	<.001	2.966 (1.605–5.481)	<.001*
TNM stage (Stage I+II vs. Stage III+IV)	3.716 (2.709–5.097)	<.001*	1.927 (1.303–2.850)	<.001*	11.115 (6.112–20.215)	<.001	4.244 (2.142–8.409)	<.001*

^a^ Hazard ratio, estimated from Cox proportional hazard regression model.

^b^ Confidence interval of the estimated HR.

^c^ Multivariate models were adjusted for T, N, M classification, age and gender.

* Indicates that the *p* value is statistically significant, *p* < .05.

Relevant clinical data of ccRCC patients are all from the TCGA‐KIRC database.

### 3.2 NUDT1 promotes the progress of ccRCC

**FIGURE 2:**
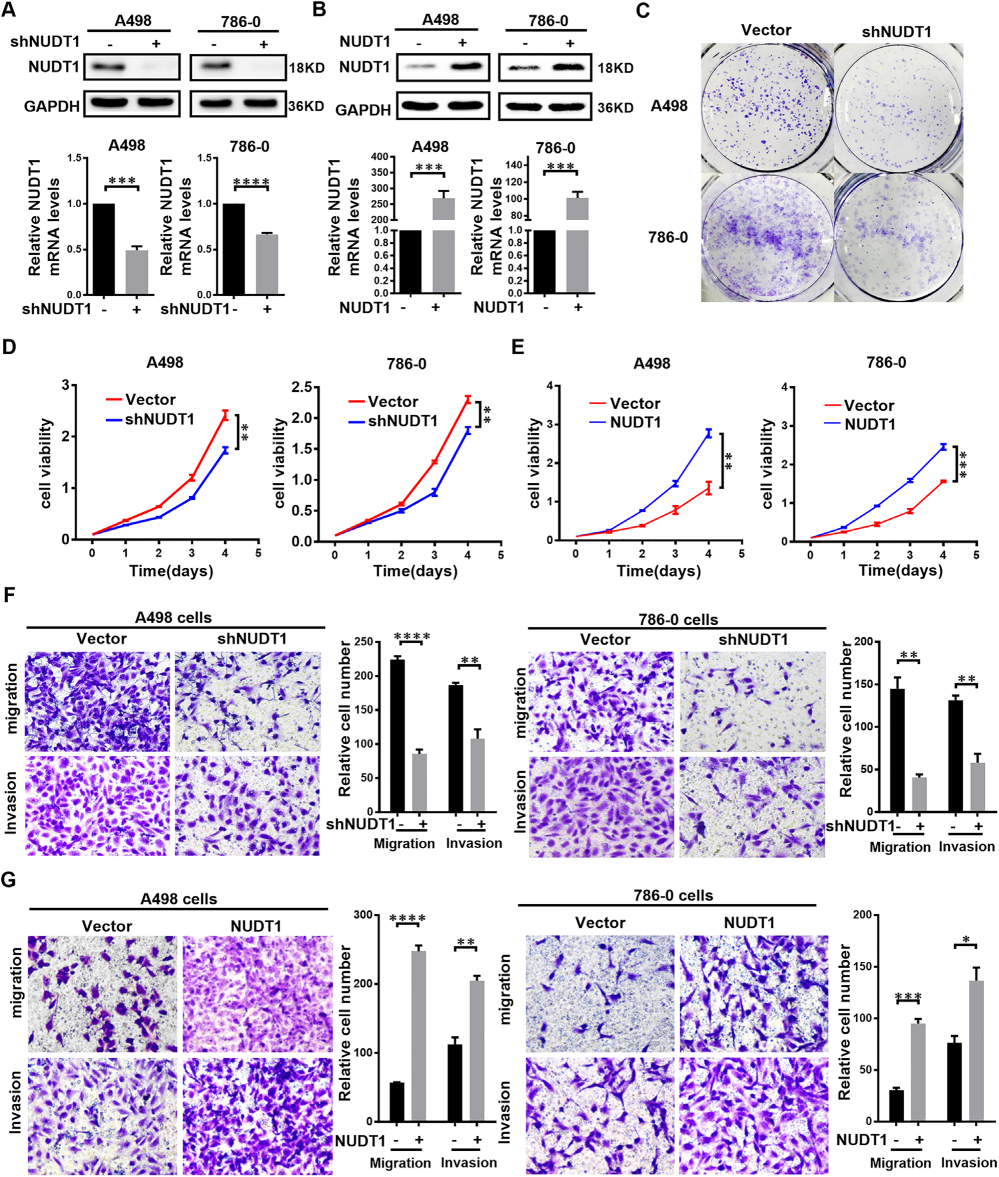
NUDT1 promotes the progress of ccRCC. NUDT1 knockdown or overexpressing ccRCC cell lines were constructed by transfecting lentivirus, respectively. The results are expressed as the mean ± SEM of three independent experiments, and there are at least three replicates in each independent experiment. *t*‐test, ****p < .0001, ***p < .001, **p < .01 and *p < .05 (independent‐samples *t*‐test for statistics). (A) Western blotting and qPCR were used to verify NUDT1 knockdown at the protein and mRNA levels, respectively. (B) Western blotting and qPCR were used to verify NUDT1 overexpression at the protein and mRNA levels, respectively. (C) Colony formation experiment results are shown for NUDT1 knockdown cells. (D) CCK8 assays were used to determine the cell growth of NUDT1 knockdown cell lines. (E) CCK8 was used assays to determine the cell growth curve of NUDT1‐overexpressing cell lines. (F) The results of the transwell assay of the migration and invasion of NUDT1 knockdown cell lines. (G) The results of the transwell assay of the migration and invasion of NUDT1‐overexpressing cell lines

The above studies have confirmed the characteristics of NUDT1 as a biomarker of ccRCC, so the specific biological function of NUDT1 in ccRCC is the focus of this part of the investigation. In order to analyse the biological functions of NUDT1, we used NUDT1 specific shRNA and overexpression lentivirus to construct ccRCC cell lines model with NUDT1 stably knocked down and overexpressed NUDT1 (Figure 2A, B). Analysis experiments based on the growth rate of tumour cells suggest that knocking down NUDT1 can significantly inhibit the proliferation rate of ccRCC cell lines (Figure 2C, D), while overexpression of NUDT1 can have the opposite result (Figure 2E). Similarly, analysis based on the migration and invasion capabilities of ccRCC cells also suggests that knocking down NUDT1 can significantly inhibit the migration and invasion of ccRCC cells (Figures 2F and S6), while overexpression of NUDT1 can significantly promote the above capabilities (Figure 2G). Then, NUDT1 overexpression lentivirus was infected into the ccRCC cell lines with stable knockdown of NUDT1 to construct functional recovery cell lines (Figure S7A). The results of functional recovery experiments showed that overexpression of NUDT1 can reverse the inhibition of downregulation of NUDT1 on cell proliferation, migration and invasion (Figure S7B–E). These findings imply that NUDT1 is an important cancer‐promoting gene in ccRCC, which can significantly promote the progress of ccRCC.

### 3.3 NUDT1 reduces the biological effects of oxidative stress

**FIGURE 3:**
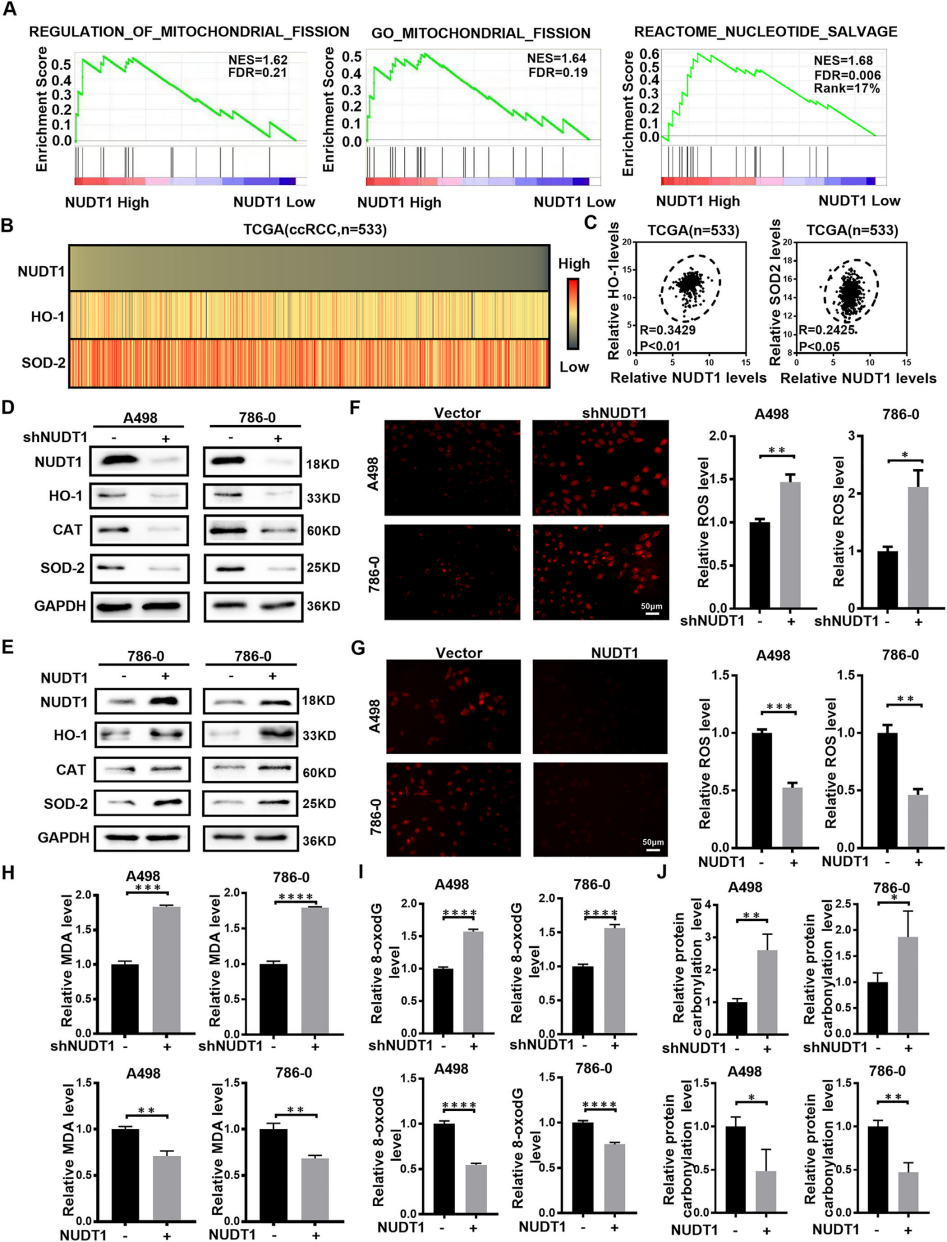
NUDT1 reduces the biological effects of oxidative stress. (A) The GSEA correlation charts were screened according to the TCGA‐KIRC database. FDR < 25% and *p* < .05 was considered statistically significant. (B), (C) The correlation heatmap and the linear correlation curve (*R* stands for Pearson's correlation coefficient) between NUDT1 and the most critical molecules related to oxidative stress (HO‐1 and SOD2) based on the data from the TCGA‐KIRC database. (D), (E) Protein levels of ROS and oxidative stress‐related marker genes (HO‐1, CAT and SOD2) in NUDT1 knockdown and overexpressing cell lines were detected by western blotting. (F) Images of NUDT1 knockdown and control cell lines stained with a ROS deep red stain solution in a 96‐well plate. Compared with control cells, the deep red fluorescence of ccRCC cells knocked down NUDT1 was enhanced. Scale bar: 50 μm. (G) Images of NUDT1 overexpressing and control cell lines stained with a ROS deep red stain solution in a 96‐well plate. Compared with control cells, the deep red fluorescence in ccRCC cells overexpressing NUDT1 was reduced. Scale bar: 50 μm. (H) MDA content in NUDT1 knockdown and overexpressing cell lines. Experiments were repeated 3 times, as described in Section 2; *t*‐test, ****p < .0001, ***p < .001, **p < .01 and *p < .05 (independent‐samples *t*‐test for statistics). (I) Relative 8‐oxodG levels in NUDT1 knockdown and overexpressing cell lines; *t*‐test, ****p < .0001, ***p < .001, **p < .01 and *p < .05 (independent‐samples *t*‐test for statistics). (J) Relative protein carbonylation levels in NUDT1 knockdown and overexpressing cell lines; *t*‐test, ****p < .0001, ***p < .001, **p < .01 and *p < .05 (independent‐samples *t*‐test for statistics)

CcRCC is a special tumour type that exhibits a significant change in cellular redox balance.^35^, ^36^, ^37^ Encouraged by the above results, more in‐depth research based on NUDT1 was carried out. It is worth noting that the screening of NUDT1 is based on oxidative stress, and at the same time, oxidative stress plays an essential role in tumour progression. Therefore, the specific regulation between NUDT1 and oxidative stress in ccRCC has become the focus of this unit. We first performed bioinformatics analysis to clarify the association between NUDT1 and oxidative stress. GSEA results suggest that NUDT1 is involved in mitochondrial formation, division and nucleotide salvage, which all imply that NUDT1 is closely related to oxidative stress (Figure 3A). In order to verify the above conjecture, we constructed a correlation heat map and a correlation curve between NUDT1 and common antioxidant enzymes (Figure 3B, C). At the same time, we tested the expression of the corresponding antioxidant enzymes in cells stably knocked out and overexpressing NUDT1. The results revealed that NUDT1 is highly positively correlated with these antioxidant enzymes. Knockdown of NUDT1 obviously reduces corresponding antioxidant enzymes’ expression, while overexpression of NUDT1 has the opposite effect (Figure 3D, E). MDA detection and ROS fluorescence detection are used to more intuitively display the level of cellular oxidative stress. The results are very similar, that is, knockdown of NUDT1 activates oxidative stress, while overexpression of NUDT1 inhibits oxidative stress in ccRCC cells (Figure 3F–H). 8‐oxo‐2′‐deoxyguanosine (8‐oxodG), as the main oxidation product of ROS‐induced guanosine (dG), is often used as an indicator of DNA oxidative damage.^38^, ^39^ Studies have shown that increased oxidative stress can cause protein carbonylation.^40^, ^41^ Therefore, we detected 8‐oxodG and protein carbonylation levels in ccRCC cells that stably knockdown and overexpressing NUDT1. The results showed that knocking out NUDT1 was accompanied by increased levels of 8‐oxodG and protein carbonylation levels, while overexpression of NUDT1 was accompanied by decreased levels of 8‐oxodG and protein carbonylation levels (Figure 3I, J). The above results indicate that NUDT1 reduces the biological effects of oxidative stress.

### 3.4 NUDT1 regulates the progress of ccRCC by inhibiting the ubiquitination of SIRT3 to affect cellular oxidative stress

**FIGURE 4:**
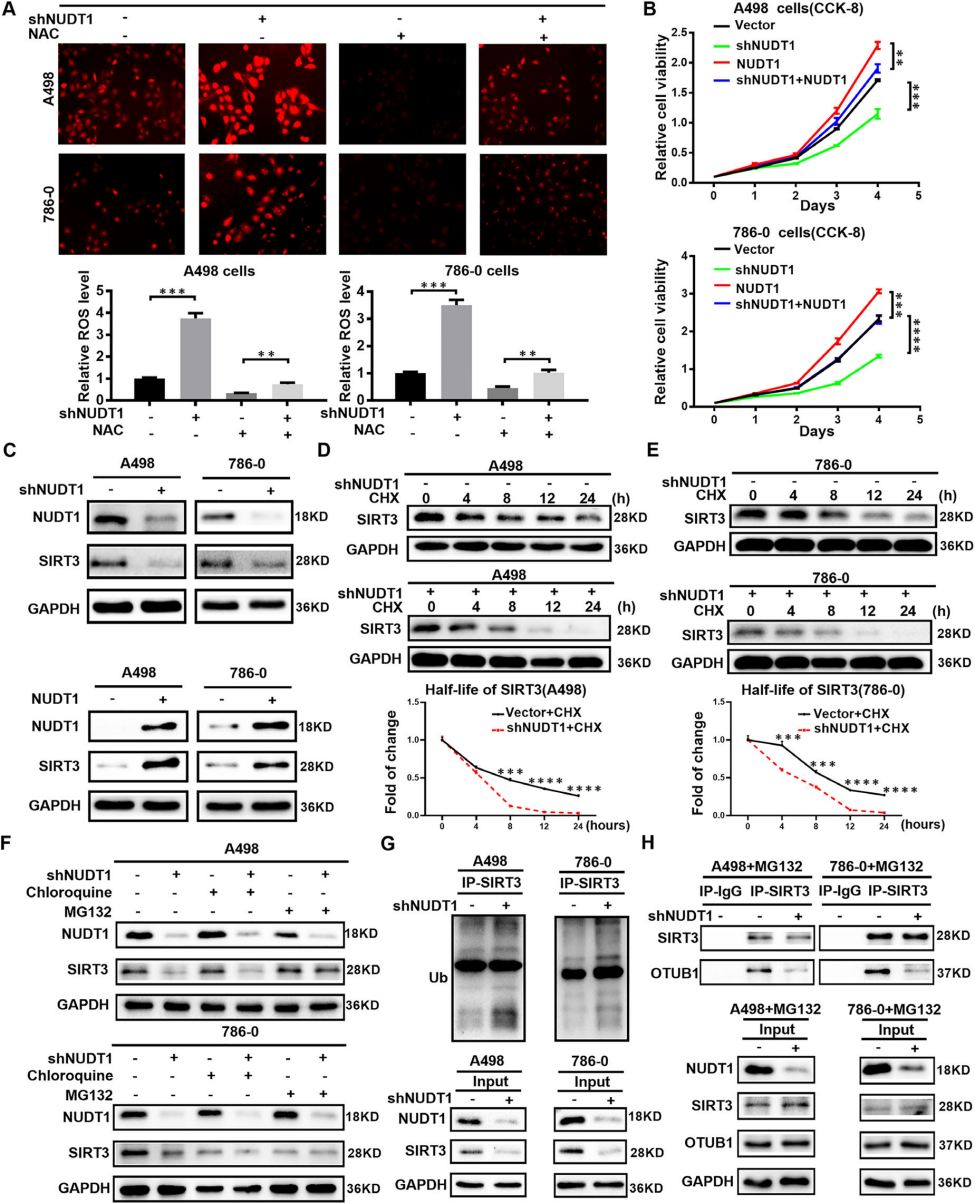
NUDT1 regulates the progress of ccRCC by inhibiting the ubiquitination of SIRT3 to affect cellular oxidative stress. Acetylcysteine (NAC) was used as a cellular ROS inhibitor to construct a functional recovery model of the ccRCC cell line stably knocked out of NUDT1. (A) ROS fluorescence staining image and the corresponding quantitative analysis results of the recovered cell line. (B) CCK8 proliferation curve of functional recovery cell line. (C) Protein levels of SIRT3 in NUDT1 knockdown and overexpressing cells were shown by western blotting. (D) Protein stability experiment of A498 cells knocked down NUDT1. Cells were treated with 50 μM cycloheximide (CHX) and harvested at specific time points, 0 h, 4 h, 8 h, 12 h, 24 h. (E) Protein stability experiment of 786‐0 cells knocked down NUDT1. (F) A498 and 786‐0 cells were treated independently with 20 μM MG132 and chloroquine for 12 h, and the expression of SIRT3 protein was analysed by Western blot. (G) A498 and 786‐0 cells infected with negative control and shNUDT1 virus were immunoprecipitated with SIRT3 antibody, and Western blotting with ubiquitin (Ub) antibody. (H) A498 and 786‐0 cells were treated with 20 μM MG132. A498 and 786‐0 cells infected with negative control and shNUDT1 virus were immunoprecipitated with SIRT3 antibody, and the expression changes of OTUB1 bound to SIRT3 were determined by western blotting

The above studies have confirmed that NUDT1 has a significant negative regulation of cellular oxidative stress. At the same time, cellular oxidative stress has been confirmed to play a vital role in tumour progression. Therefore, it is reasonable to believe that cellular oxidative stress also plays an important role in NUDT1's regulation of the progress of ccRCC. To verify the above hypothesis, we used acetylcysteine (NAC), an inhibitor of cellular ROS, to construct functional recovery models in ccRCC cells with stably NUDT1 knocked down (Figure 4A). As shown in the figure, after the use of Acetylcysteine to reduce cell ROS, the inhibition of cell proliferation caused by NUDT1 knockdown can be significantly reversed (Figure 4B). At the same time, similar results can be obtained from experiments on migration and invasion ability. Inhibition of cellular ROS can also significantly reverse the inhibition of NUDT1 on cell migration and invasion (Figure S8A, B). Based on the above results, we can conclude that cellular oxidative stress plays a vital role in the biological functions mediated by NUDT1 in ccRCC.

Encouraged by the above results, the next focus is to explore the specific mechanism by which NUDT1 regulates cellular oxidative stress. As known, SIRT3 is an important cell oxidative stress regulator; especially it participates in the regulation of ROS in many tumour fields.^42^, ^43^, ^44^ It is worth noting that through transcriptome sequencing and bioinformatics analysis (Figure S9), we found that NUDT1 is closely related to SIRT3. Considering the molecular biological functions of these two molecules, we have reason to believe that SIRT3 is very likely to be an important downstream of NUDT1 in regulating oxidative stress. Experiments have confirmed that NUDT1 has a significant positive regulation of SIRT3 in ccRCC; that is, NUDT1 overexpression can significantly enhance the SIRT3 expression, while knocking down NUDT1 has the opposite effect (Figure 4C). In order to eliminate the effect of proliferation on SIRT3 levels, we used colchicine to treat ccRCC cells that knock down or overexpress NUDT1, respectively. The experimental results are consistent with the above results (Figure S8C). The protein level of the mitochondrial marker COXIV^45^ was determined to exclude the influence of the number of mitochondria on the level of SIRT3 (Figure S8D). The results showed that mitochondrial markers increased slightly after knocking down NUDT1. A series of results show that NUDT1 can regulate the protein level of SIRT3, but not caused by the proliferation level or mitochondrial level changes caused by NUDT1. A large number of studies have confirmed that the stability regulation of SIRT3 plays an important role in the regulation of oxidative stress in tumour. Moreover, the functional analysis of NUDT1 has shown that it is highly related to ubiquitination, protein synthesis and protein hydromechanical pathways, which are closely related to the regulation of protein stability. Accordingly, we introduced that NUDT1's regulation of SIRT3 is based on the regulation of protein stability. In order to verify the above conjecture, we used cycloheximide (CHX) to construct protein half‐life experiments. As shown in the figure, knocking down NUDT1 can significantly accelerate the degradation rate of SIRT3 (Figure 4D, E). It can be seen that NUDT1 can significantly improve the protein stability of SIRT3. Protein degradation is mainly mediated through the lysosomal‐dependent pathway or the ubiquitin‐proteasome pathway.^46^ In order to determine the specific way that NUDT1 affects the stability of SIRT3, lysosomal inhibitor chloroquine and proteasome inhibitor MG132 were added to the ccRCC cell lines with stable knockdown of NUDT1, and the expression of SIRT3 was detected by Western blotting. After treatment with the chloroquine, the expression of SIRT3 in knockdown NUDT1 cells was significantly lower than that of control cells, but after treatment with the MG132, the expression of SIRT3 in knockdown NUDT1 cells was the same as that of control cells (Figure 4F). The results showed that NUDT1 regulates the expression of SIRT3 mainly through the ubiquitin‐proteasome pathway. Later, Western blot results showed that knockdown of NUDT1 increased the ubiquitination level of SIRT3 (Figure 4G). Based on the above research results, the focus of our next exploration is how NUDT1 affects SIRT3 ubiquitination. As we all know, the ubiquitination process is jointly regulated by ubiquitination‐related enzymes and deubiquitinating enzymes. As a deubiquitinating enzyme, OTUB1 not only has the classic deubiquitinating enzyme activity, but also shows a non‐classical activity that does not depend on catalysis, which can inhibit the ubiquitination of a variety of proteins.^47^ Treating ccRCC cells with MG132 to inhibit SIRT3 ubiquitination, we found that knocking down NUDT1 can significantly reduce the expression of OTUB1 that binds to SIRT3, which indicates that OTUB1 plays an important role in the process of NUDT1 affecting SIRT3 ubiquitination (Figure 4H). In summary, the conclusion can be drawn that NUDT1 regulates the progress of ccRCC by inhibiting the ubiquitination of SIRT3 to affect cellular oxidative stress.

### 3.5 The oxidative stress pathway inhibited by the highly expressed NUDT1 is a key link in the process of HIF2α promoting ccRCC

**FIGURE 5:**
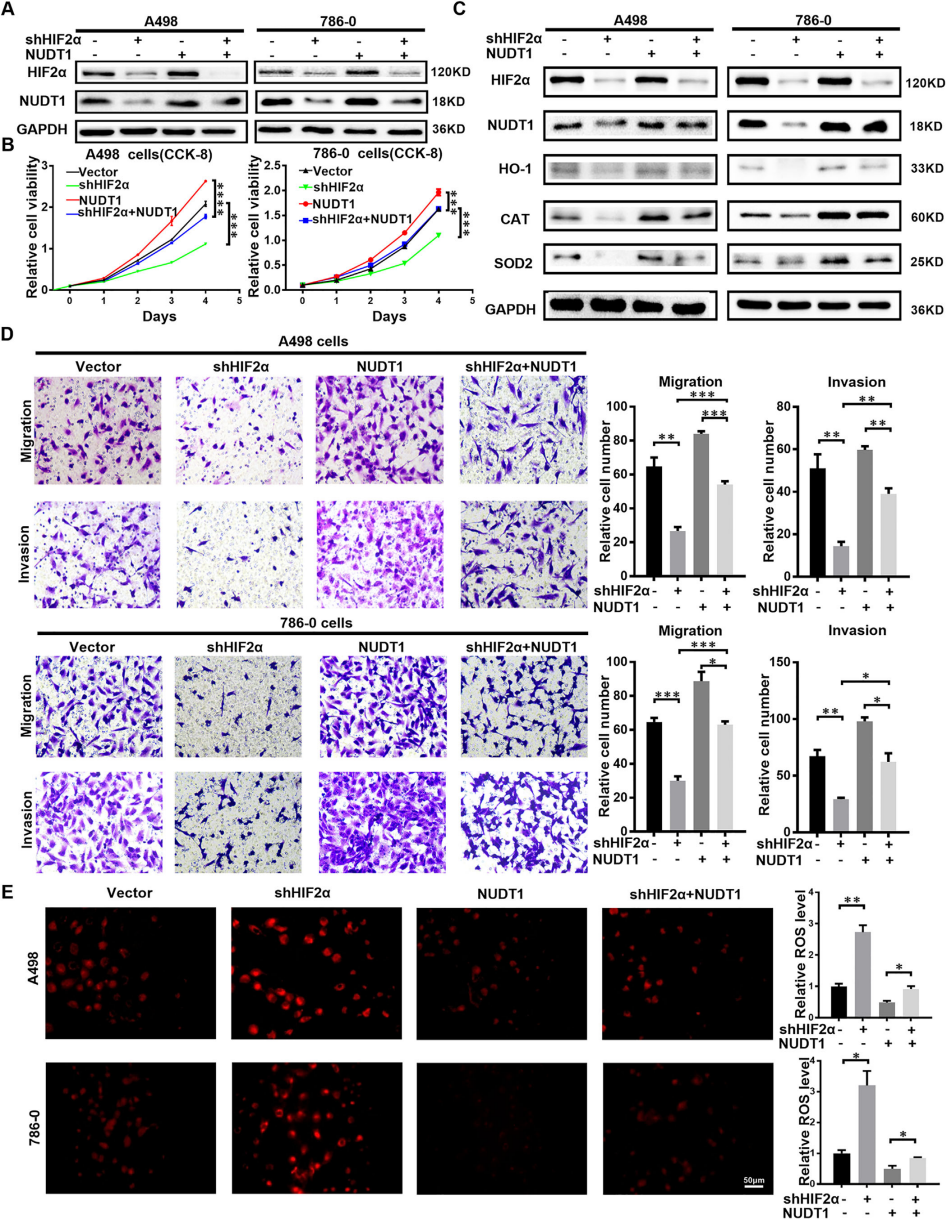
The oxidative stress pathway inhibited by the highly expressed NUDT1 is a key link in the process of HIF2α promoting ccRCC. We performed double infection in A498 and 786‐O cells with sh‐HIF2α and NUDT1 overexpression lentivirus to cause HIF2α knockdown and NUDT1 overexpression. (A) HIF‐2α and NUDT1 protein levels in transfected cell lines are shown by western blotting. (B) Cell growth curves based on CCK8 assays are shown for transfected cell lines; *t*‐test, ****p < .0001, ***p < .001, **p < .01 and * *p* < .05 (independent‐samples *t*‐test for statistics). (C) Western bolt showing levels of the HIF2α, NUDT1, HO‐1, CAT, SOD2 in the indicated cell lines. (D) The results of the transwell assay of the migration and invasion of transfected cell lines; *t*‐test, ****p < .0001, ***p < .001, **p < .01 and *p < .05 (independent‐samples *t*‐test for statistics). (E) Images and quantitative analysis results of NUDT1 transfected cell lines stained with ROS deep red stain solution in a 96‐well plate. Scale bar: 50 μm

Considering the reasons of NUDT1 based on HIF2α knockdown and oxidative stress screening, as well as the important characteristics of NUDT1 as a cancer‐promoting gene, we have reason to believe that NUDT1 has a potentially key role in the cancer‐promoting pathway of HIF2α. We used NUDT1 overexpression lentivirus to construct functional recovery models in ccRCC cells with HIF2α stably knocked down to verify the above hypothesis (Figure 5A). As shown in the Figure 5B, after the use of lentivirus to overexpress NUDT1, the inhibition of cell proliferation caused by HIF2α knockdown can be significantly reversed. At the same time, similar results can be obtained from experiments on cell migration and invasion ability. The NUDT1 overexpression can also significantly reverse the negative effects of HIF2α knockout on cell migration and invasion (Figure 5D). When talking about oxidative stress, similar results can also be observed; that is, overexpression of NUDT1 is able to reverse the ROS production caused by knockdown of HIF2α (Figure 5C, E). Consistently, overexpression of NUDT1 can reverse DNA oxidative damage caused by HIF2α knockdown (Figure S10). In all, we can conclude that the oxidative stress pathway inhibited by the highly expressed NUDT1 is a key link in the procession of HIF2α promoting ccRCC.

### 3.6 HIF2α directly transcriptionally regulates the expression of NUDT1 in ccRCC

**FIGURE 6:**
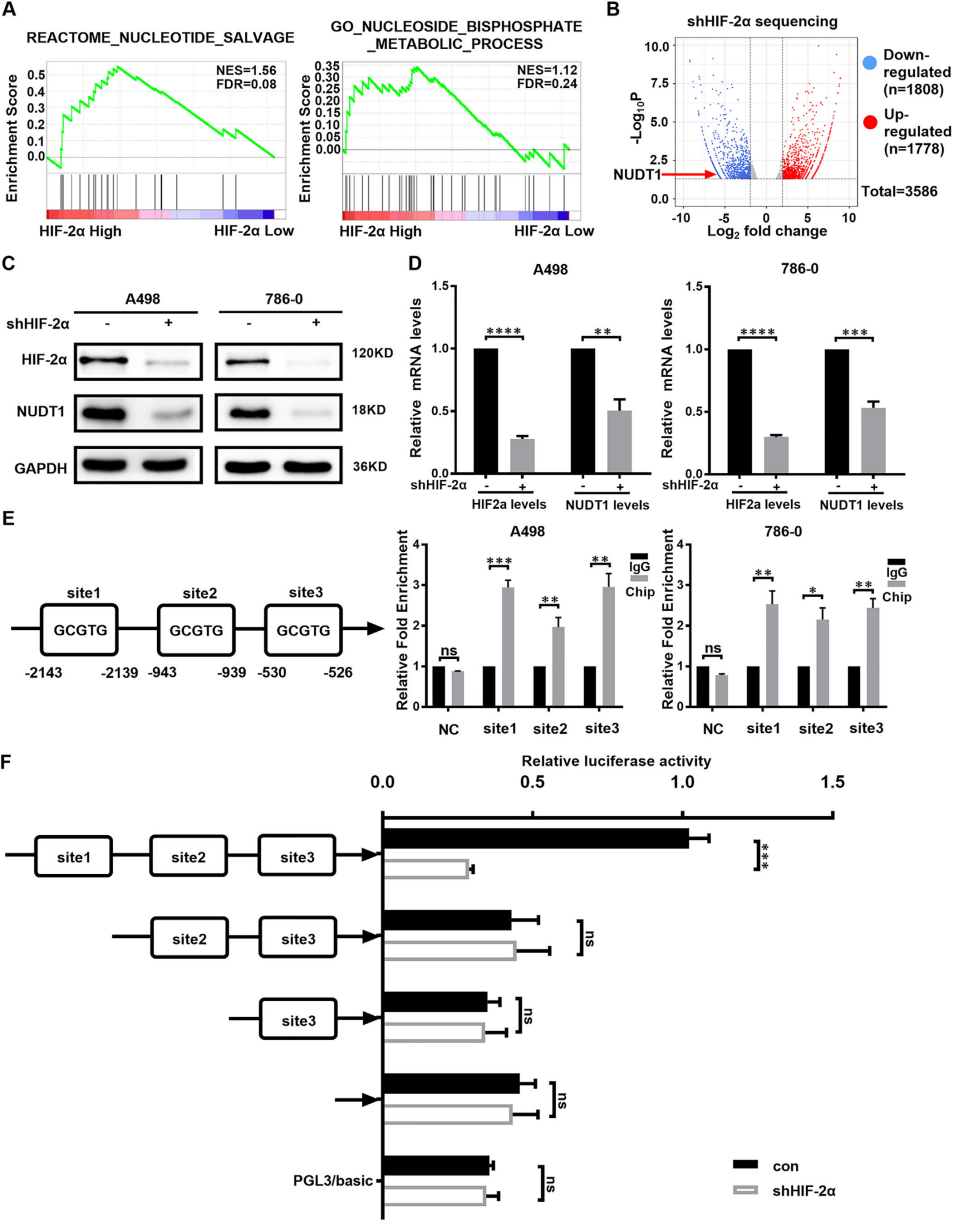
HIF2α directly transcriptionally regulates the expression of NUDT1 in ccRCC. (A) GSEA correlation charts were screened according to the database from TCGA‐KIRC, reflecting the correlation between nucleoside metabolism and HIF‐2a mRNA levels in ccRCC. FDR < 25% and *p* < .05 was considered statistically significant. (B) Volcano map of whole transcriptome sequencing data after HIF2a knockdown. After HIF2a knockdown, there were 3586 meaningful differentially expressed genes, of which 1778 were upregulated genes and 1808 were downregulated genes (including NUDT1). (C), (D) The protein and mRNA levels of NUDT1 after knocking down HIF2α are shown by western blotting and qPCR; *t*‐test, ****p < .0001, ***p < .001, **p < .01 and *p < .05 (independent‐samples *t*‐test for statistics). (E) ChIP experiment results of potential HIF2α binding sites in the NUDT1 promoter are based on the HIF2α binding sequence; *t*‐test, ****p < .0001, ***p < .001, **p < .01 and *p < .05 (independent‐samples *t*‐test for statistics). (F) The results of the luciferase assay were obtained according to Section 2 described previously. The truncation of the promoter showed that HIF2α bound to the NUDT1 promoter 1 region (–2143 to –2139), which is important for HIF2α to regulate NUDT1; *t*‐test, ****p < .0001, ***p < .001, **p < .01 and *p < .05 (independent‐samples *t*‐test for statistics)

Above research results indicate that NUDT1 may be an important potential downstream of HIF2α in promoting cancer in ccRCC, so the specific regulatory mechanism between them has become the focus of this unit. First, through further bioinformatics analysis, we once again clarified the correlation between NUDT1 and HIF2α (Figure 6A). Subsequently, sequencing (Figure 6B) and related expression experiments based on knockdown of HIF2α all indicate that HIF2α positively regulates NUDT1; that is, knockdown of HIF2α can significantly reduce the protein and RNA levels of NUDT1 in ccRCC (Figures 6C, D and S11A, B). In order to further increase the reliability of the conclusion, the cell hypoxia experiment was carried out. A498 and 786‐0 parental cells were cultured in 1% O_2_ to induce increased HIF2α levels. Compared with cells cultured under normoxia, the expression of HIF2α increased under hypoxic conditions, and NUDT1 increased accordingly (Figure S11C). At the same time, the protein levels of HIF2α, HO‐1, CAT and SOD2 in ccRCC cell lines that were knocked down and overexpressed in NUDT1 were detected by western blot (Figure S11D, E). The results showed that NUDT1 affects cell oxidative stress without affecting the level of HIF2α protein. It was further verified that NUDT1 is a downstream gene of HIF2α affecting cell oxidative stress. Based on the characteristics of HIF2α as a transcription factor and the fact that HIF2α and NUDT1 are positively regulated, the first thing we consider in terms of mechanism is direct transcription regulation. According to the prediction of the sequence information of the hypoxia‐inducing unit, there are three potential binding sites for HIF2α in the 3000 bp promoter region upstream of the transcription start site of NUDT1. We named the site as 1 to 3 based on the location of these sites (Figure S12). In order to verify the corresponding specific mechanism, we successively carried out Chip experiments and carried out luciferase assay by constructing truncated plasmids (Figure S13). The results showed that in terms of binding, HIF2α can bind to the site 1–3 in the promoter region of NUDT1 (Figure 6E), while in terms of function, HIF2α only has a significant effect on site1 (Figure 6F). In Figure 6F, the decreasing on luciferase activity mediated by HIF2α silencing was significantly reversed after HIF2α site 1 was excised, while the removal of site 2 or 3 had no significant reverse effect. The results suggested that site 1 was the main site of HIF2α regulation of NUDT1. In all, we can draw the conclusion that HIF2α directly transcriptionally regulates the expression of NUDT1 in ccRCC

### 3.7 Targeting NUDT1 can affect the drug sensitivity of ccRCC to sunitinib

Currently, the targeted therapies for HIF2α in ccRCC are mainly anti‐angiogenesis. They include various types of tyrosinase inhibitors. Among them, sunitinib is considered to be the first‐line drug for ccRCC treatment.^48^ In the above studies, we have confirmed that NUDT1 has a highly mediating effect on the biological function of HIF2α in ccRCC. Therefore, their correlation in the field of ccRCC treatment has become the focus of our attention. In order to explore the above conjecture, drug sensitivity experiments were carried out between sunitinib and NUDT1. Different concentrations of sunitinib were used to treat ccRCC cell lines with NUDT1 stably knocked down and NUDT1 stably overexpressed, and construct a drug sensitivity curve based on the experimental results. The experimental results showed that compared with the control cell lines, the ccRCC cell lines with stable knockdown of NUDT1 has a faster rate of decrease in cell viability against the same sunitinib concentration (Figure S14A). However, overexpression of NUDT1 will weak the inhibitory efficiency of ccRCC cell line against sunitinib (Figure S14B). These results all suggest that knocking down of NUDT1 in ccRCC can enhance the sensitivity of ccRCC to sunitinib, but overexpression of NUDT1 will have the opposite effect. Therefore, we can conclude that targeted knockdown of NUDT1 can enhance the drug sensitivity of ccRCC to sunitinib, which will provide the possibility for the development of new drug combination therapies.

### 3.8 NUDT1 knockdown suppresses the progression of ccRCC in vivo

**FIGURE 7:**
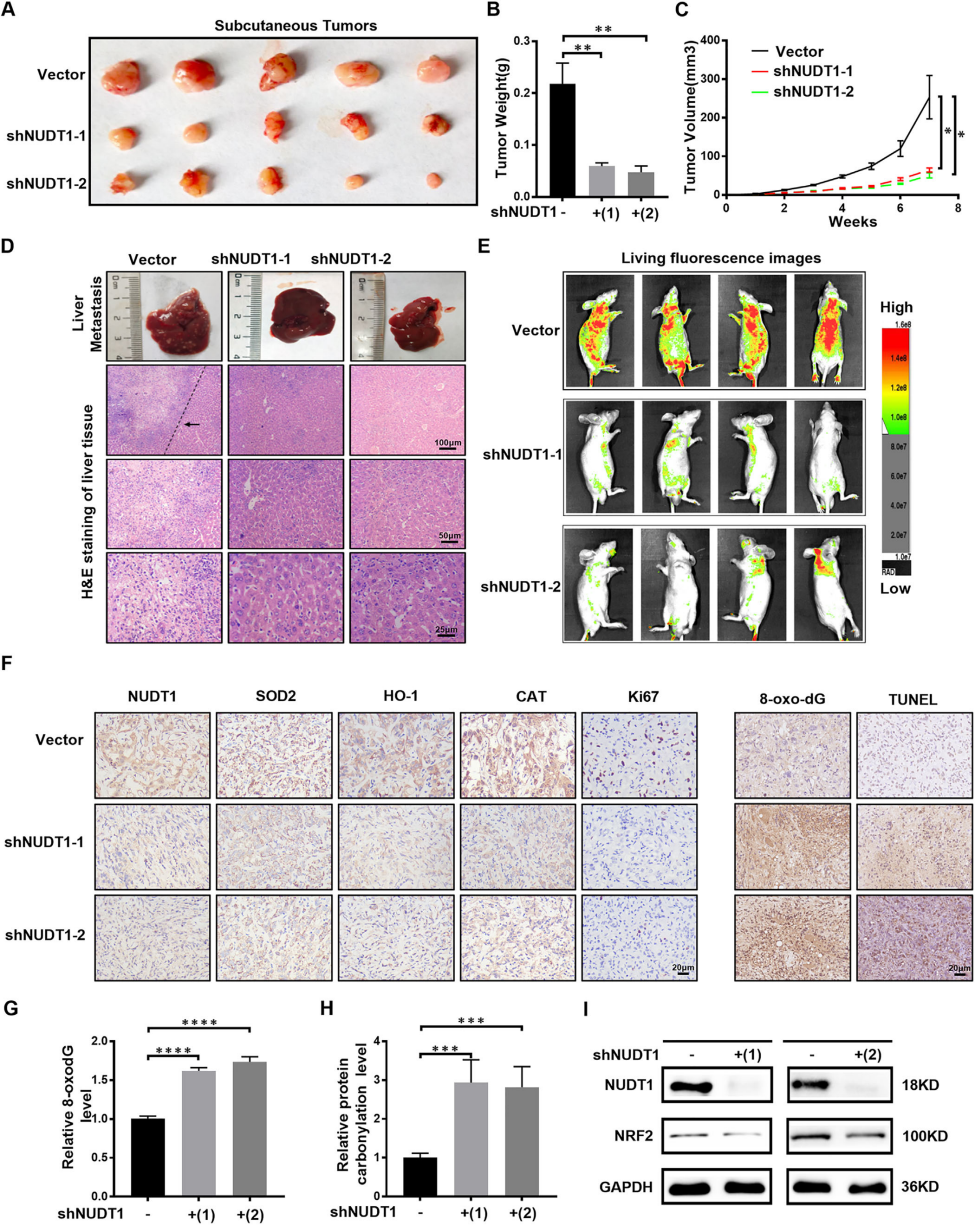
NUDT1 knockdown suppresses the progression of ccRCC in vivo. (A), (B) A498 cells and control cells transfected with shNUDT1‐1 and shNUDT1‐2 were injected subcutaneously into nude mice in the three groups. The tumour size and weight of mice in each group was measured after the seventh week. Data are expressed as the mean ± SEM from tumours of each group; *t*‐test, ****p < .0001, ***p < .001, **p < .01 and *p < .05 (independent‐samples *t*‐test for statistics). (C) The tumour volume of each group was measured every week. This graph is drawn based on the relationship between the number of weeks after tumour cell implantation and tumour size (mm^3^). Data are expressed as the mean ± SEM from tumours of each group; *t*‐test, ****p < .0001, ***p < .001, **p < .01 and *p < .05 (independent‐samples *t*‐test for statistics). (D) H&E staining of liver tissue in the NUDT1 knockdown group and control group. Scale bar: 100 μm, 50 μm, 25 μm. (E) Living fluorescence images of the NUDT1 knockdown in the metastasis model group and control group. (F) Immunohistochemical (IHC) staining for NUDT1, markers of oxidative stress‐related molecules (SOD2, HO‐1 and CAT), tumour malignancy (Ki67), 8‐oxodG and TUNEL in tumour xenografts. Scale bar: 20 μm. (G) Relative level of 8‐oxodG in xenograft tumour tissue; *t*‐test, ****p < .0001, ***p < .001, **p < .01 and *p < .05 (independent‐samples *t*‐test for statistics). (H) Relative level of protein carbonylation in xenograft tumour tissue; *t*‐test, ****p < .0001, ***p < .001, **p < .01 and * *p* < 0.05 (independent‐samples *t*‐test for statistics). (I) NRF2 protein level in xenograft tumour tissue determined by western blot

Encouraged by cell experiments, the role of NUDT1 at the animal level has become the focus of exploration. In order to explore the above functions, we used subcutaneous injection of tumour cells and tail vein injection to construct nude mouse subcutaneous xenograft tumour models and nude mouse‐tail vein metastasis models. Through the evaluation of subcutaneous transplanted tumours in nude mice, it is found that knocking down NUDT1 (Figure S15) can significantly inhibit the growth rate of tumours (Figure 7A–C). At the same time, small animal live imaging based on tail vein metastases in nude mice showed that knocking down NUDT1 can significantly reduce the level of tumour metastasis (Figure 7D, E). Subsequently, immunohistochemistry based on subcutaneous transplanted tumours also showed that the expression level of the corresponding antioxidant enzymes decreased significantly after knocking down NUDT1, and the tumour malignant index KI67 also decreased significantly. However, the DNA oxidative damage marker 8‐oxodG increased, and the level of apoptosis increased (Figure 7F). To verify the regulatory effect of silencing NUDT1 on oxidative stress, we detected the levels of 8‐oxodG and protein carbonylation in subcutaneous tumour tissues in nude mice. The results showed that the silencing of NUDT1 caused increased DNA oxidative damage and increased protein carbonylation levels in vivo (Figure 7G, H). Studies have shown that the transcription factor nuclear factor red blood cell 2 related factor 2 (NRF2), as the main regulator of the antioxidant response, can neutralise ROS in the cell to restore the cell's redox balance.^49^, ^50^, ^51^ Western blot results of xenograft tumour tissues showed that NUDT1 silencing led to a decrease in NRF2 levels in the body (Figure 7I). These results indicate that NUDT1 silence activates oxidative stress in vivo to inhibit the progression of ccRCC.

**FIGURE 8:**
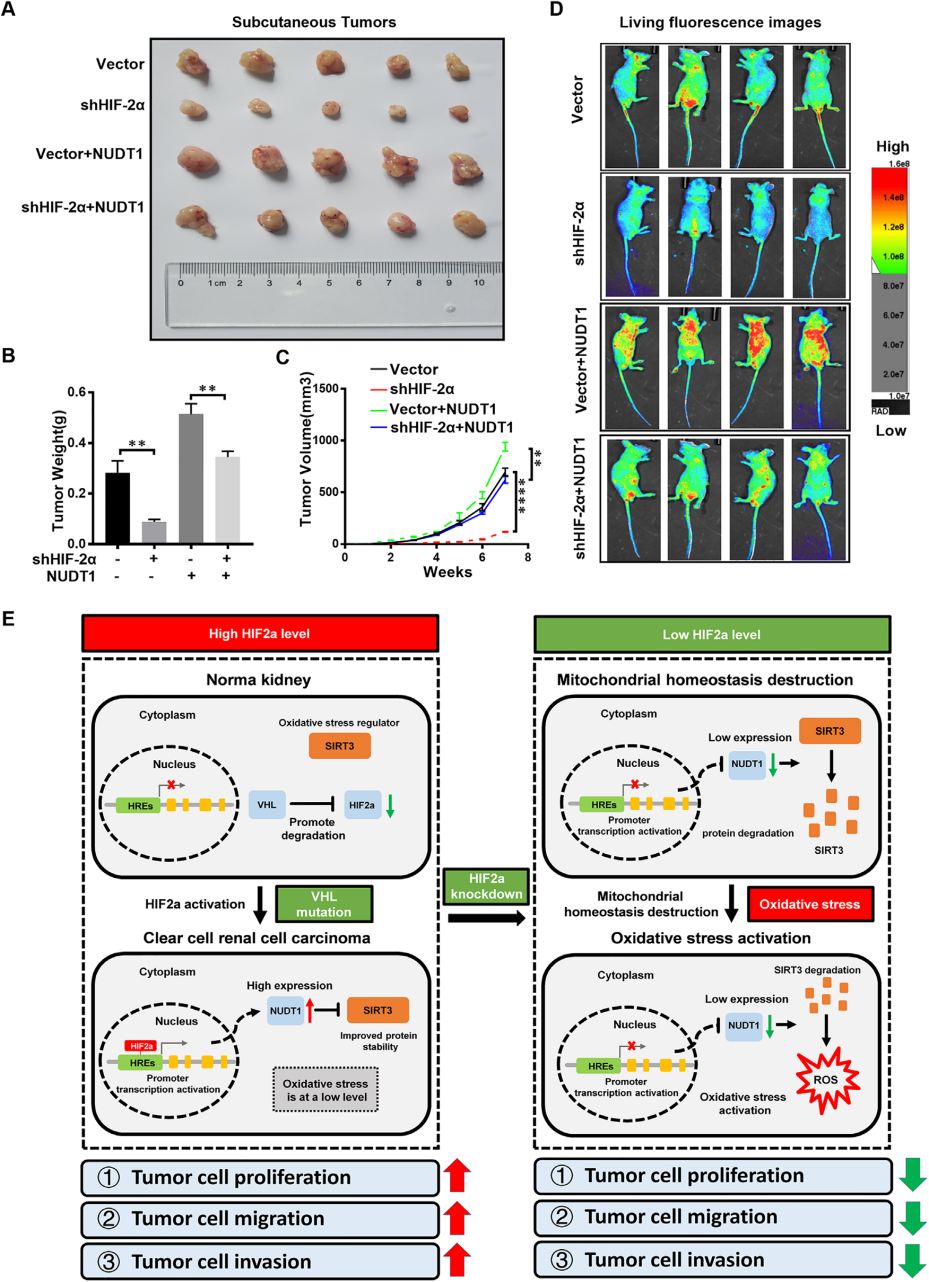
A model of HIF2α affecting oxidative stress through NUDT1 in ccRCC. (A) Xenograft tumour model was constructed by overexpressing NUDT1 lentivirus to infect HIF2α stable knockout and negative control A498 cells. (B) Xenograft tumour weight (g) after 7 weeks. (C) Volume growth curve of xenograft tumour within 7 weeks. (D) Living fluorescence images of the Vector, shHIF2α, Vector+NUDT1, shHIF2α+NUDT1 group in the metastasis model. (E) A model in which HIF2α acts as a transcription factor to directly increase NUDT1 expression by binding to the HIF2α response element in the NUDT1 promoter. The high expression of HIF2α in ccRCC cells can directly target the promotion of NUDT1 expression to stabilise SIRT3 protein in tumour cells and inhibit oxidative stress, thereby promoting the progression of ccRCC. When HIF2α is knocked down, it can target the reduction of NUDT1 expression and accelerate the degradation of SIRT3 protein in tumour cells to cause the increase of oxidative stress level and inhibiting the progression of ccRCC

In order to further prove in vivo that NUDT1 is a vital gene for HIF2α to promote ccRCC, HIF2α stable knockdown and negative control A498 cells were infected with NUDT1 overexpressing lentivirus. Xenograft tumour models and tail vein metastasis models were established using the above cell lines. Overexpression of NUDT1 can reverse the growth inhibition caused by HIF2α silencing, which is consistent with in vitro experiments (Figure 8A–C). The results of fluorescence images of living mice showed that the overexpression of NUDT1 reversed the metastasis inhibition effect caused by HIF2α silencing (Figure 8D). In general, NUDT1 is a vital downstream gene that HIF2α promotes the progression of ccRCC.

In summary, we constructed a model in which HIF2α acts as a transcription factor to directly elevate NUDT1 expression by binding the HIF2α response element in the NUDT1 promoter. NUDT1 regulates ccRCC progression through the SIRT3 stability mediated cellular oxidative stress. The decreased expression of HIF2α inhibits the expression of NUDT1 at the transcriptional level and causes the degradation of SIRT3 to accelerate, which cause an increase in tumour cell ROS and oxidative stress levels, thereby inhibits the progress of ccRCC (Figure 8E).

## 4 DISCUSSION

Multiple studies have indicated that oxidative stress takes a vital part in ccRCC.^36^, ^52^ CcRCC has the distinctive feature of HIF2α activation. HIF2α has been shown to affect oxidative stress.^53^ However, the unambiguous mechanism by which HIF2α affects oxidative stress is still unclear. Our study describes a new approach, HIF2α reduces the biological effects of oxidative stress in tumour cells through NUDT1. Malignant tumours can produce large amounts of ROS due to their high metabolic characteristics, leading to DNA damage and protein degeneration, thereby inhibiting tumour progression.^54^ HIF2α can directly transcriptionally activate the expression of NUDT1, reduce the biological impact of oxidative stress on tumour cells and promote tumour growth and metastasis.

Oxidative stress refers to the breakdown of the balance between the production of cellular oxidants and the removal of by‐products.^11^ Reactive oxygen species (ROS) is a barometer of oxidative stress and is produced during mitochondrial respiration. ROS occupies the central field in a variety of cell signaling pathways such as proliferation and apoptosis.^55^, ^56^ But, excess ROS can lead to structural damage in cells.^54^ Cancer cells produce more ROS than normal cells due to their active metabolism, and there is DNA damage.^10^ Therefore, tumour cells use some ‘means’ to reduce the level of ROS and the DNA damage it brings to promote cell survival and proliferation.^57^

Increased levels of ROS in cancer cells lead to an increase in 8‐oxo‐dGTP in the nucleic acid pools.^29^, ^58^, ^59^ NUDT1 protects the nucleic acid of cancer cells from oxidative damage by removing excess 8‐oxo‐dGTP.^60^ Related studies have pointed out that NUDT1 can reduce the level of ROS, which is induced by oncogenic RAS.^26^, ^61^, ^62^ Overexpressed NUDT1 promotes cancer cell growth and metastasis by reducing ROS levels and hydrolysing ROS products, such as 8‐oxo‐dGTP. Dozens of NUDT1 inhibitors have been developed with the goal of inhibiting cancer growth by accumulating oxidative damage in cancer cells.^29^, ^63^, ^64^, ^65^ Although these NUDT1 inhibitors have been shown to be effective in suppressing cancer, some studies have pointed out that NUDT1 inhibitors have failed to completely eradicate cancer cells.^66^, ^67^ In this context, we believe that the specific mechanism of inhibiting NUDT1 and the key factors affecting the efficiency of NUDT1 inhibition should be strictly resolved. The results of NUDT1 inhibition depend on whether there are strong oxidant driving factors. The presence of strong oxidant driver will eliminate the redundant function of NUDT1 inhibitors on tumours.^26^, ^61^ The oxidant driving factors in ccRCC may be the reason for the toxic effect of NUDT1 inhibition on tumour cells. Since gene depletion of NUDT1 and pharmacological inhibition of NUDT1 have different mechanisms of action, our study only reflects the effects of knockdown of NUDT1 on oxidative stress and cell function. As for the selection of effective NUDT1 inhibitors in ccRCC, follow‐up related experiments need to be supplemented. The role of HIF2α as a common transcription factor on the occurrence and development of ccRCC has been confirmed. Its effect on oxidative stress has also been discovered by a number of studies.^68^, ^69^ Under this premise, the specific mechanism by which HIF2α regulates oxidative stress is still confused. Our research further validated the effect of HIF2α on oxidative stress and clarified the specific ways in which HIF2α affects oxidative stress in ccRCC. Consistently, HIF2α directly transcriptionally activates NUDT1 to reduce the biological impact of oxidative stress. This regulation process may be an important link for HIF2α to promote the progress of ccRCC.

SIRT3 is a type III deacetylase that relies on nicotinamide‐adenine dinucleotide (NAD). It is mainly located in mitochondria and is widely distributed in tissues and organs rich in mitochondria such as kidney, brain and liver. It can play a vital role in the deacetylation of histones and non‐histone proteins in the regulation of cell metabolism, cell cycle, cell apoptosis and cell lifespan.^70^, ^71^ SIRT3 plays a pivotal role in cellular oxidative stress. It can deacetylate related acetylated proteins in mitochondria, stabilise mitochondrial function by increasing the activity of ROS scavenging enzymes, thereby inhibiting the accumulation of ROS in mitochondria to improve cell function.^72^ Our research found that NUDT1 in ccRCC can regulate the level of cellular oxidative stress by regulating the stability of SIRT3, thereby affecting the progress of ccRCC. This means that SIRT3 is an important downstream of NUDT1, and at the same time, treatments for SIRT3 can also provide the possibility for the further development of combined treatment programs.

Many proteins have a dual role in tumours due to the differences in their localisation and functional pathways in cells. They play different roles in different stages of tumour development and in different cells, such as YB‐1,^73^, ^75^ TRAP1,^76^, ^77^ autophagy pathways^77^, ^78^, ^79^ etc. According to literature reports, SIRT3 also has the same characteristics. Since SIRT3 can maintain the production of ROS at an appropriate level to prevent cell apoptosis and promote cell proliferation, it is called an oncogene in certain types of cancer.^80^ On the contrary, some studies have shown that SIRT3 has a tumour suppressor effect. According to reports, SIRT3 induces cell arrest and apoptosis by regulating Bcl‐2, HIF‐1α, p53 and other proteins.^81^, ^82^, ^83^, ^84^ At present, there is no consensus on the impact of SIRT3 on the occurrence and progression of ccRCC. The results of some studies are not completely consistent with ours, which is very likely to exist.^85^ Because the regulatory mechanisms of different studies are different, and we do not regulate the expression of SIRT3 at the transcriptional level, but affect the protein stability of SIRT3 through NUDT1. There may be some feedback and bypass adjustment mechanisms to produce functional differences. In view of these differences, we are expected to improve and resolve them through further experiments.

HIF2α as a far‐reaching ccRCC oncogene has been extensively studied in ccRCC. Most of these studies have focused on the angiogenic effects of HIF2α. Sunitinib, the first‐line treatment of ccRCC, targets vascular endothelial growth factor receptor (VEGFR) and platelet‐derived growth factor receptor (PDGFR), which are both downstream genes of HIF2α.^86^, ^87^ However, about 20% of patients with advanced RCC instinctively do not respond to sunitinib treatment, and patients who are sensitive to treatment gradually show drug resistance and tumour deterioration after 6–15 months of treatment.^88^ The cancer‐promoting effect of HIF2α on ccRCC is mainly due to angiogenesis and oxidative stress.^53^ Nevertheless, few reports have evaluated the effects of targeted oxidative stress therapy on ccRCC. Our research certified that NUDT1 is a pivotal gene through which HIF2α governs oxidative stress in ccRCC. Therefore, we believe that treatments targeting NUDT1 are of great benefit to inhibit the progression of ccRCC. In view of the above findings, we come up with a brand‐new drug joint tactics: a combination therapy of NUDT1 targeting inhibitors and anti‐angiogenesis.

## 5 CONCLUSION

In summary, we found a novel pathway for HIF2α to transcriptionally activate the expression of NUDT1 in ccRCC. As HIF2α’s downstream, NUDT1 mediates the stability of SIRT3 to influence the process of cell oxidative stress and regulate ccRCC. Moreover, our research provides a new direction for the cancer‐promoting effect of HIF2α in ccRCC, which is different from angiogenesis. This may become a new weapon to break through the outcome of ccRCC‐targeted drug treatment resistance.

## CONFLICT OF INTEREST

The authors declare that they have no competing interests.

## Supporting information

Supplementary information 1

Supplementary information 2

Supplementary information 3

Supplementary information 4
