# Supplementary material for: HIF2α promotes tumour growth in clear cell renal cell carcinoma by increasing the expression of NUDT1 to reduce oxidative stress
Source: Clin Transl Med. 2021 Nov 4;11(11):e592. doi: 10.1002/ctm2.592 (PMC8567048; doi:10.1002/ctm2.592)
Supplement: Supplementary file 1 — Supplementary information 1 [file CTM2-11-e592-s003.docx]

**Supplementary information 1**

**
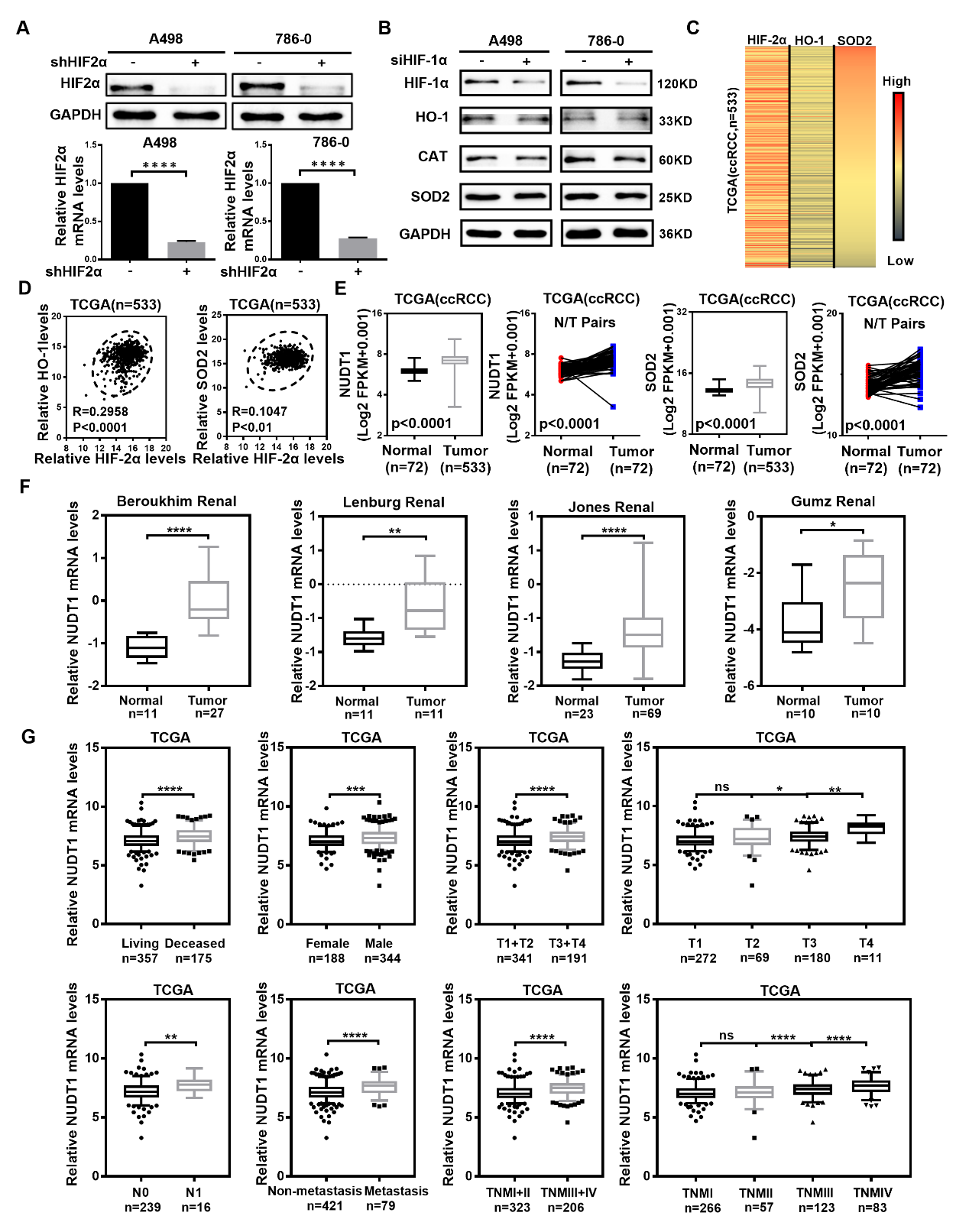
**

**Supplementary Figure S1. NUDT1 expression is highly correlated with HIF2****α expression and ccRCC clinical pathological parameters.**

**A.** Western blotting and qPCR were used to verify HIF2α knockdown at the protein and mRNA levels, respectively; t-test, * P <0.05, *** P <0.001 and **** P <0.0001(Independent-Samples t-test for statistics).

**B.** Protein expression levels of HO-1, CAT and SOD2 in HIF1α knockdown A498 and 786-0 cells were shown by western blotting.

**C-D.** The correlation heatmap and the linear correlation curve between HIF2α and the most critical molecules related to oxidative stress (HO-1 and SOD2) based on the data from the TCGA-KIRC database (r stands for Pearson correlation coefficient).

**E.** The NUDT1 and SOD2 mRNA levels in 533 ccRCC tissues and 72 paired tissues in the TCGA database; t-test, P <0.0001(Independent-Samples t-test for overall difference analysis, Paired-Samples t-test for N/T pairs analysis).

**F.** The expression of NUDT1 is shown from the Beroukhim renal, Lenburg renal, Jones renal, and Gumz renal gene sets from the Oncomine database; t-test, P <0.0001(Independent-Samples t-test for statistics).

**G.** The correlations between NUDT1 expression and different clinicopathological parameters of ccRCC are based on the relevant data of 519 ccRCC patients with complete clinical parameters from the TCGA-KIRC database; t-test, **** P <0.0001, *** P <0.001, ** P <0.01, and * P <0.05; ns, P≥0.05 (Independent-Samples t-test for statistics).


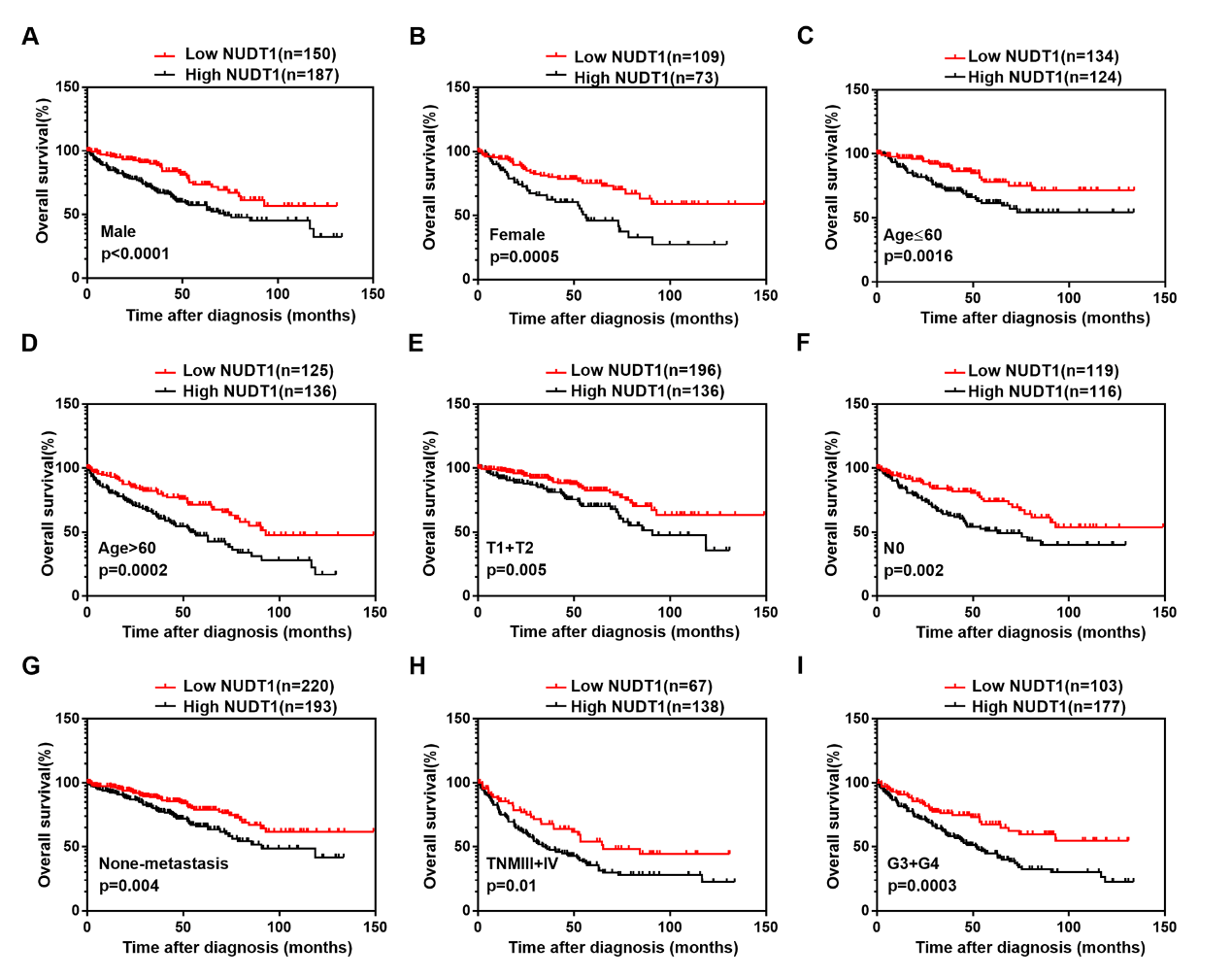


**Supplementary Figure S2. High expression of NUDT1 is associated with poor overall survival in patients with ccRCC.**

Kaplan-Meier analysis was performed according to different ccRCC clinical stages to obtain (A-I) OS curves. The p value is obtained through Log-rank (Mantel-Cox) test. (A) Male. (B) Female. (C) Age ≤ 60. (D) Age > 60. (E) T1+T2 stage. (F) N0 stage. (G) Nonmetastasis. (H) TNM III+ IV stage. (I) G3+G4 stage.

**
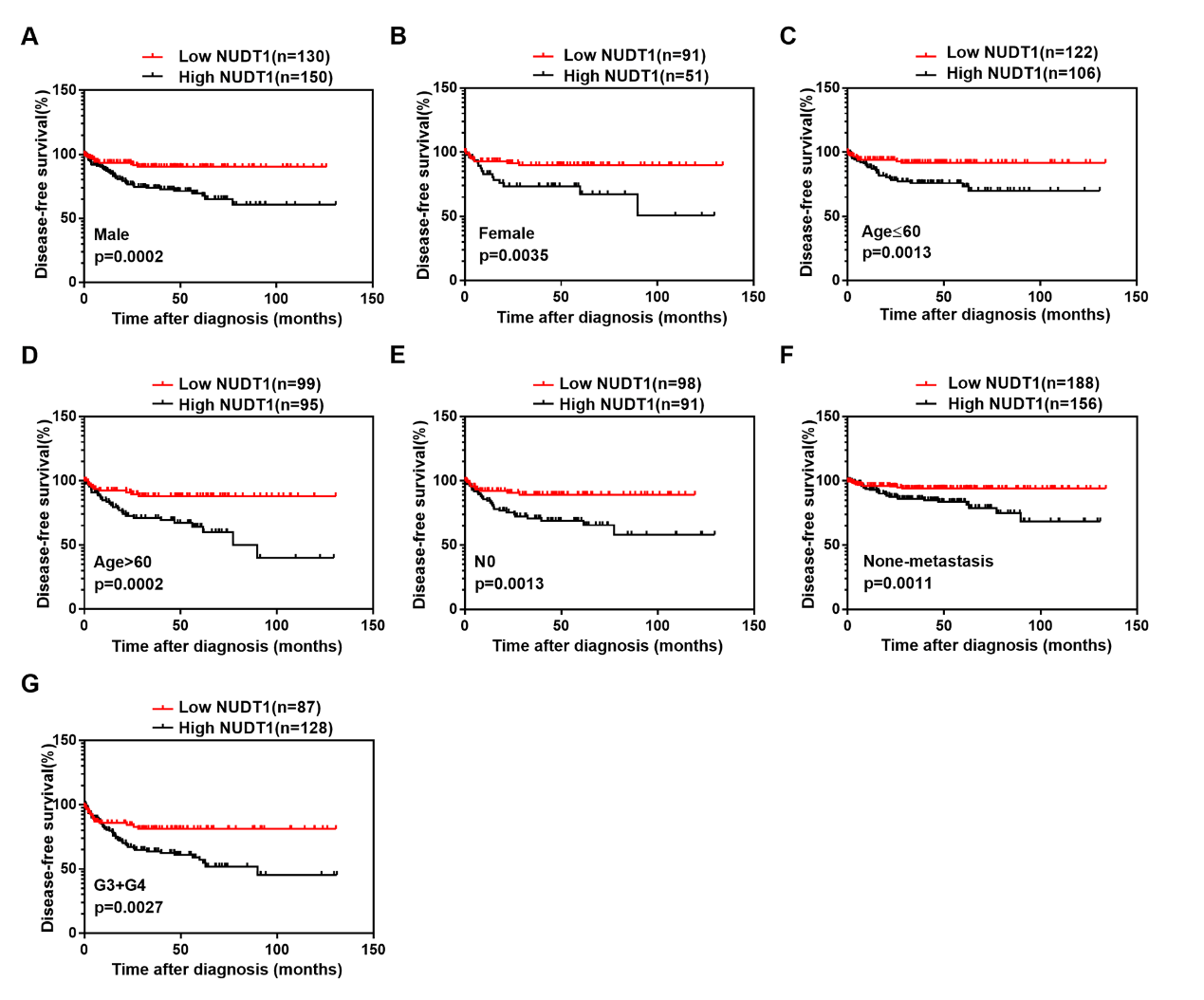
**

**Supplementary Figure S3. High expression of NUDT1 is associated with** **poor disease-free survival in patients with ccRCC.**

Kaplan-Meier analysis was performed according to different ccRCC clinical stages to obtain (A-G) disease-free survival curves. The p value is obtained through Log-rank (Mantel-Cox) test. (A) Male. (B) Female. (C) Age ≤ 60. (D) Age > 60. (E) N0 stage. (F) Nonmetastasis. (G) G3+G4 stage.

**
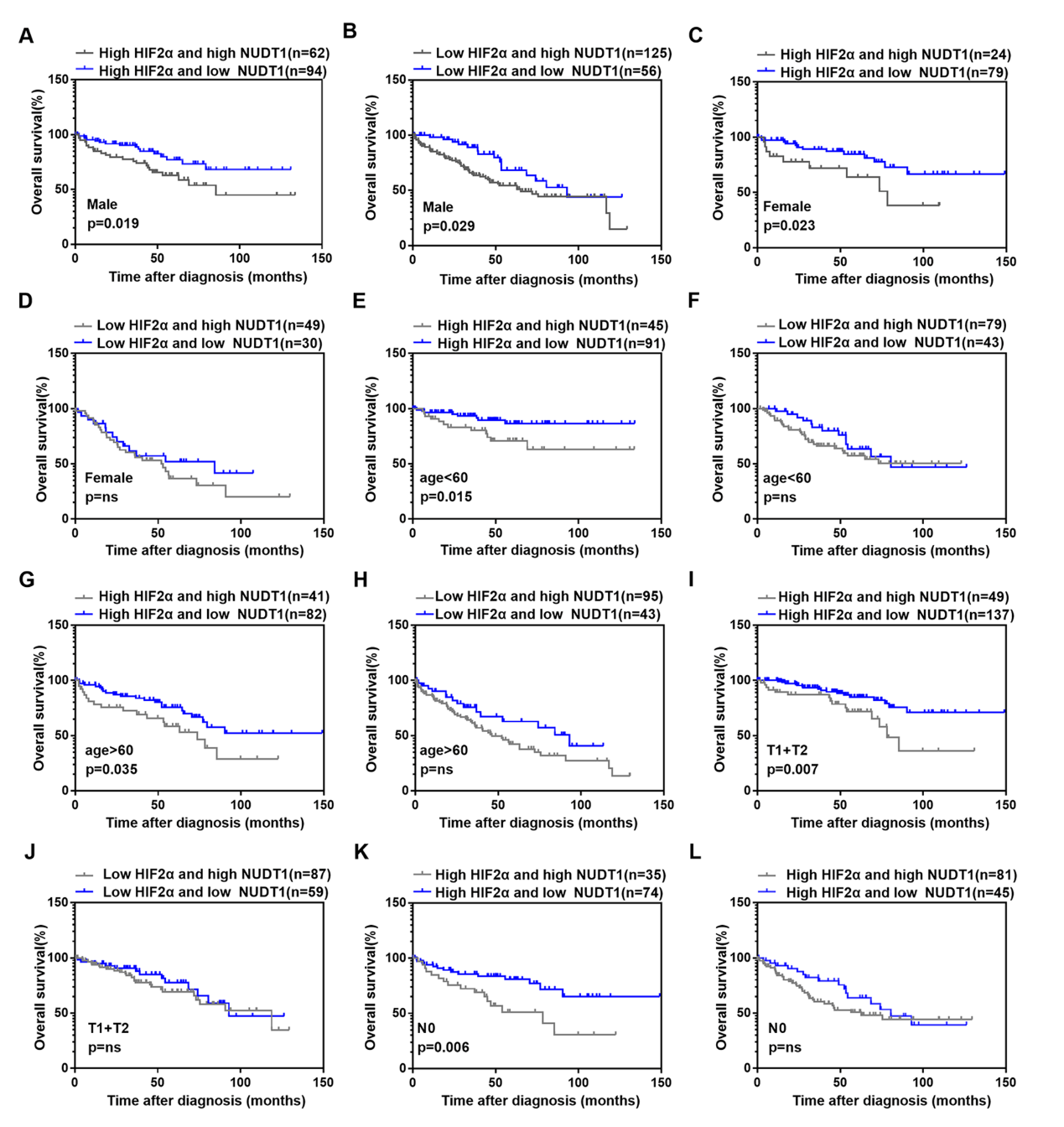
**

**Supplementary Figure S4. The effect of NUDT1 expression on overall survival is more significant in the high expression HIF2α group than in the low expression HIF2α group.** CcRCC patient data are divided into four groups based on the expression levels of HIF2α and NUDT1.

Kaplan-Meier analysis was performed according to different ccRCC clinical stages to obtain (A-L) OS curves. The p value is obtained through Log-rank (Mantel-Cox) test. (A) HIF2α high expression, Male. (B) HIF2α low expression, Male. (C) HIF2α high expression, Female. (D) HIF2α low expression, Female. (E) HIF2α high expression, Age＜60. (F) HIF2α low expression, Age＜60. (G) HIF2α high expression, Age>60. (H) HIF2α low expression, Age>60. (I) HIF2α high expression, T1+T2. (I) HIF2α low expression, T1+T2. (K) HIF2α high expression, N0. (L) HIF2α low expression, N0.

**
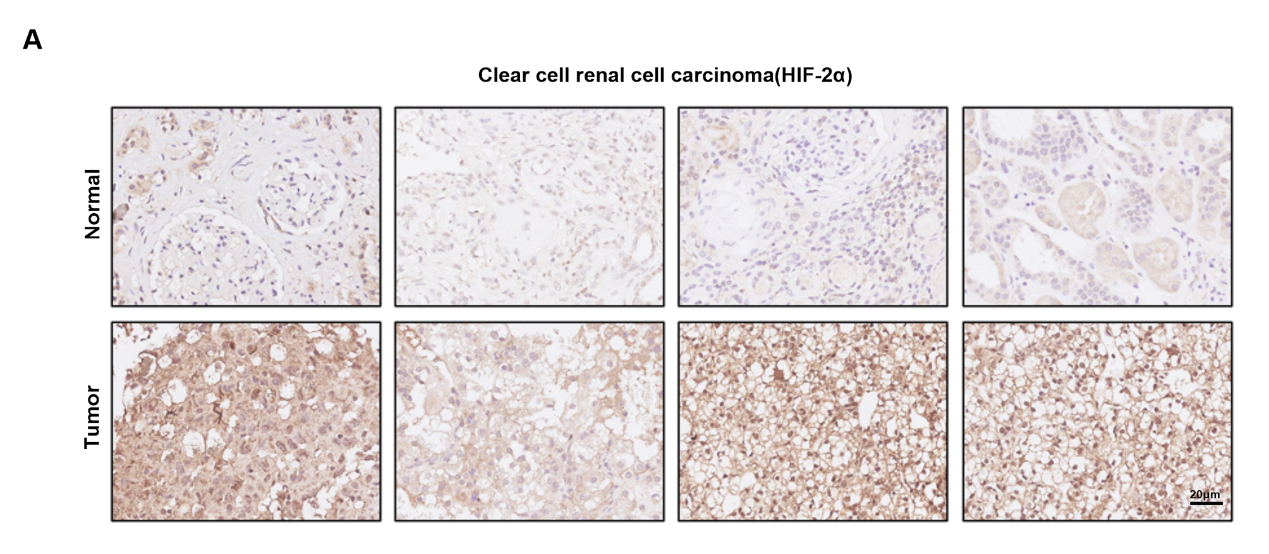
**

**Supplementary Figure S5.** **HIF2α is highly expressed in ccRCC.**

**A.** Immunohistochemical (IHC) staining for HIF2α in ccRCC tissues and adjacent nonmalignant tissues. Scale bar: 20μm.

**
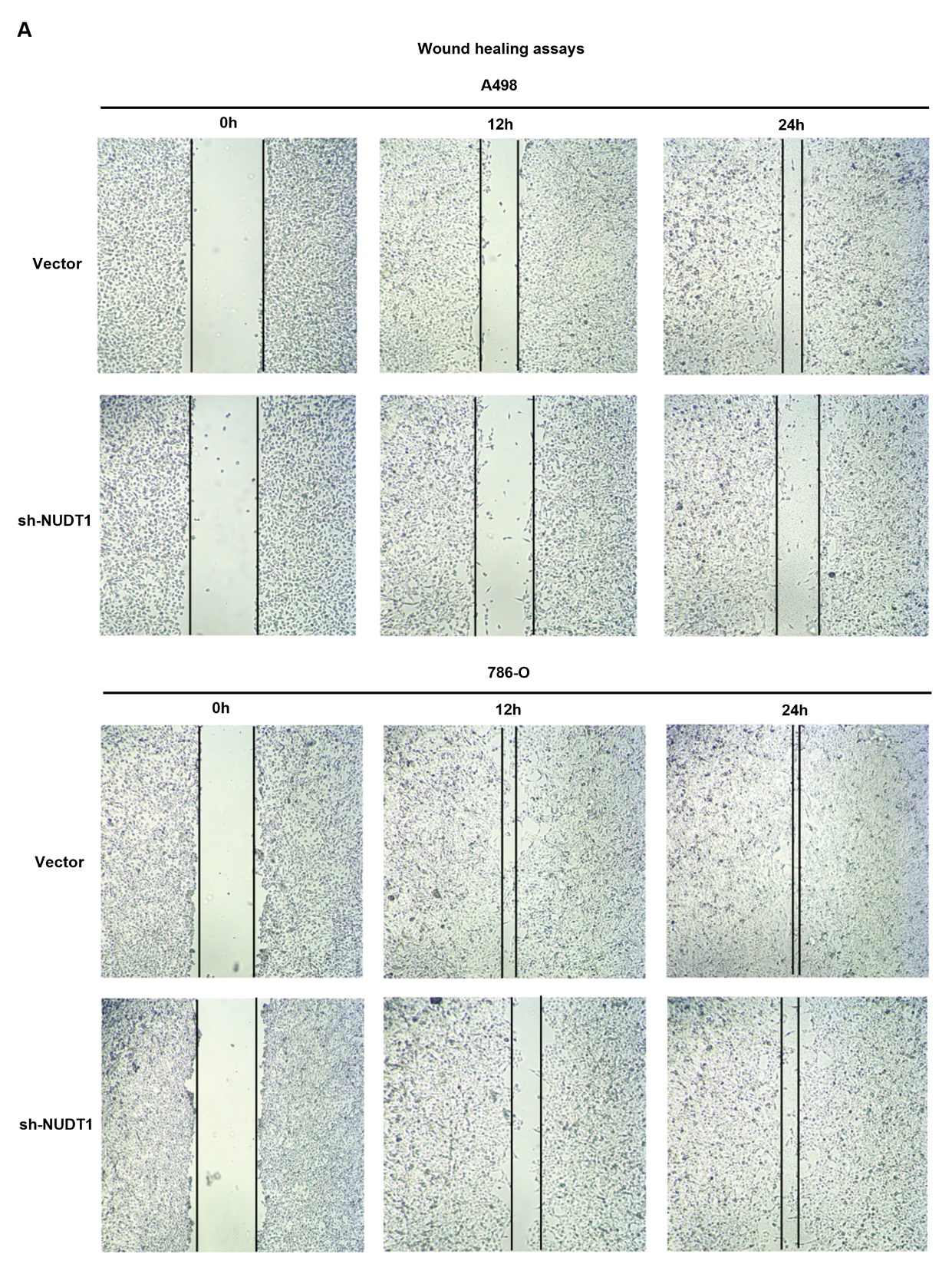
**

**Supplementary Figure S6. Knockdown of NUDT1 can inhibit the migration of ccRCC cells.** (A) Wound healing experiments were used to detect the migration of NUDT1 knockdown cells.


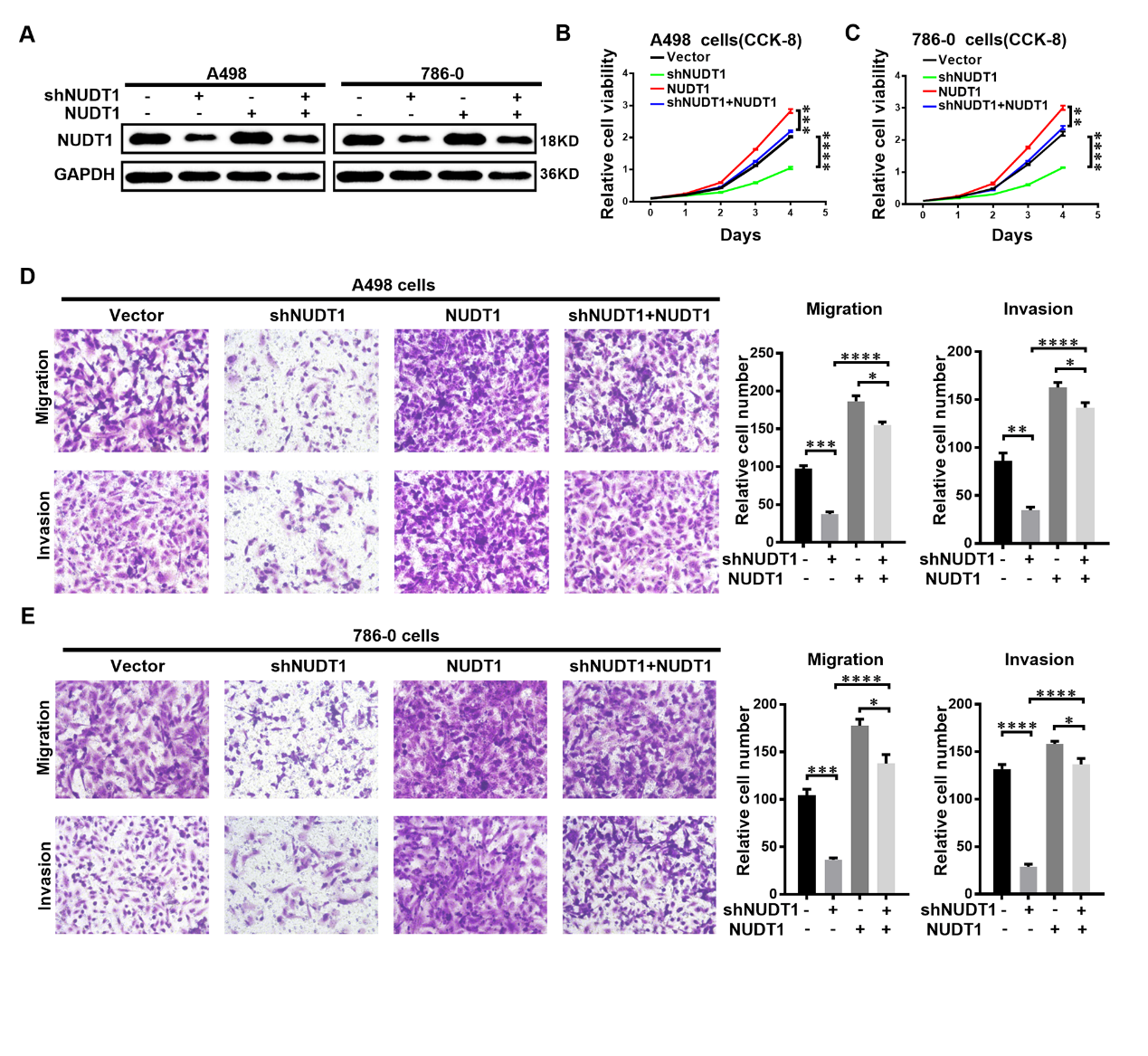


**Supplementary Figure S7. Functional recovery experiment of overexpression of NUDT1 in stable silencing NUDT1 cells.**

**A.** Protein levels of NUDT1 in Double transfected cells were shown by western blotting.

**B-C.** CCK8 proliferation curve of functional recovery cells; t-test, **** P <0.0001, *** P <0.001, ** P <0.01, and * P <0.05 (Independent-Samples t-test for statistics).

**D-E.** The results of the transwell assay of the migration and invasion of functional recovery cells.


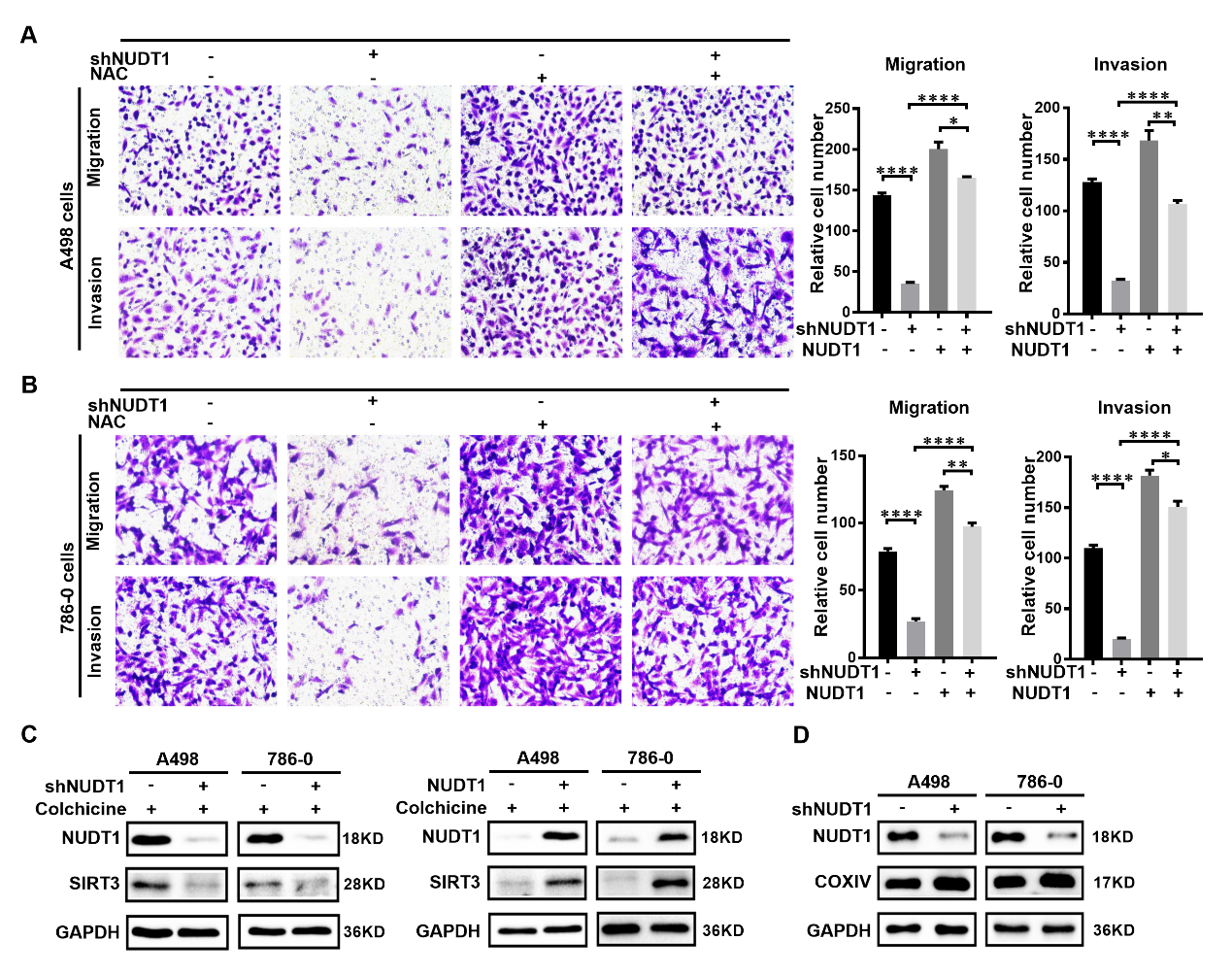


**Supplementary Figure S8. NUDT1 regulates the progress of ccRCC by inhibiting the ubiquitination of SIRT3 to affect cellular oxidative stress.**

**A.** The results of the transwell assay of the migration and invasion of functional recovery A498 cells.

**B.** The results of the transwell assay of the migration and invasion of functional recovery 786-0 cells.

**C.** Protein levels of SIRT3 in NUDT1 knockdown and overexpressing cells treated with 4μM colchicine for 6h were shown by western blotting.

**D.** The protein levels of mitochondrial marker COXIV after knocking down NUDT1 are shown by western blotting.


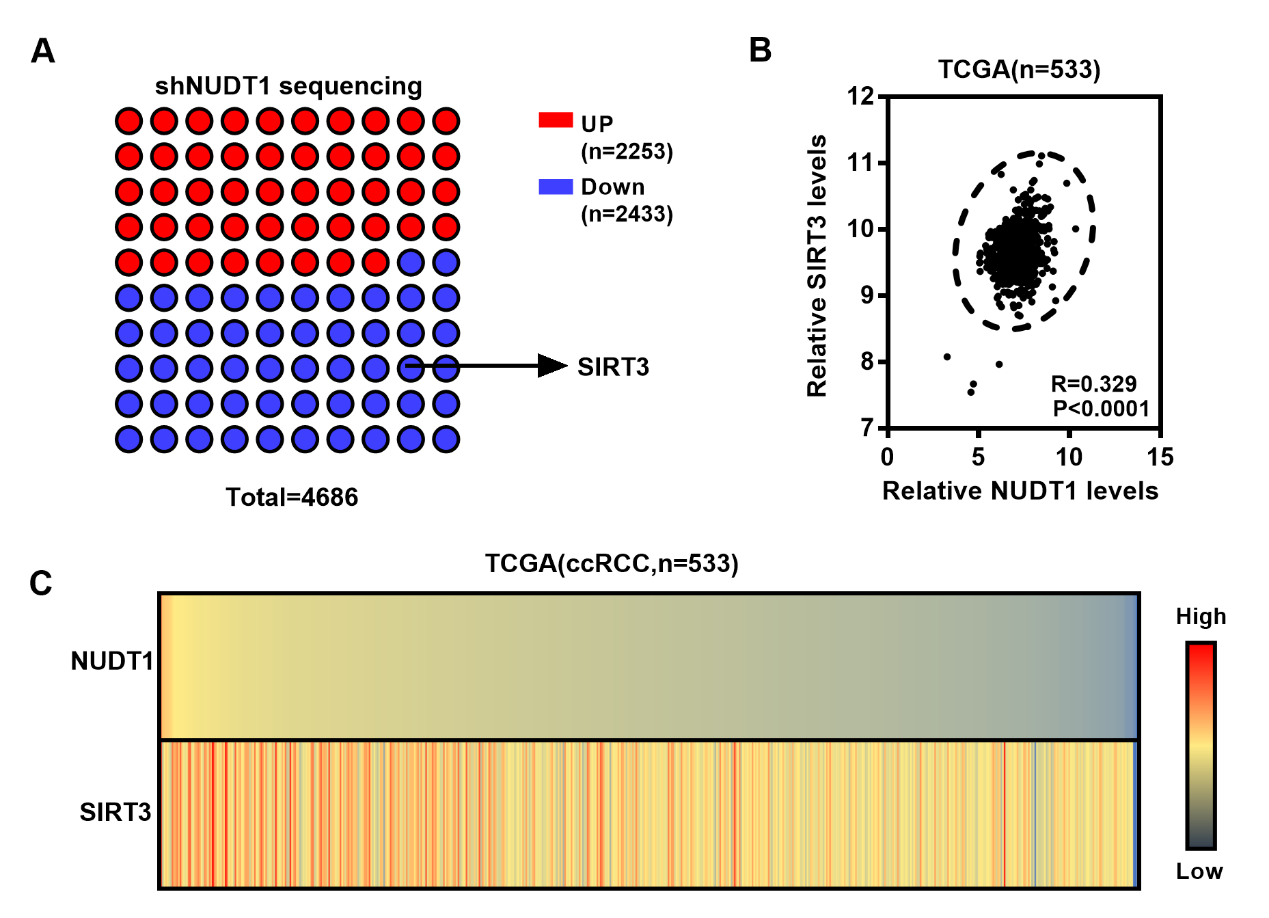


**Supplementary Figure S9. SIRT3 is highly correlated with NUDT1.**

**A.** Whole transcriptome sequencing data after NUDT1 knockdown. After NUDT1 knockdown, there were 4686 meaningful differentially expressed genes, of which 2253 were up-regulated genes and 2433 were down-regulated genes (including NUDT1).

**B.** The linear correlation curve (R stands for Pearson correlation coefficient) between NUDT1 and SIRT3 based on the data from the TCGA-KIRC database.

**C.** The correlation heatmap between NUDT1 and SIRT3 based on the data from the TCGA-KIRC database.


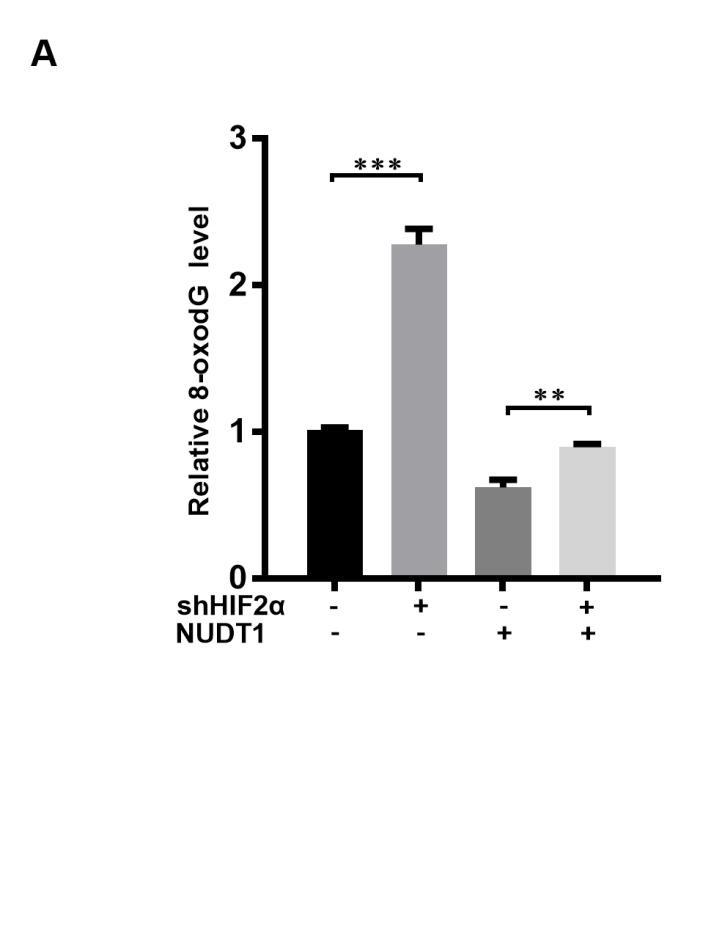


**Supplementary Figure S10. NUDT1 is the downstream molecule of HIF2α to reduce oxidative damage.**

**A.** Relative levels of 8-oxodG in functional recovery cell lines; t-test, **** P <0.0001, *** P <0.001, ** P <0.01, and * P <0.05 (Independent-Samples t-test for statistics).


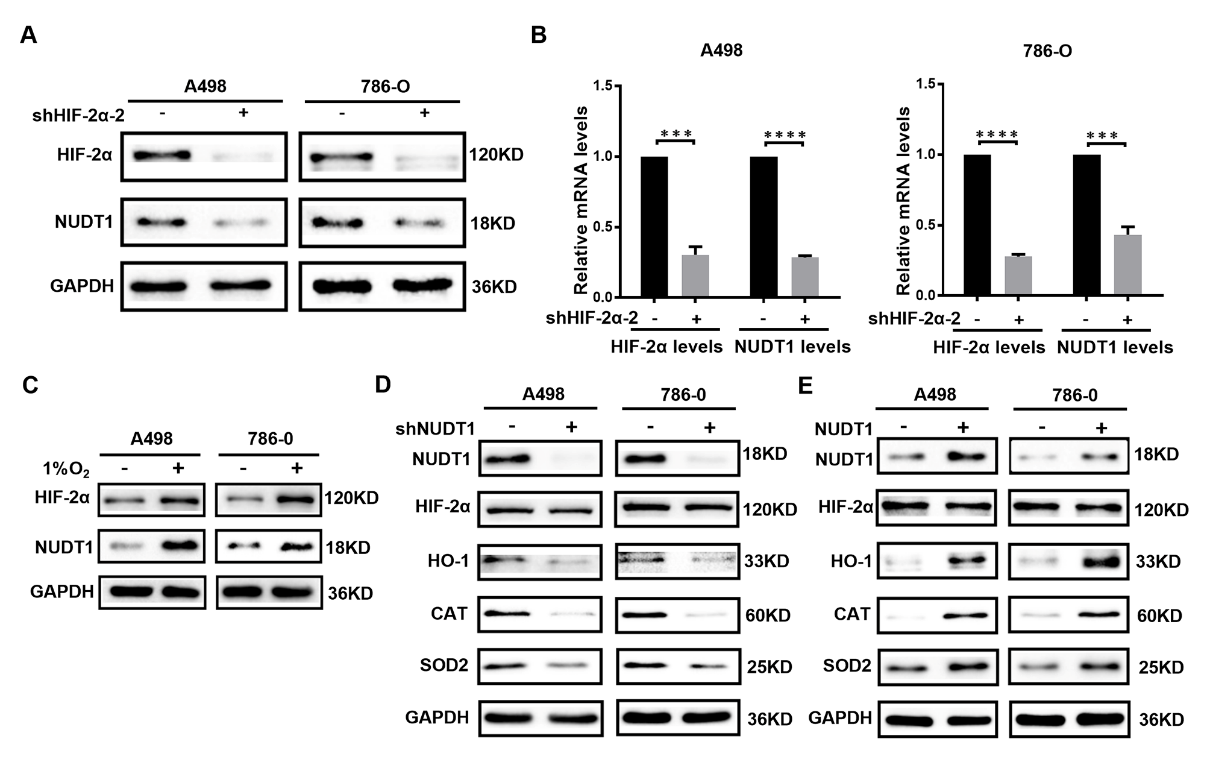


**Supplementary Figure S11. NUDT1 is a downstream gene of HIF2α**

**A-B**. The protein and mRNA levels of NUDT1 after knocking down HIF2α with shHIF2α-2 are shown by western blotting and qPCR; t-test, * P <0.05, *** P <0.001 and **** P <0.0001(Independent-Samples t-test for statistics).

**C.** Protein levels of HIF2α and NUDT1 in ccRCC cell lines cultured under 1% O_2_.

**D.** The protein levels of HIF2α, HO-1, CAT and SOD2 in NUDT1 knocked down cells.

**E.** The protein levels of HIF2α, HO-1, CAT and SOD2 in NUDT1 overexpressed cells.

**
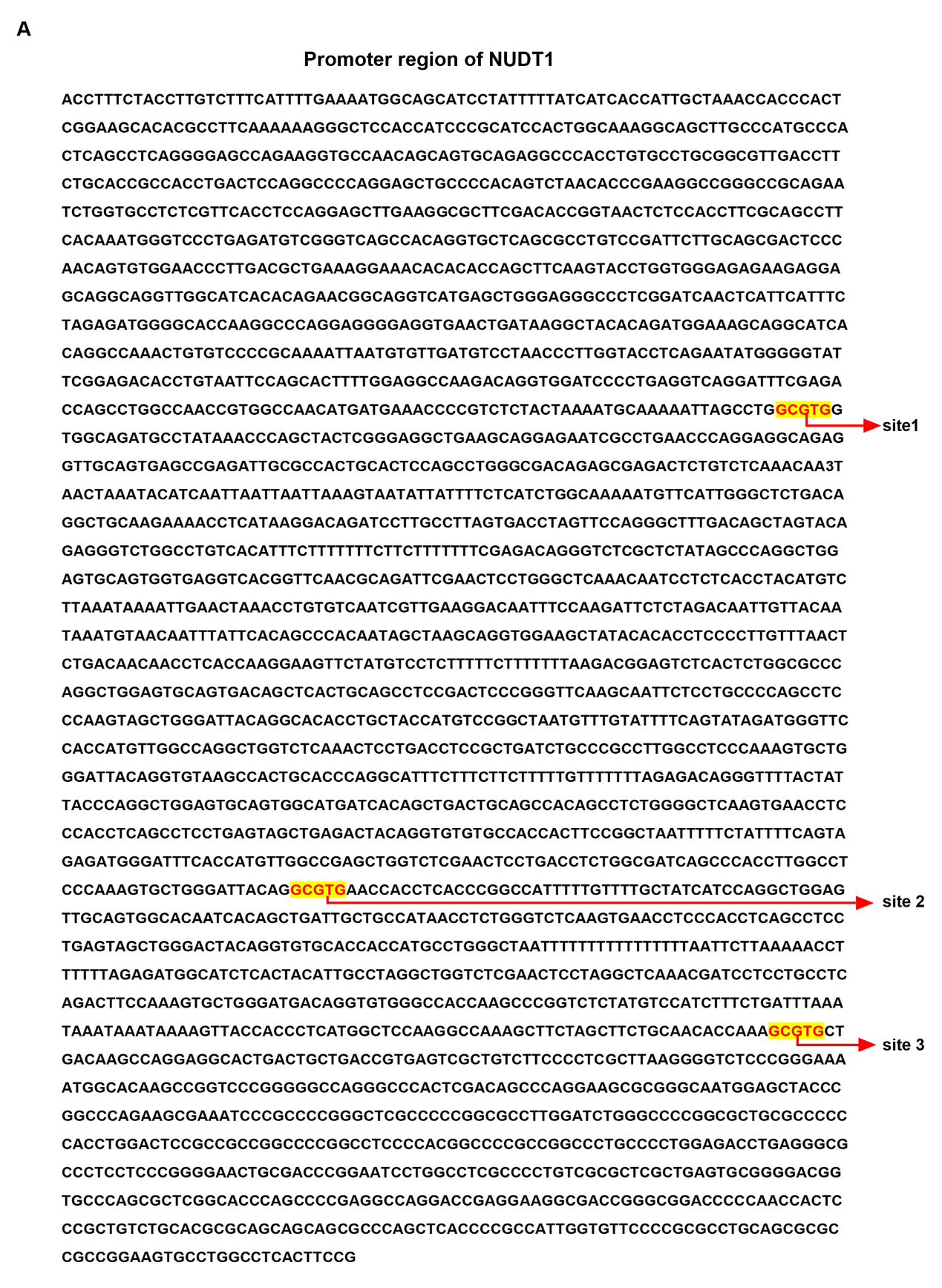
**

**Supplementary Figure S12. Potential binding sites for HIF2α in the NUDT1 promoter region.** The 3000 bp region upstream of the NUDT1 promoter identified three potential binding sites (1, 2, and 3) based on HIF2α binding sequences.


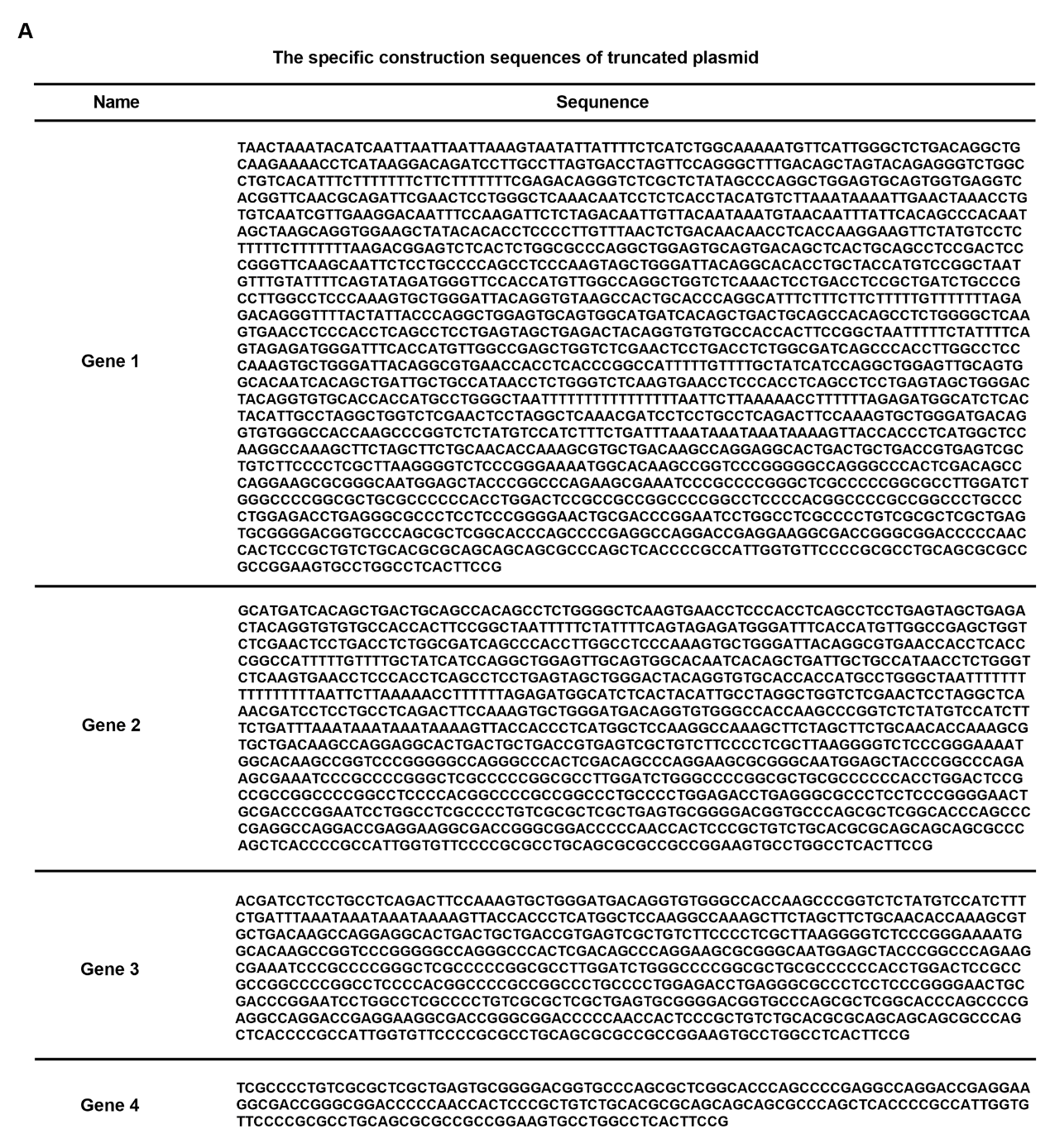


**Supplementary Figure S13. The specific construction sequences of truncated plasmid.**

The sequence of the truncated and full-length NUDT1 promoter luciferase reporter plasmids. Gene 1 is the sequence of the full-length plasmid, gene2, gene3 and gene4 are the sequence of the truncated plasmid.


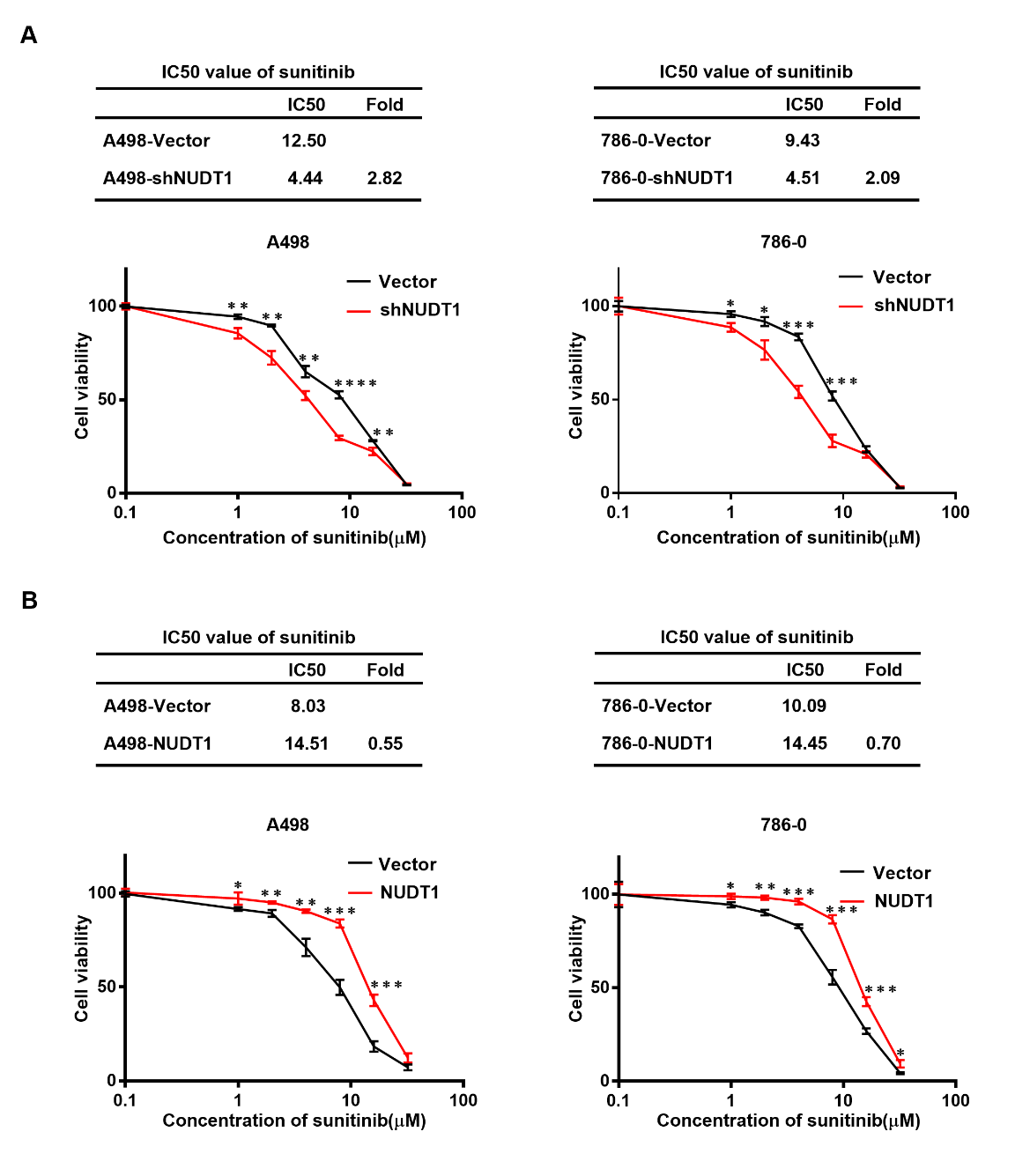


**Supplementary Figure S14. Targeting NUDT1 can affect the drug sensitivity of ccRCC to sunitinib.**

**A.** Drug sensitivity curves and IC50 of sunitinib on NUDT1 knockdown cells and negative control cells.

**B.** Drug sensitivity curves and IC50 of sunitinib on NUDT1 overexpressed cells and negative control cells.


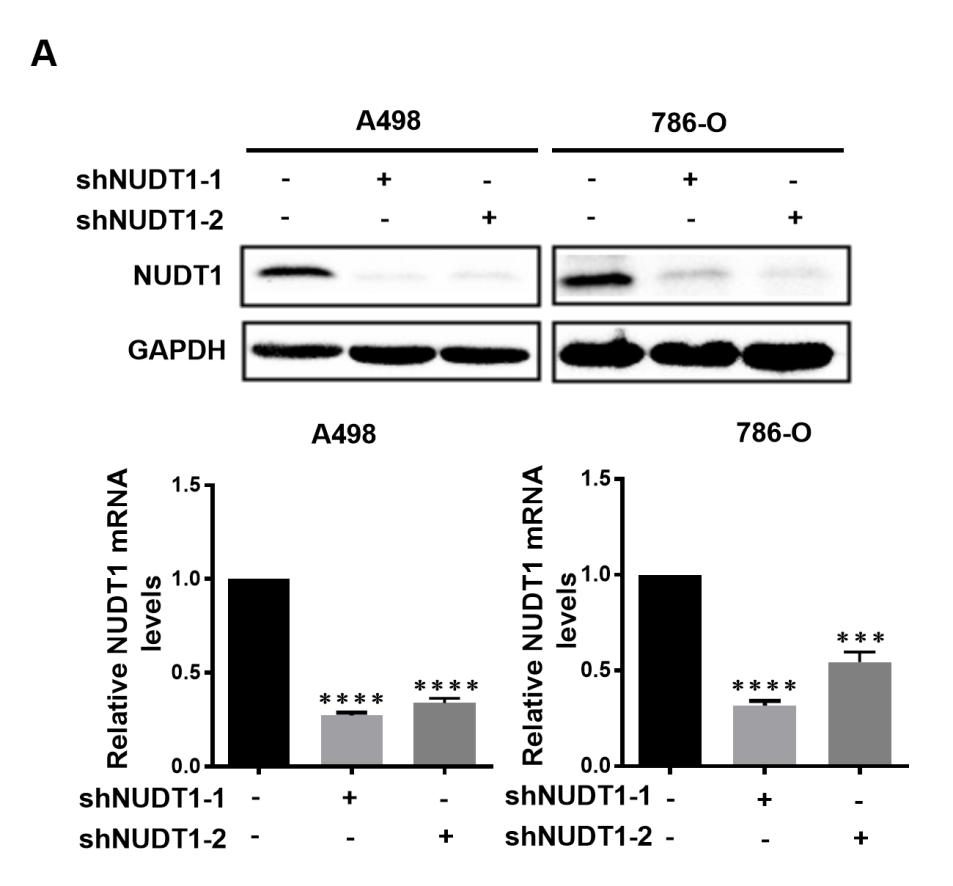


**Supplementary Figure S15. Validation of the effect of knocking down NUDT1.**

**A.** Western blotting and qPCR verified the knockdown effects of shNUDT1-1 and shNUDT1-2 on NUDT1 at protein and mRNA levels, respectively; t-test, * P <0.05, *** P <0.001 and **** P <0.0001(Independent-Samples t-test for statistics).
