# Supplementary material for: HIF2α promotes tumour growth in clear cell renal cell carcinoma by increasing the expression of NUDT1 to reduce oxidative stress
Source: Clin Transl Med. 2021 Nov 4;11(11):e592. doi: 10.1002/ctm2.592 (PMC8567048; doi:10.1002/ctm2.592)
Supplement: Supplementary file 2 — Supplementary information 2 [file CTM2-11-e592-s004.docx]

**Supplementary information 2**

**
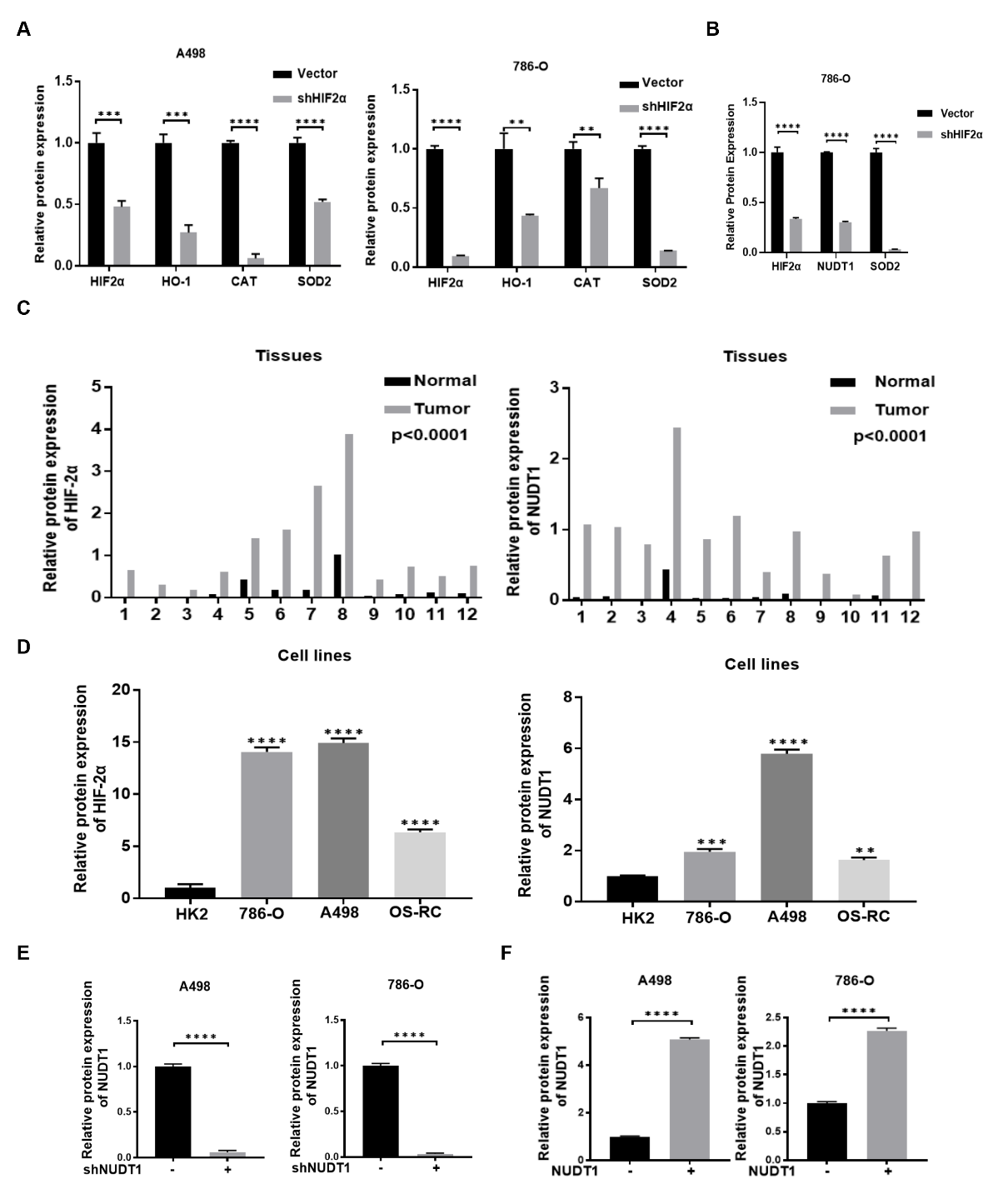
**

**Western blot statistics.** (A) Figure 1B. (B) Figure 1D. (C) Figure 1I. (D) Figure 1K. (E) Figure 2A. (F) Figure 2B.


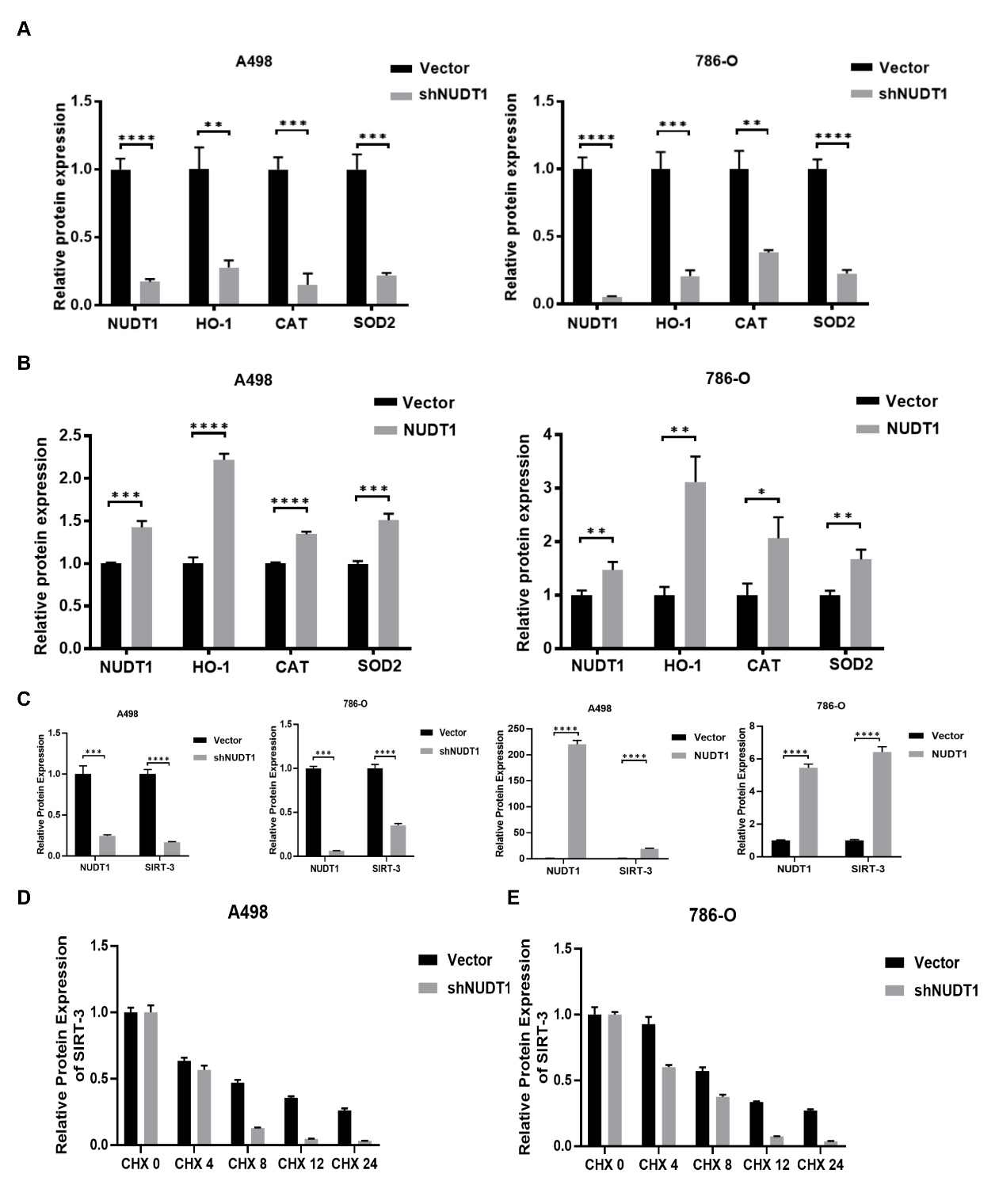


**Western blot statistics.** (A) Figure 3D. (B) Figure 3E. (C) Figure 4C. (D) Figure D. (E) Figure E.

**
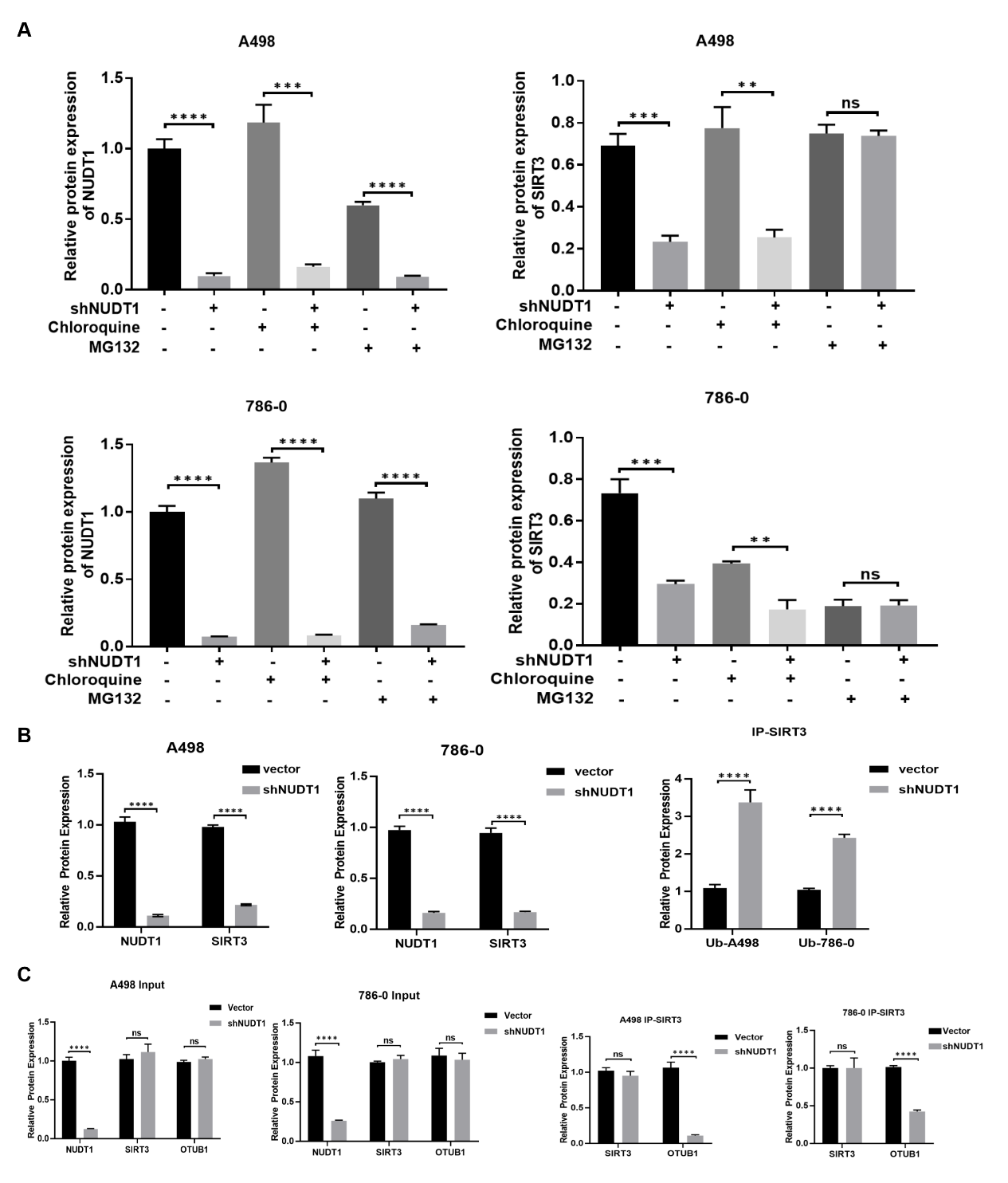
**

**Western blot statistics.** (A) Figure 4F. (B) Figure 4G. (C) Figure 4H.


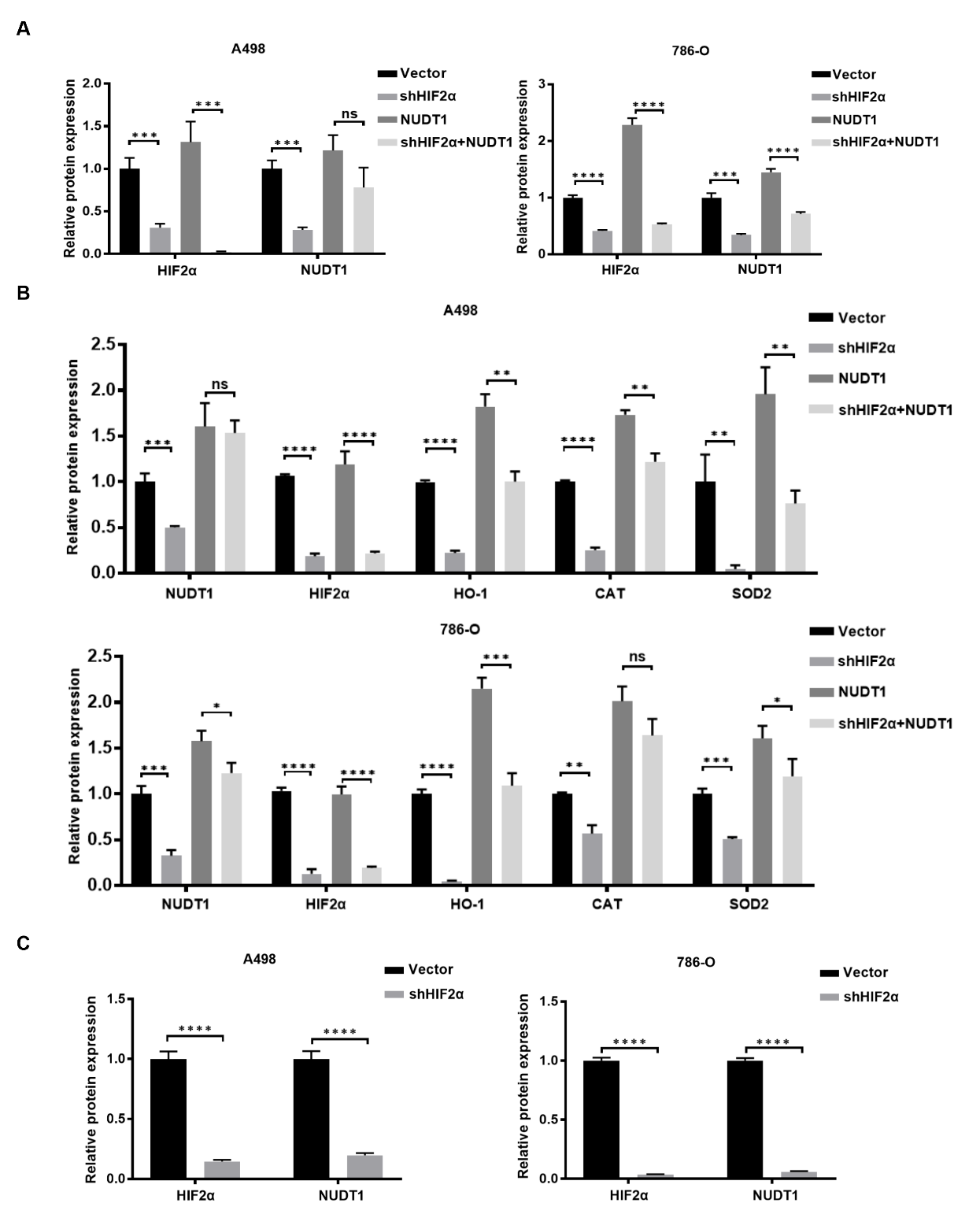


**Western blot statistics.** (A) Figure 5A. (B) Figure 5C. (C) Figure 6C.


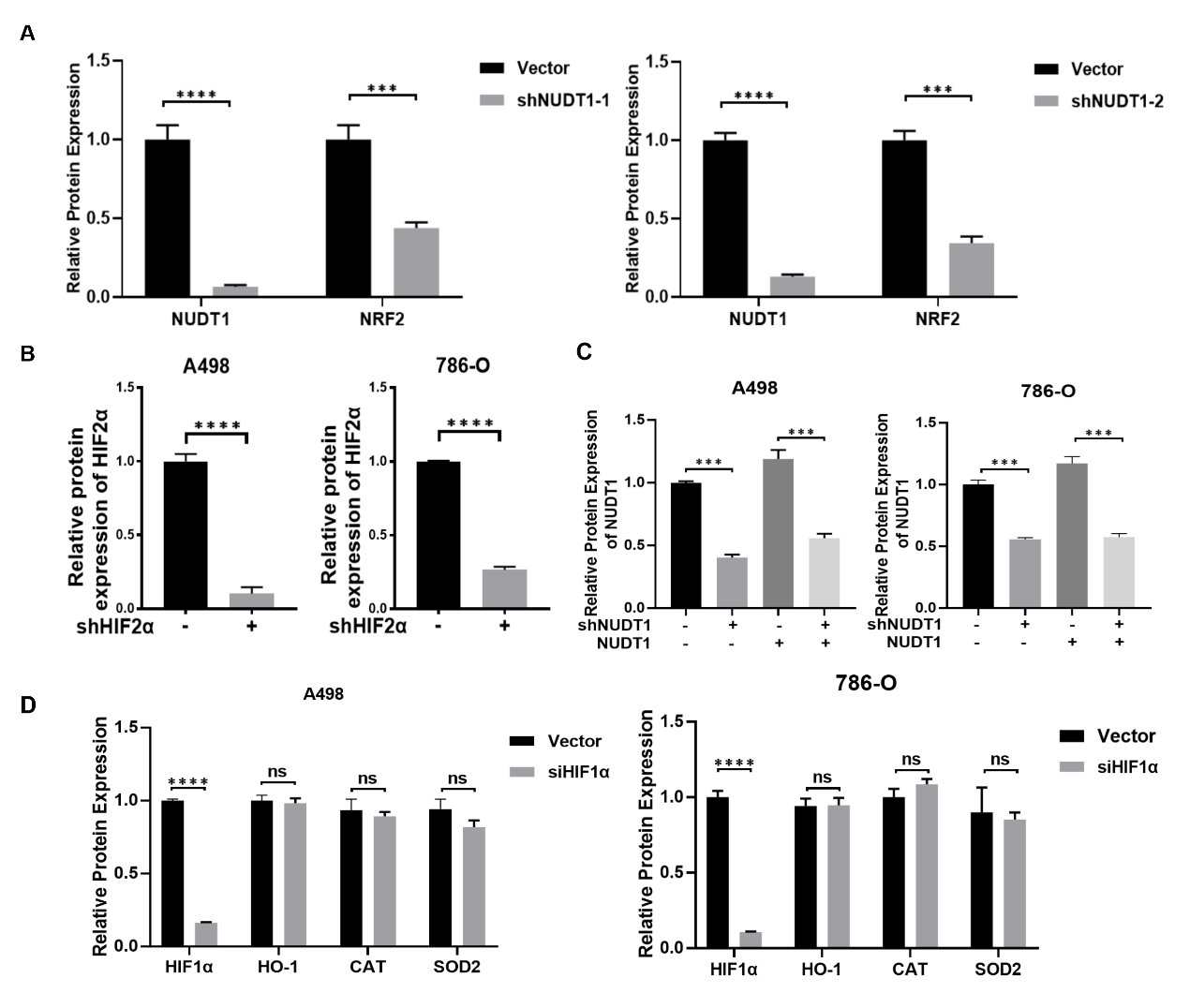


**Western blot statistics.** (A) Figure 7I. (B) Supplementary figure S1A. (C) Supplementary figure S7A. (D) Supplementary figure S1B.


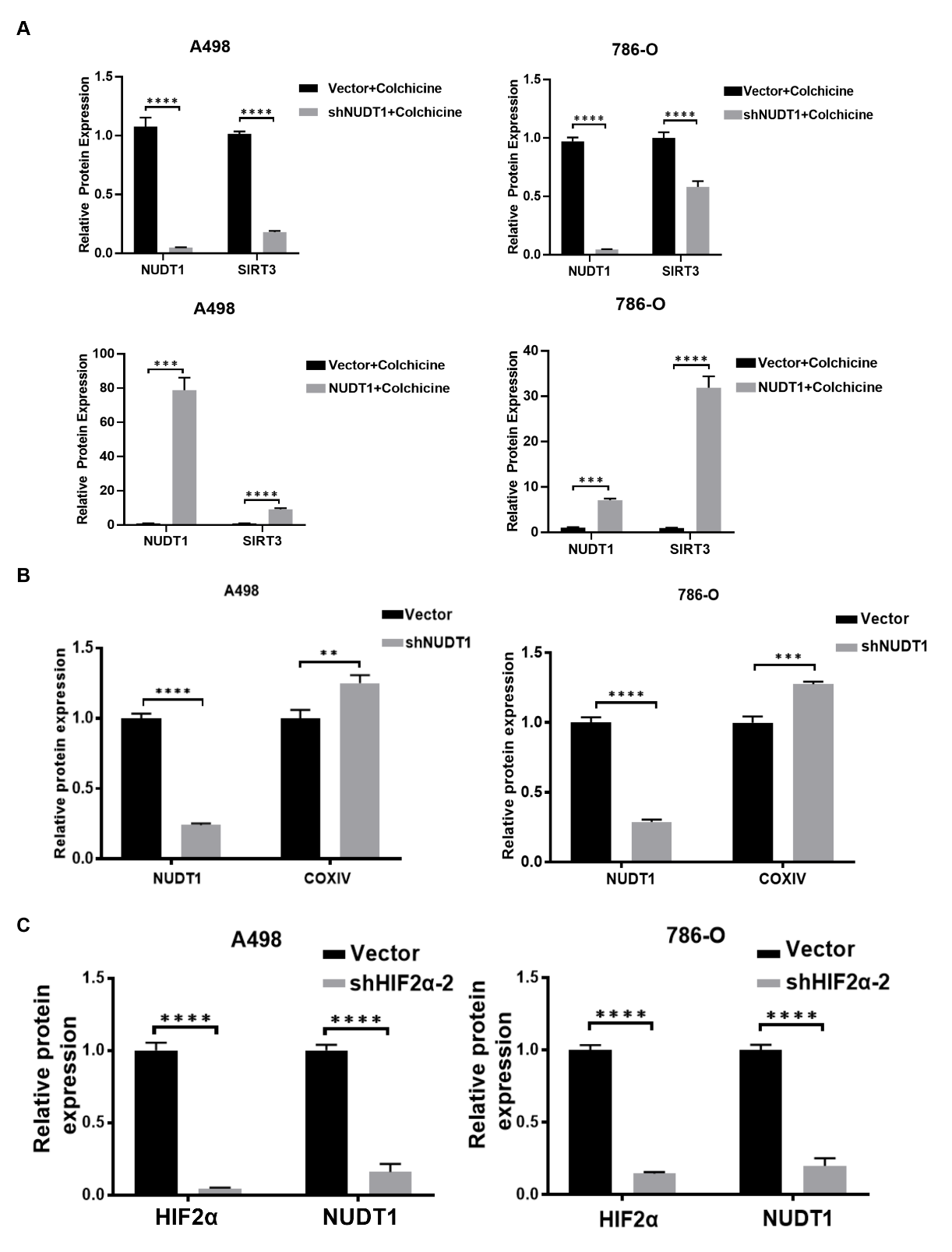


**Western blot statistics.** (A) Supplementary figure S8C. (B) Supplementary figure S8D. (C) Supplementary figure S11A.


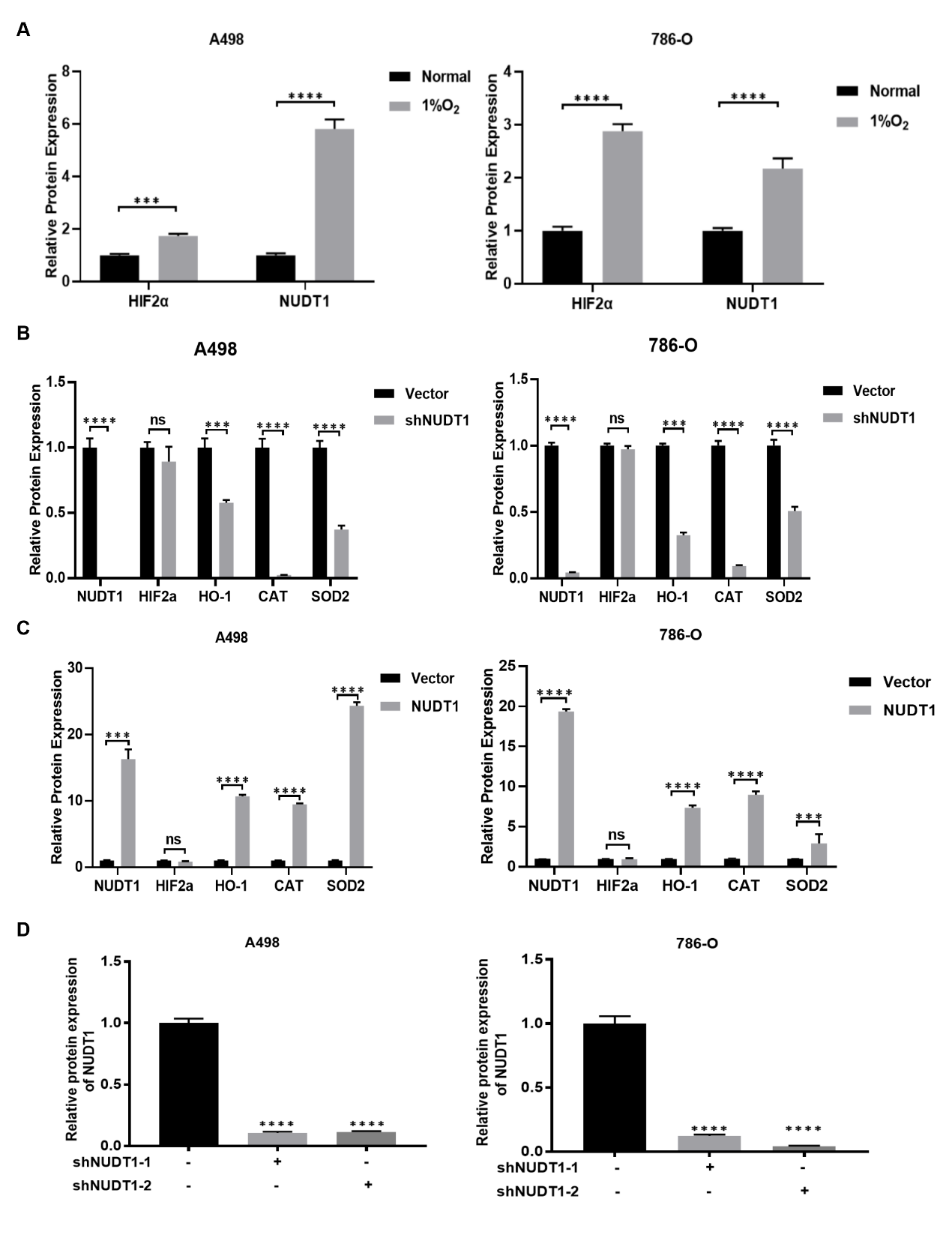


**Western blot statistics.** (A) Supplementary figure S11C. (B) Supplementary figure S11D. (C) Supplementary figure S11E. (D) Supplementary figure S15A.
