# Supplementary figures and images for: HIF2α promotes tumour growth in clear cell renal cell carcinoma by increasing the expression of NUDT1 to reduce oxidative stress
Source: Clin Transl Med. 2021 Nov 4;11(11):e592. doi: 10.1002/ctm2.592 (PMC8567048; doi:10.1002/ctm2.592)

**Supplementary information 3** Original blots of the western blotting


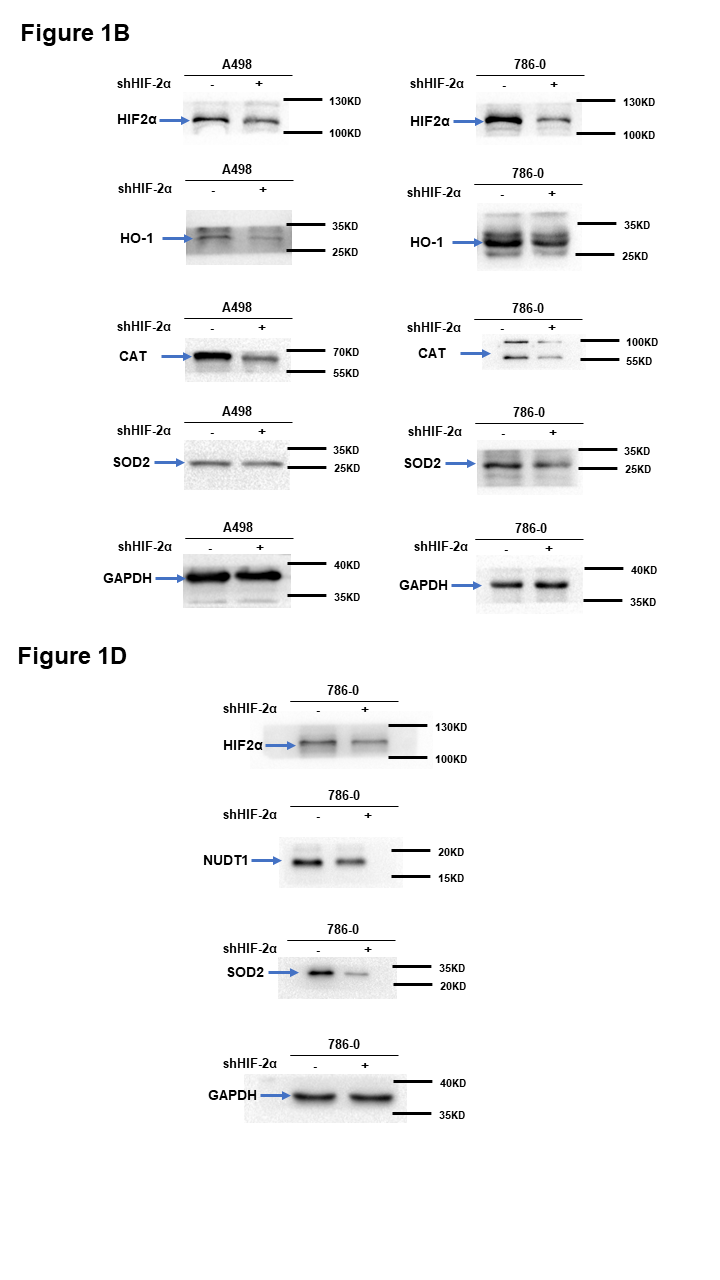

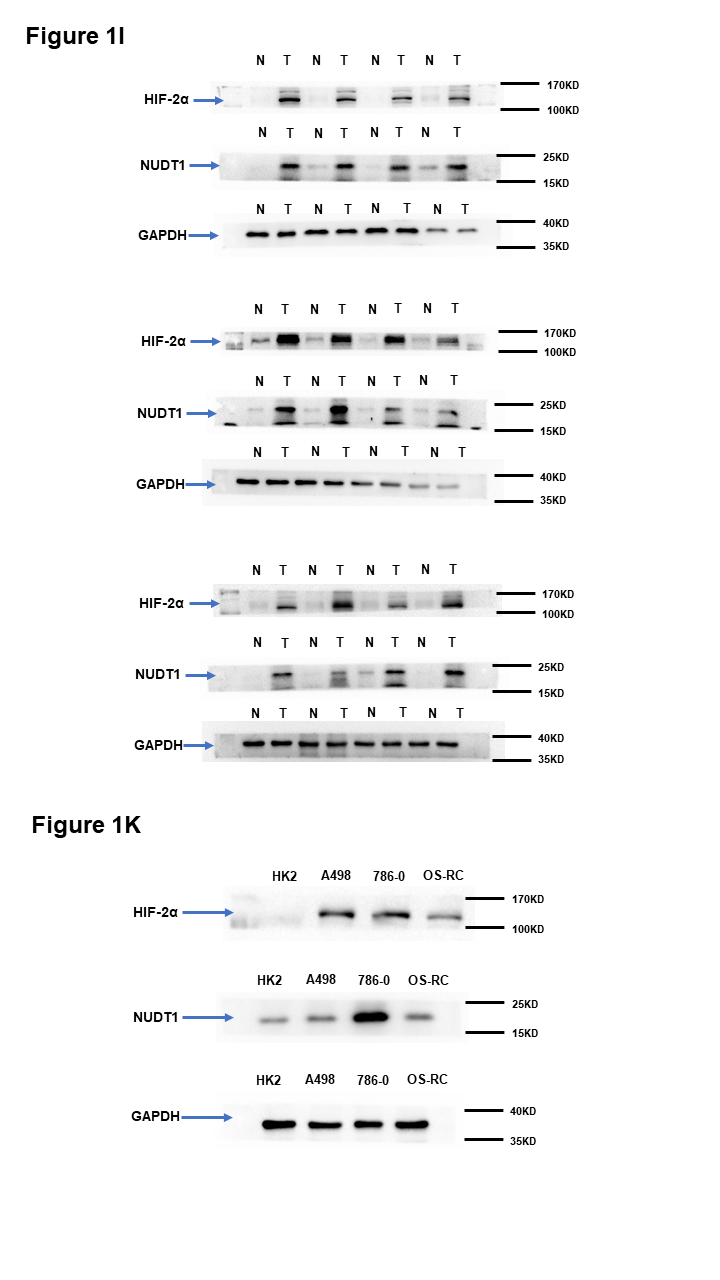

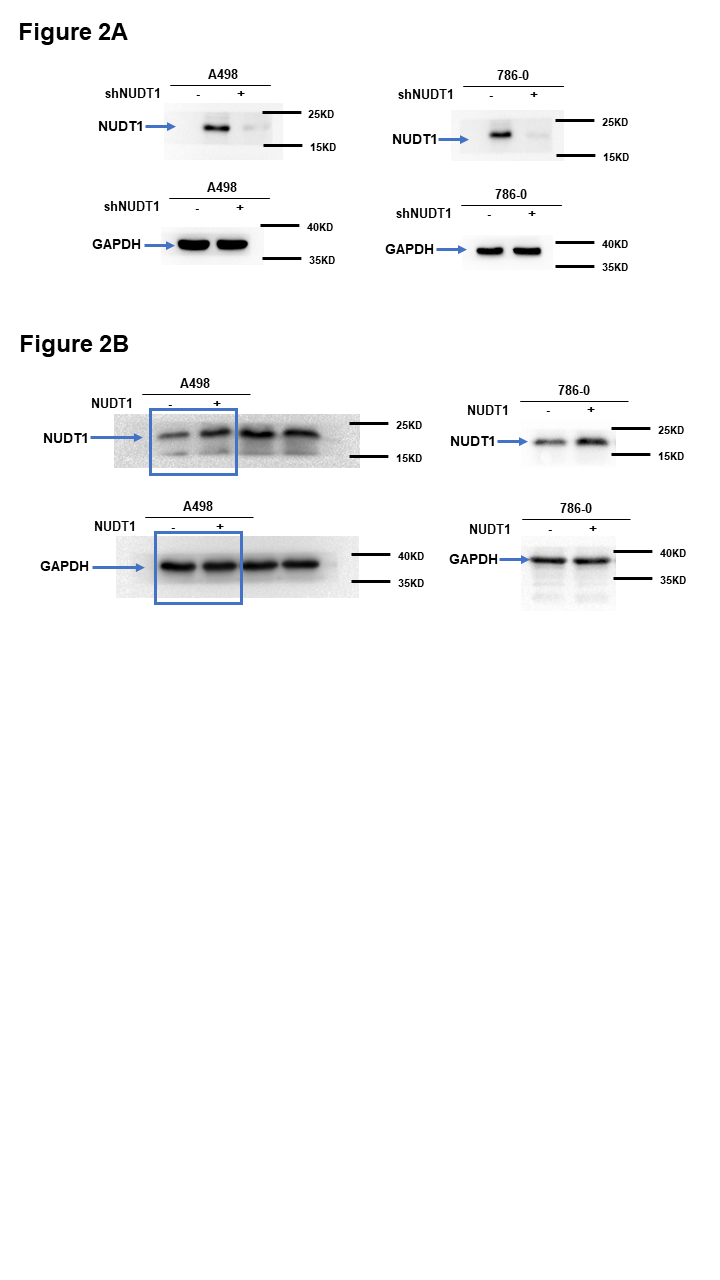

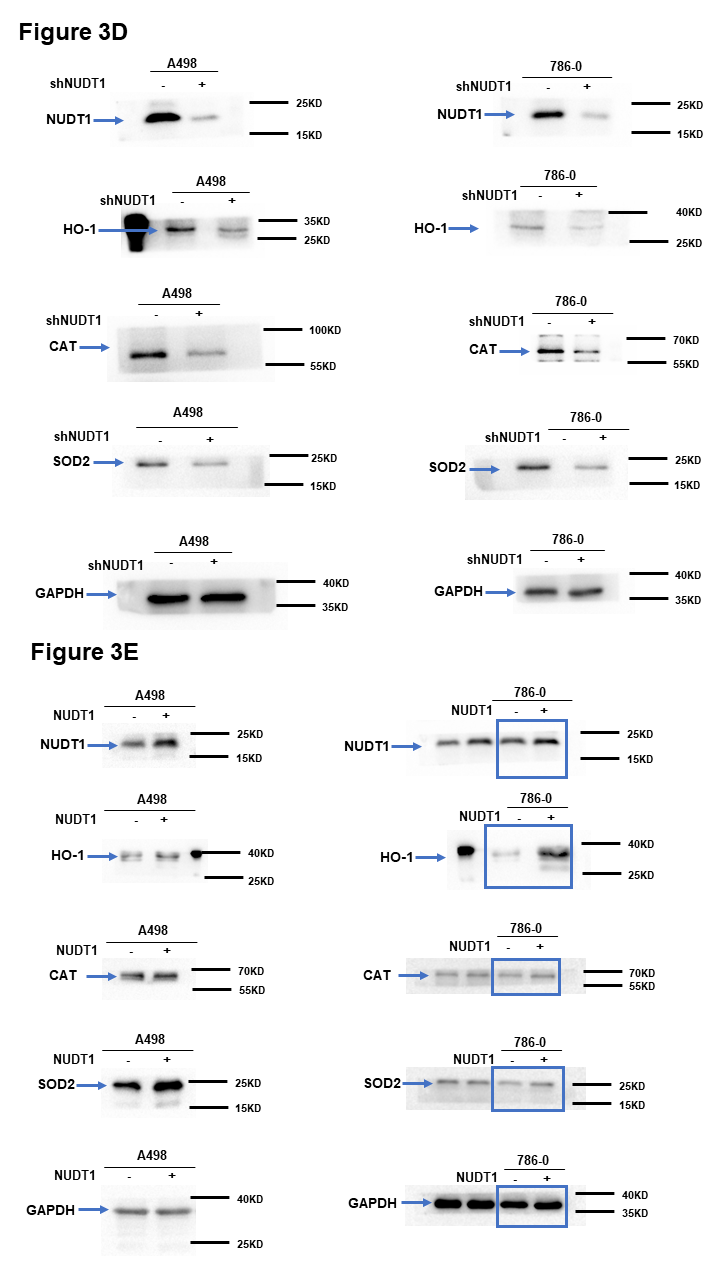

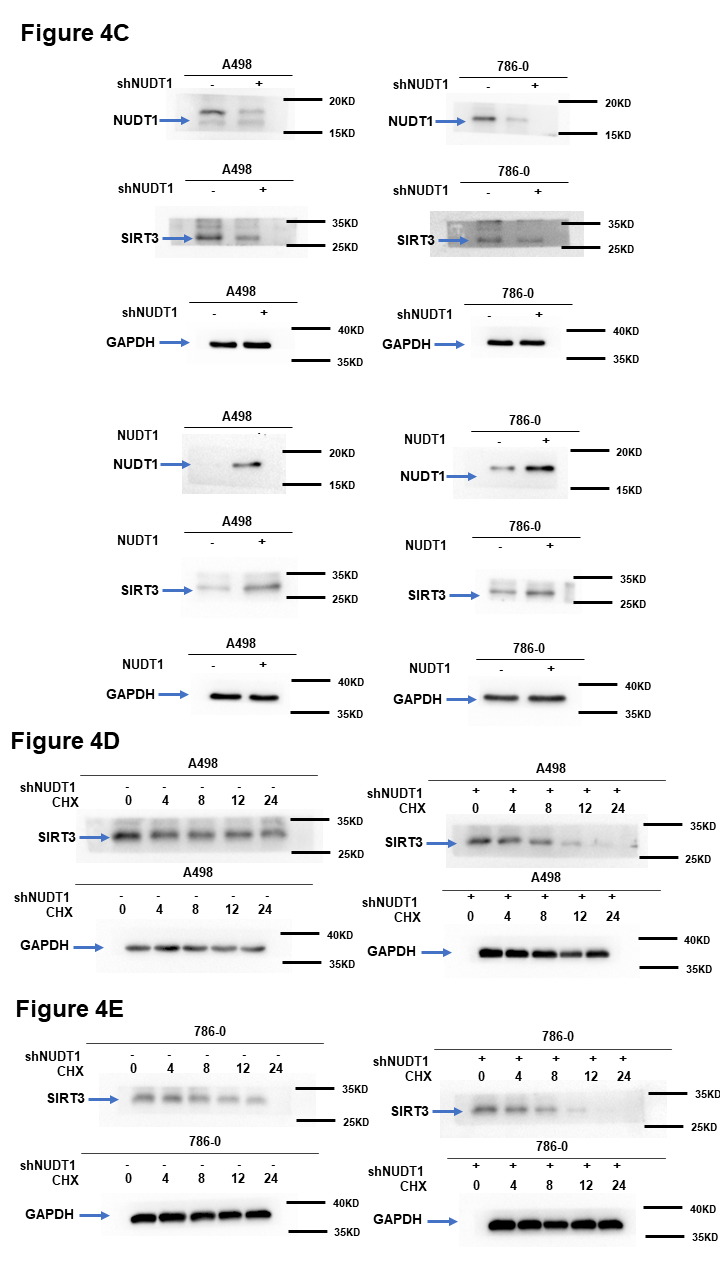

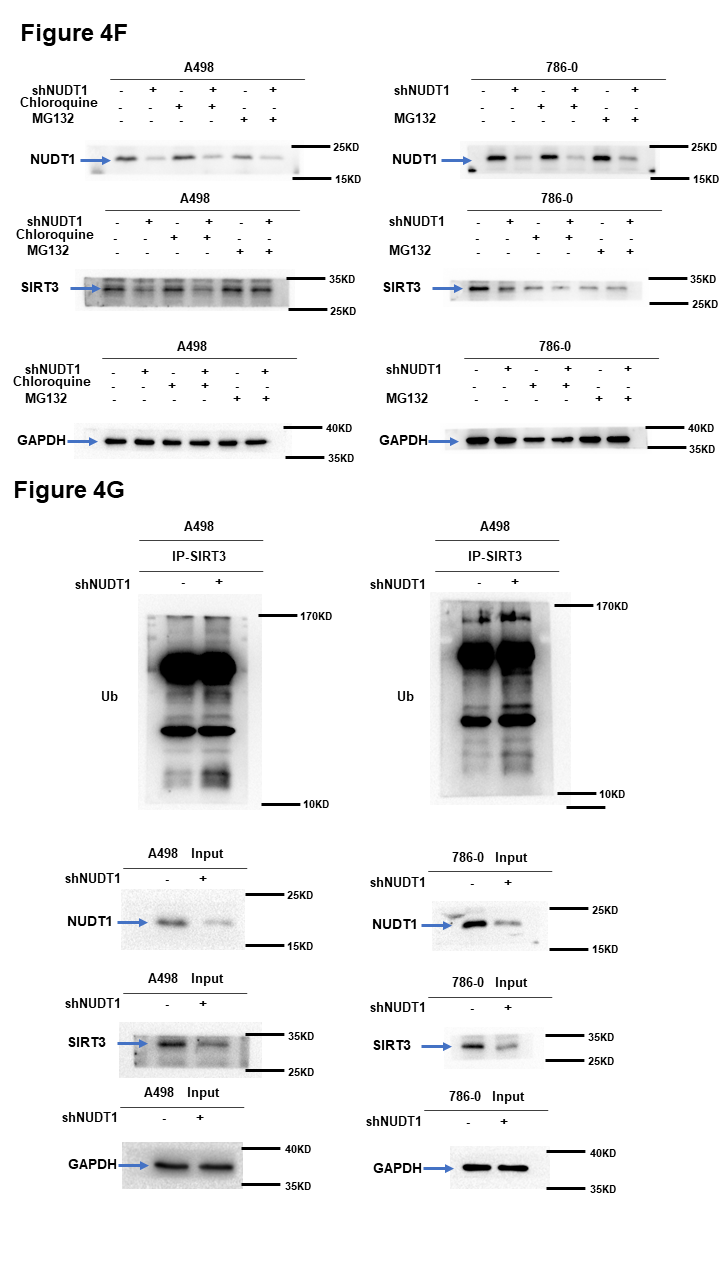

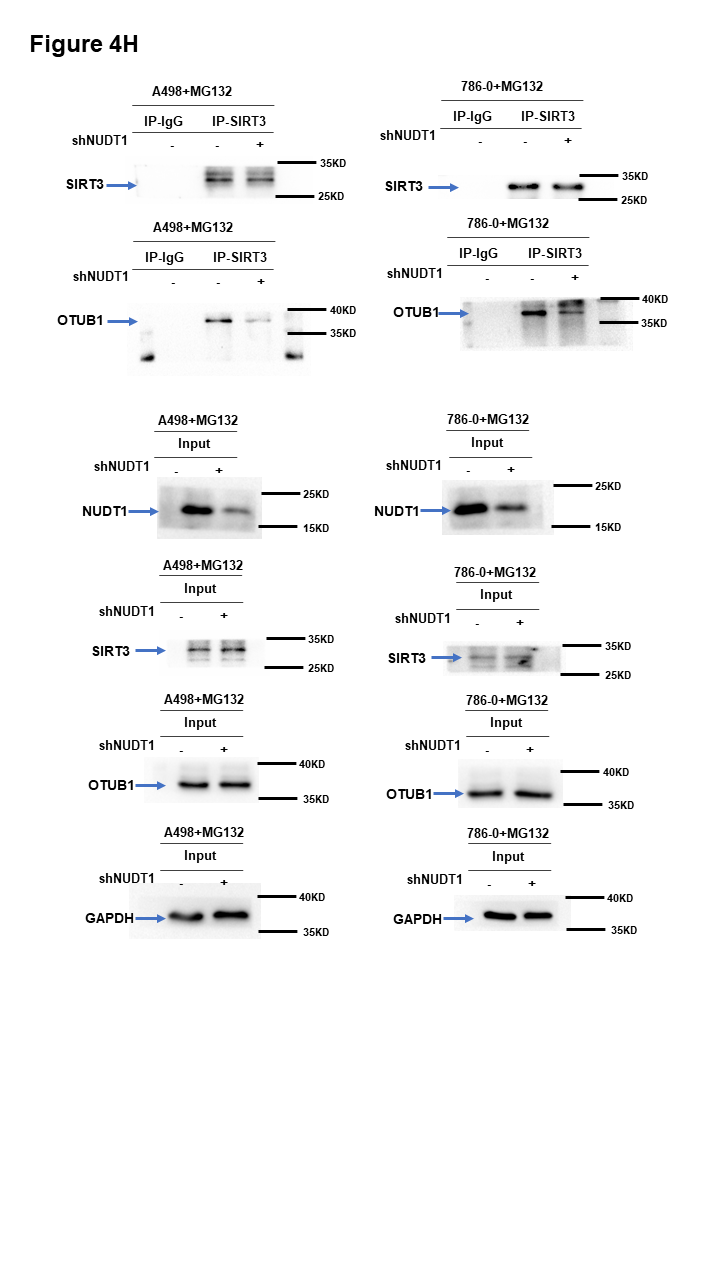


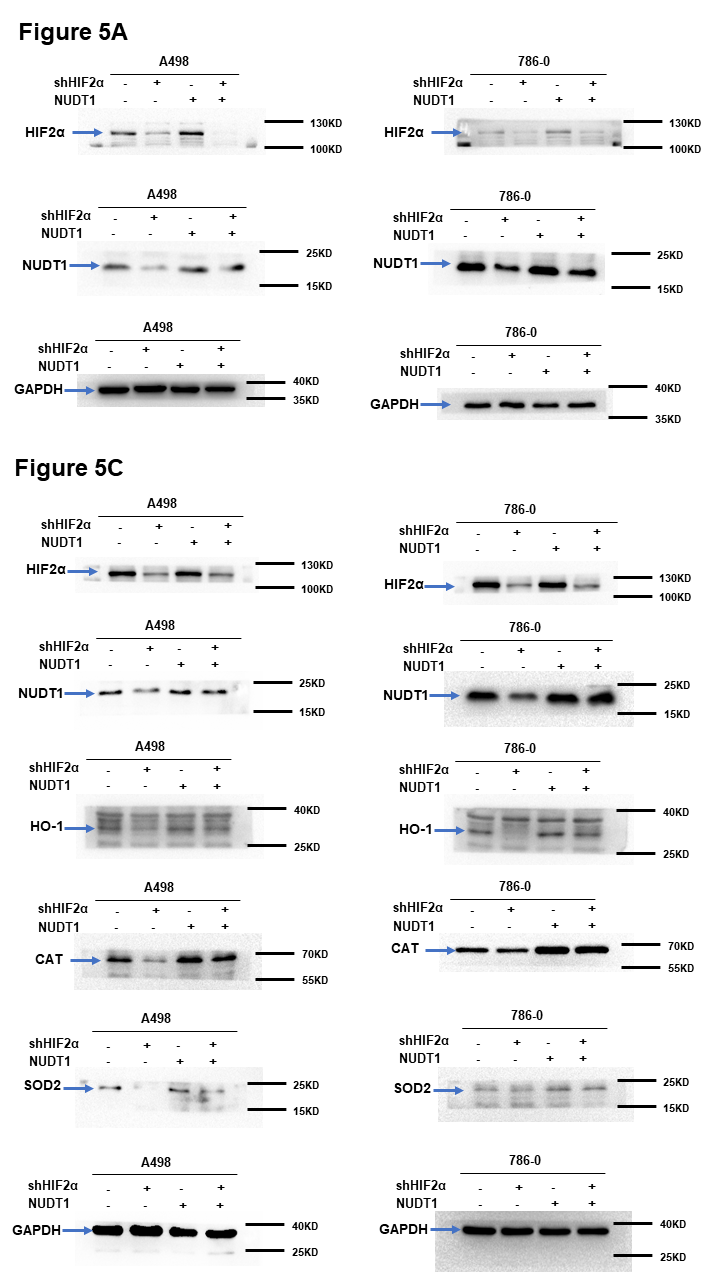


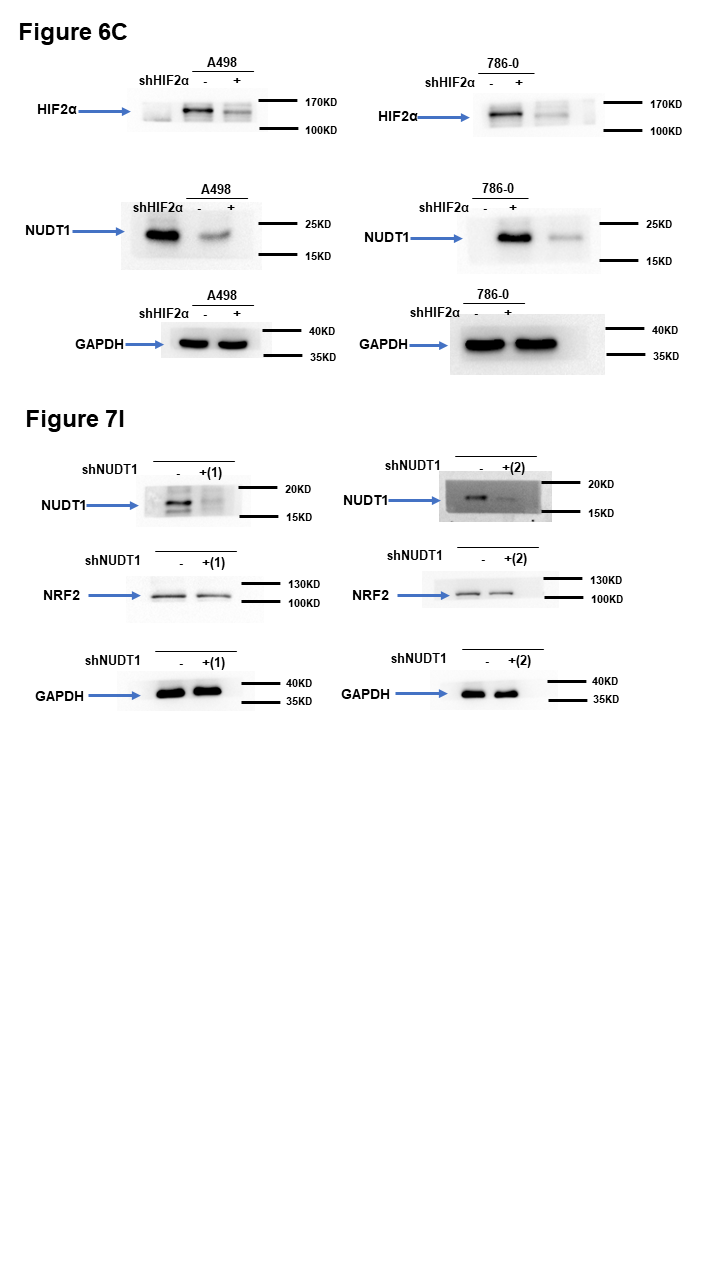


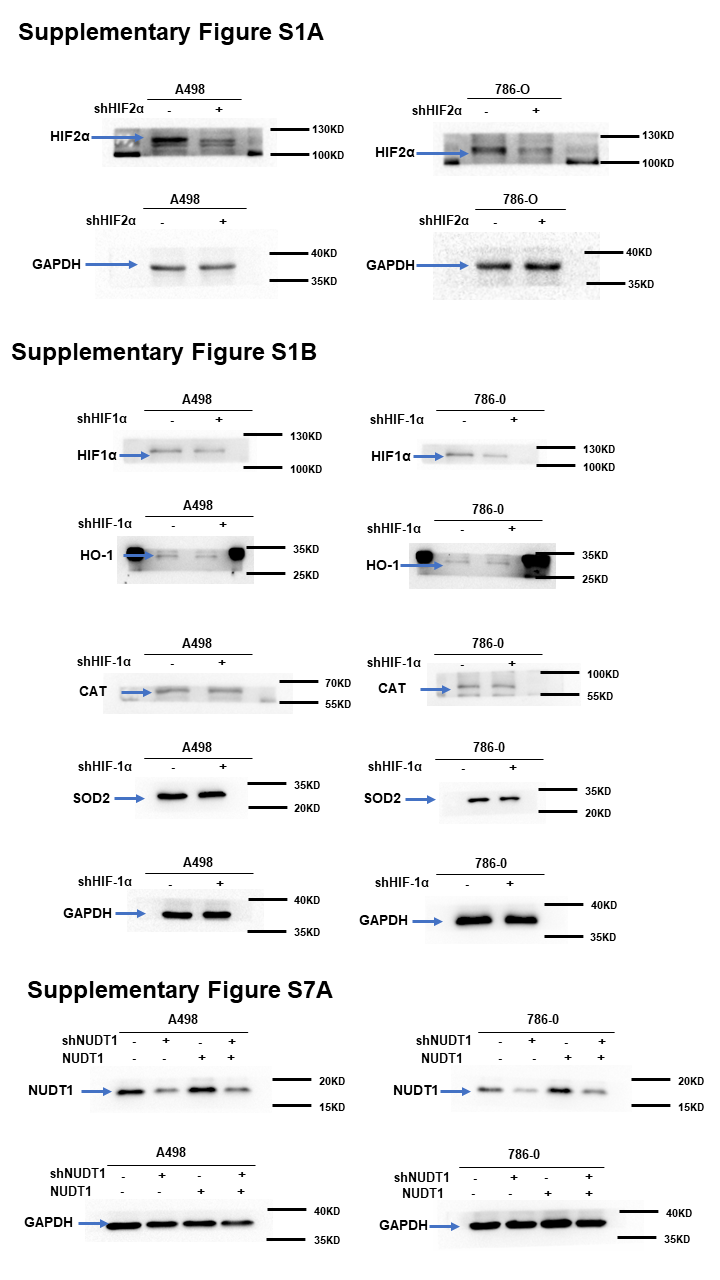


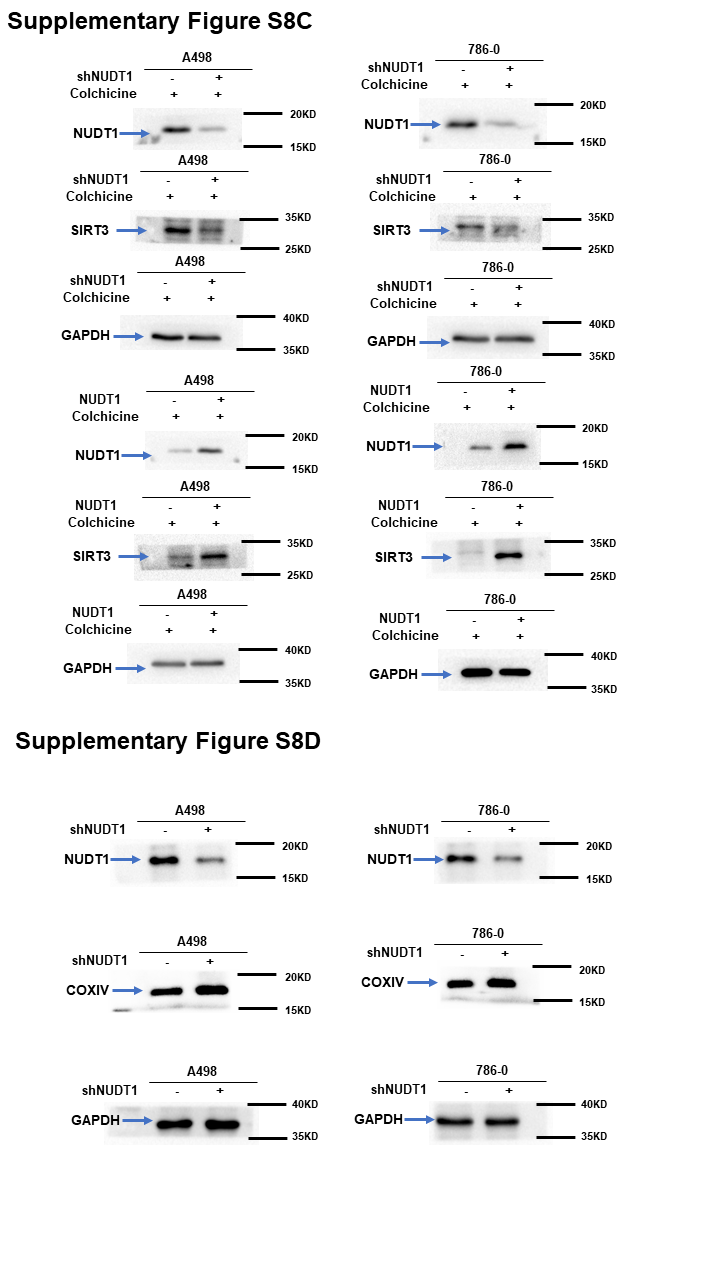


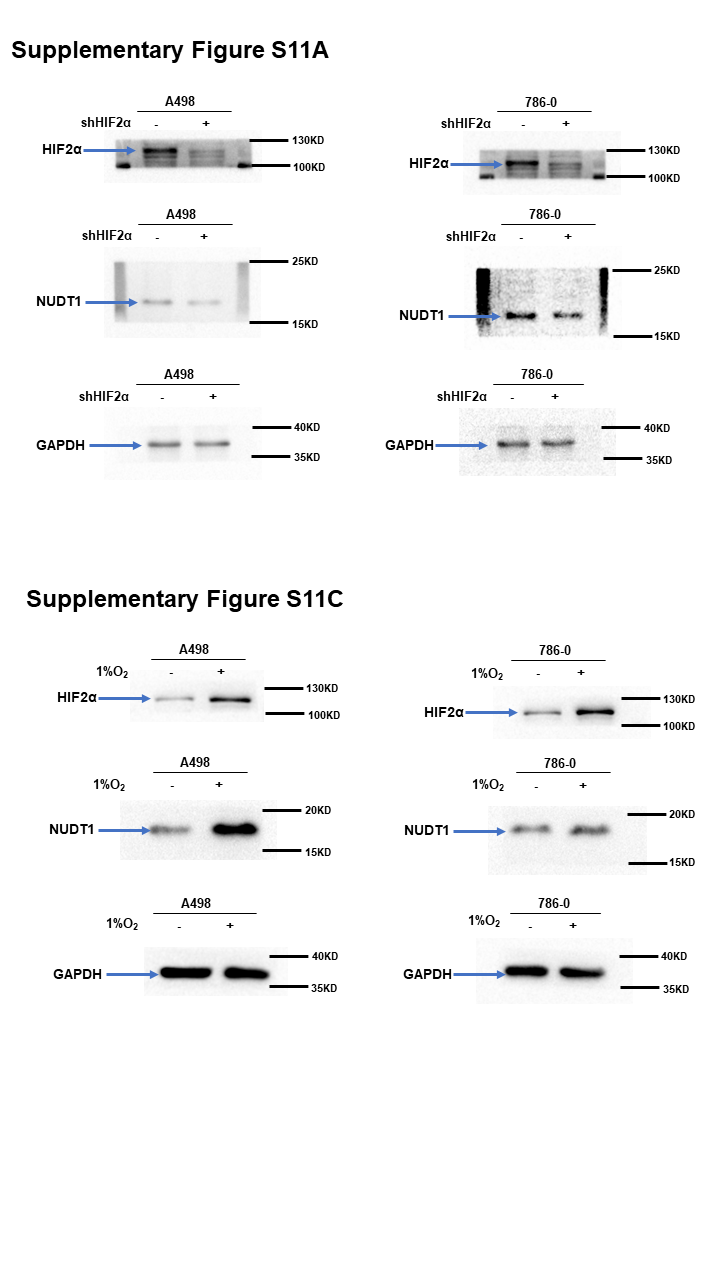


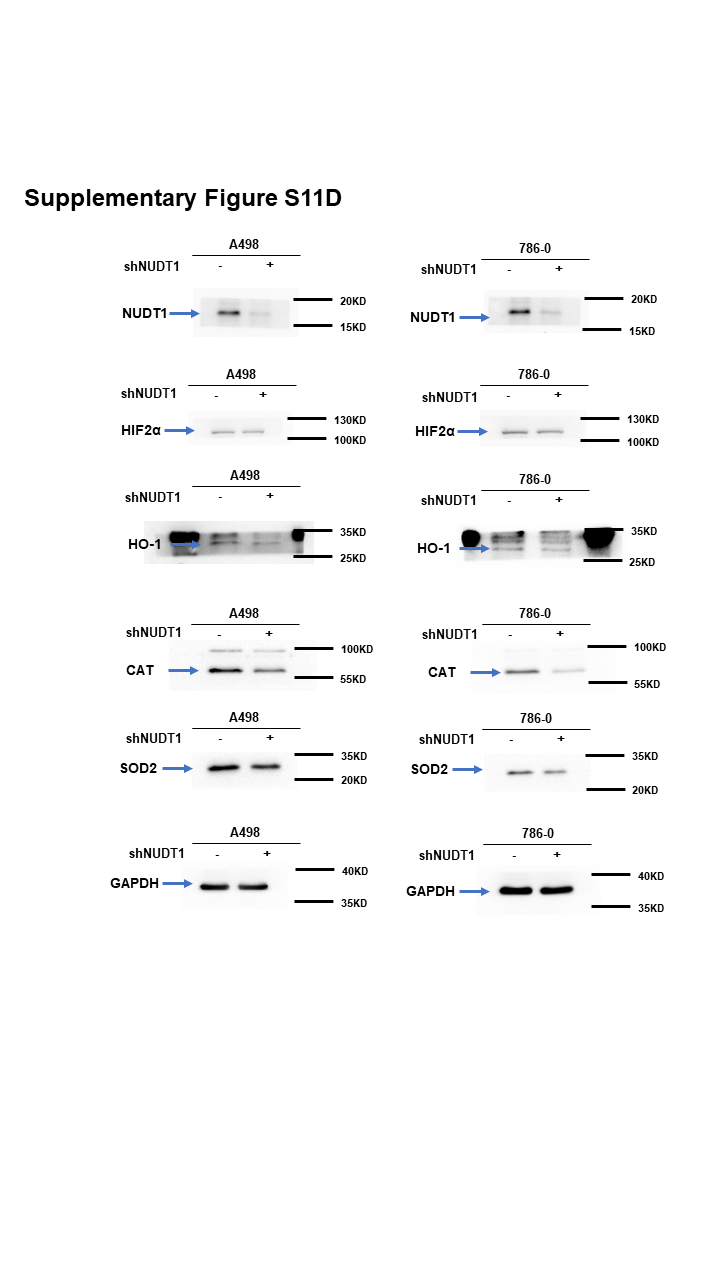


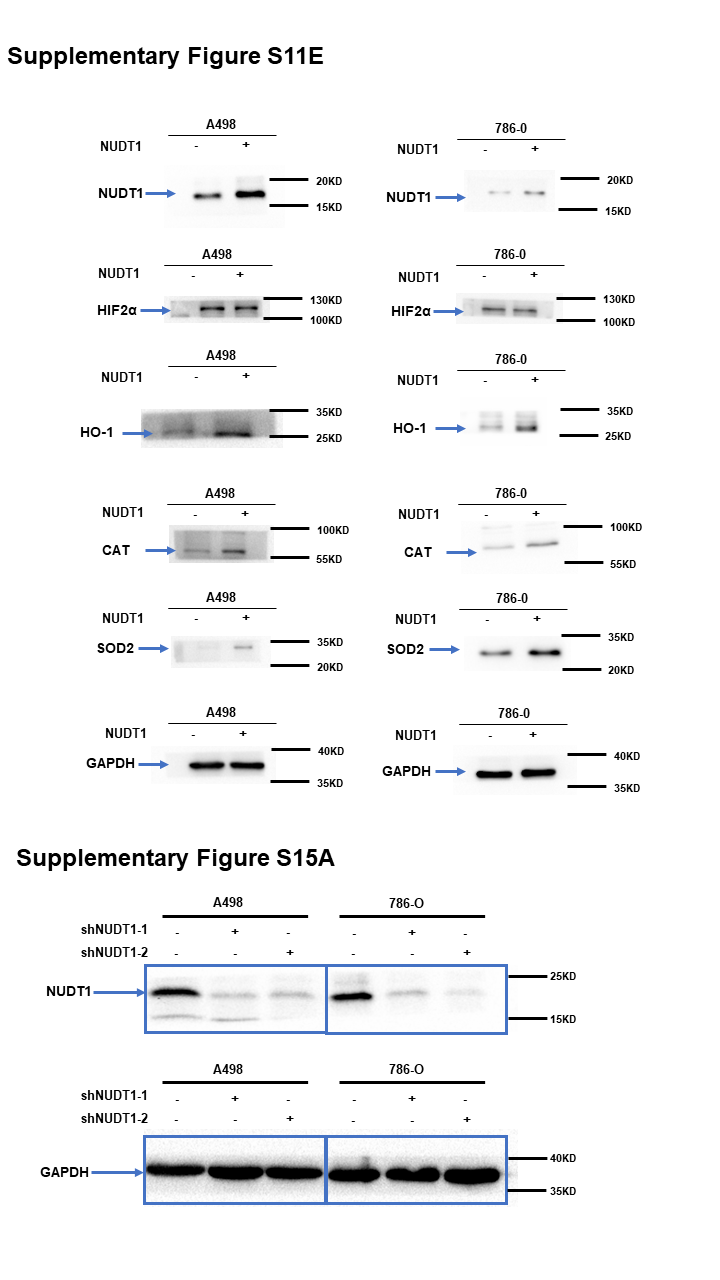

Supplement: Supplementary file 3 — Supplementary information 3 [file CTM2-11-e592-s001.docx]
