# Supplementary material for: HIF2α promotes tumour growth in clear cell renal cell carcinoma by increasing the expression of NUDT1 to reduce oxidative stress
Source: Clin Transl Med. 2021 Nov 4;11(11):e592. doi: 10.1002/ctm2.592 (PMC8567048; doi:10.1002/ctm2.592)
Supplement: Supplementary file 4 — Supplementary information 4 [file CTM2-11-e592-s002.docx]

**Supplementary information 4** Immunohistochemical quantification.


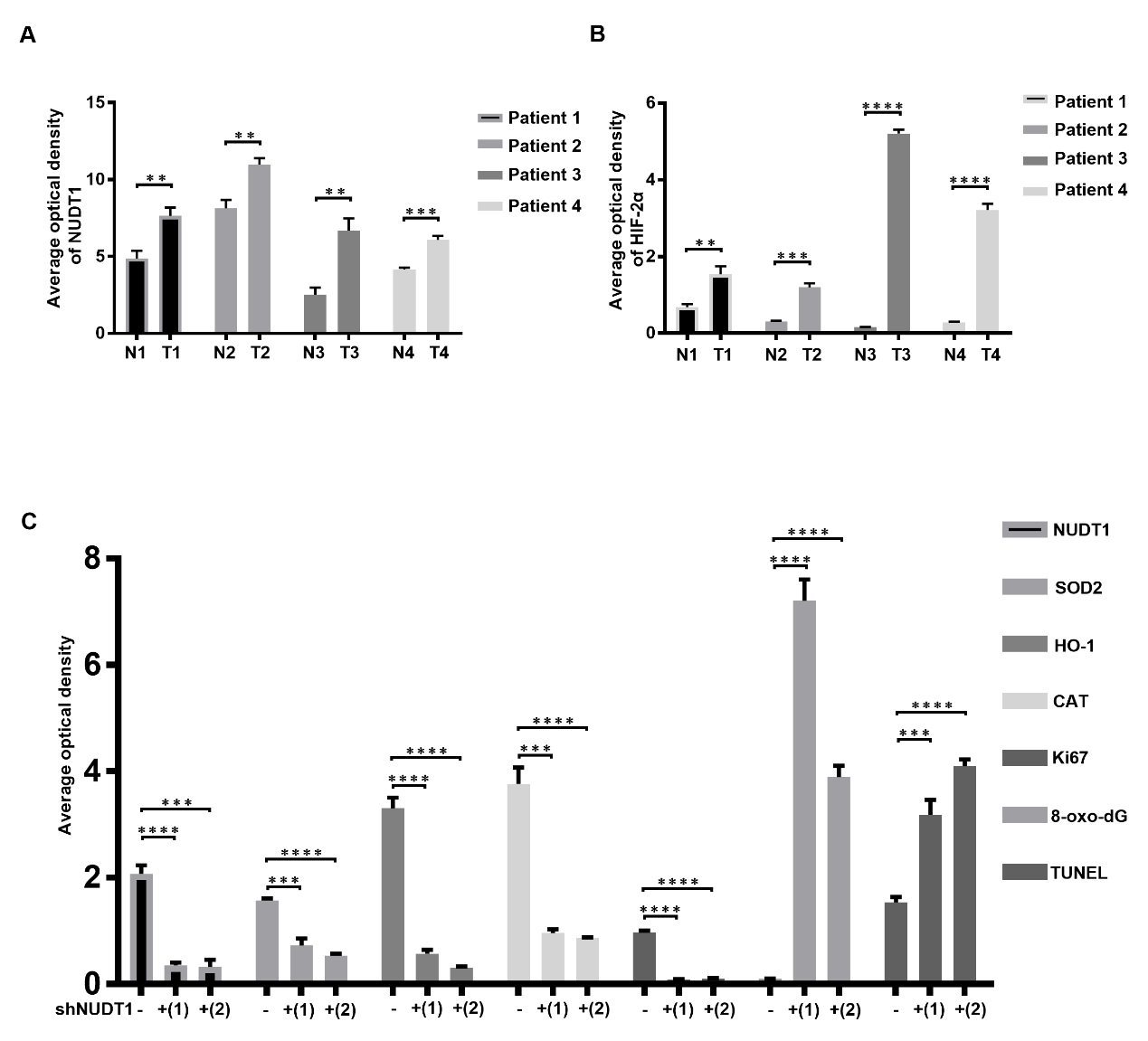


**A.** Immunohistochemical quantification of Figure 1J.

B. Immunohistochemical quantification of Supplementary figure S6.

C. Immunohistochemical quantification of Figure 7F.
